# An RNA‐Binding Protein RBMS1 Promotes Endothelial Cell Autophagy to Maintain Vascular Homeostasis and Suppress Deep Vein Thrombosis

**DOI:** 10.1002/advs.77393

**Published:** 2026-08-27

**Authors:** Chu Chu, Xinkui Liu, Nannan Fan, Xiaoyan Yu, Qiaoqiao Han, Yunhong Zhang, Wei Li, Yongqing Cai, Miaomiao Zhou, Fang Li, Feifei Shi, Zhen Zhang, Bin Wang, Xia Li

**Affiliations:** ^1^ Innovative Institute of Chinese Medicine and Pharmacy Shandong University of Traditional Chinese Medicine Jinan China; ^2^ Key Laboratory of Traditional Chinese Medicine Classical Theory Jinan Key Laboratory of Traditional Chinese Medicine Immunoregulation Ministry of Education Traditional Chinese Medicine Immunoregulation Engineering Research Center of Shandong Province Shandong University of Traditional Chinese Medicine Jinan China; ^3^ Shanghai Institute of Immunology State Key Laboratory of Systems Medicine for Cancer Shanghai Jiao Tong University School of Medicine Shanghai China; ^4^ The Second Affiliated Hospital of Shandong University of Traditional Chinese Medicine Jinan China; ^5^ College of Traditional Chinese Medicine Shandong University of Traditional Chinese Medicine Jinan China

**Keywords:** ATG3, autophagy, deep venous thrombosis (DVT), RBMS1, vascular endothelial cells (VECs)

## Abstract

Deep vein thrombosis (DVT) is a prevalent vascular disorder characterized by aberrant coagulation within the deep venous system, and the roles of RNA‐binding proteins (RBPs) in its pathogenesis remain largely undefined. Herein, using a murine inferior vena cava (IVC) stenosis model, we identify RNA‐binding motif single‐stranded interacting protein 1 (RBMS1) as a previously unrecognized regulator of DVT progression via the modulation of vascular endothelial cell (VEC) autophagy. By utilizing endothelial cell‐conditional *Rbms1* knockout mice, we demonstrate that RBMS1 deficiency exacerbates thrombus formation and impairs VEC function. Mechanistically, RBMS1 associates with autophagy‐related 3 (ATG3) mRNA through its 3′‐untranslated region (3′‐UTR) and enhances its stability, thereby promoting autophagic flux in VECs. *RBMS1* depletion destabilizes *ATG3* mRNA, leading to suppressed autophagy and compromised endothelial homeostasis. Conversely, ATG3 overexpression rescues autophagy impairment and mitigates thrombotic phenotypes upon *RBMS1* depletion. Collectively, our findings in the IVC stenosis‐induced DVT model demonstrate that RBMS1 regulates ATG3 to maintain vascular endothelial integrity, providing a mechanistic basis for further evaluation of RBMS1 in DVT intervention.

## Graphical abstract

RBMS1, an RNA‐binding protein, restores vascular endothelial homeostasis by enhancing ATG3‐mediated autophagy, thereby attenuating thrombus formation and suggesting a potential avenue for further therapeutic exploration in DVT.

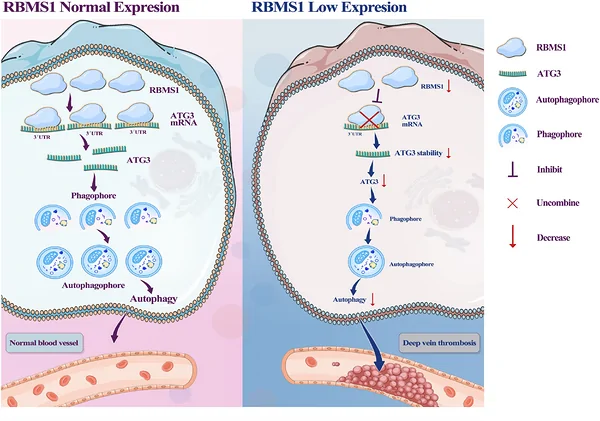

## 1 Introduction

Deep vein thrombosis (DVT) is a common and serious peripheral vascular disease characterized by the pathological formation of blood clots within deep veins, leading to venous lumen obstruction and impaired return flow [1]. With an estimated 10 million new cases diagnosed annually worldwide, DVT ranks as the third most prevalent vascular disorder following acute myocardial infarction and stroke, imposing a significant global health burden [2, 3]. Current established therapeutic strategies, primarily anticoagulation and thrombolysis, exhibit limited efficacy and are associated with considerable risks, including hemorrhage, allergic reactions, and hepatic or renal dysfunction [4, 5]. Notably, 20% to 50% of patients develop postthrombotic syndrome after treatment, which severely compromises their long‐term quality of life [6, 7]. Therefore, elucidating the molecular pathogenesis of DVT is imperative for the development of novel, effective therapeutic targets and intervention strategies.

Vascular endothelial cells (VECs), which constitute the primary biological interface of the vessel wall, play central roles in maintaining vascular homeostasis by regulating vasomotor tone, coagulation‐anticoagulation balance, and inflammatory responses [8, 9, 10]. Endothelial dysfunction is recognized as a central initiating event in DVT pathogenesis, yet the upstream regulatory mechanisms remain incompletely understood [11]. Autophagy, a highly conserved cellular self‐degradative process, is essential for maintaining intracellular homeostasis through the clearance of damaged organelles and misfolded proteins [12, 13]. Autophagy‐related 3 (ATG3), a key ubiquitin‐like conjugating enzyme, facilitates autophagosome membrane elongation by catalyzing the lipidation of microtubule‐associated protein 1 light chain 3 (LC3)‐I to form LC3‐II [14, 15]. While ATG3 has been implicated in atherosclerosis and myocardial injury [16, 17], its functional significance in DVT is still unknown.

RNA‐binding proteins (RBPs) are master regulators of posttranscriptional gene expression, governing messenger RNA (mRNA) splicing, stability, localization, and translation [18, 19, 20]. RBP dysregulation is increasingly linked to various human diseases, including cancer and vascular pathologies [21, 22, 23, 24]. RNA‐binding motif single‐stranded interacting protein 1 (RBMS1), initially identified as a c‐Myc single‐stranded deoxyribonucleic acid (DNA) binding protein [25], has emerged as a multifaceted regulator that influences RNA stability and translation in various biological contexts. For instance, in triple‐negative breast cancer, RBMS1 stabilizes B4GALT1 mRNA to promote programmed death‐ligand 1 protein glycosylation protein glycosylation and immune evasion [23]; similarly, in non‐small cell lung cancer, it activates S100P translation in an N6‐methyladenosine (m6A)‐dependent manner to facilitate metastasis [22]. Despite these advances in oncology, the role of RBMS1 in vascular biology, particularly in the pathogenesis of DVT, remains unexplored.

The aim of this study was to investigate the function and mechanism of RBMS1 in DVT pathogenesis. We demonstrate that RBMS1 expression is downregulated in DVT and that its conditional deletion in endothelial cells aggravates thrombosis. Mechanistically, we identify ATG3 as a downstream target, with RBMS1‐binding to its 3'‐UTR to increase mRNA stability and promote autophagic flux in VECs. Our findings establish a novel regulatory mechanism in vascular endothelium maintenance, offering insights into DVT pathogenesis and highlighting a potential therapeutic candidate for further investigation.

## 2 Results

### 2.1 The Downregulation of RBMS1 Expression in Vascular Endothelial Cells Exacerbates DVT

**FIGURE 1:**
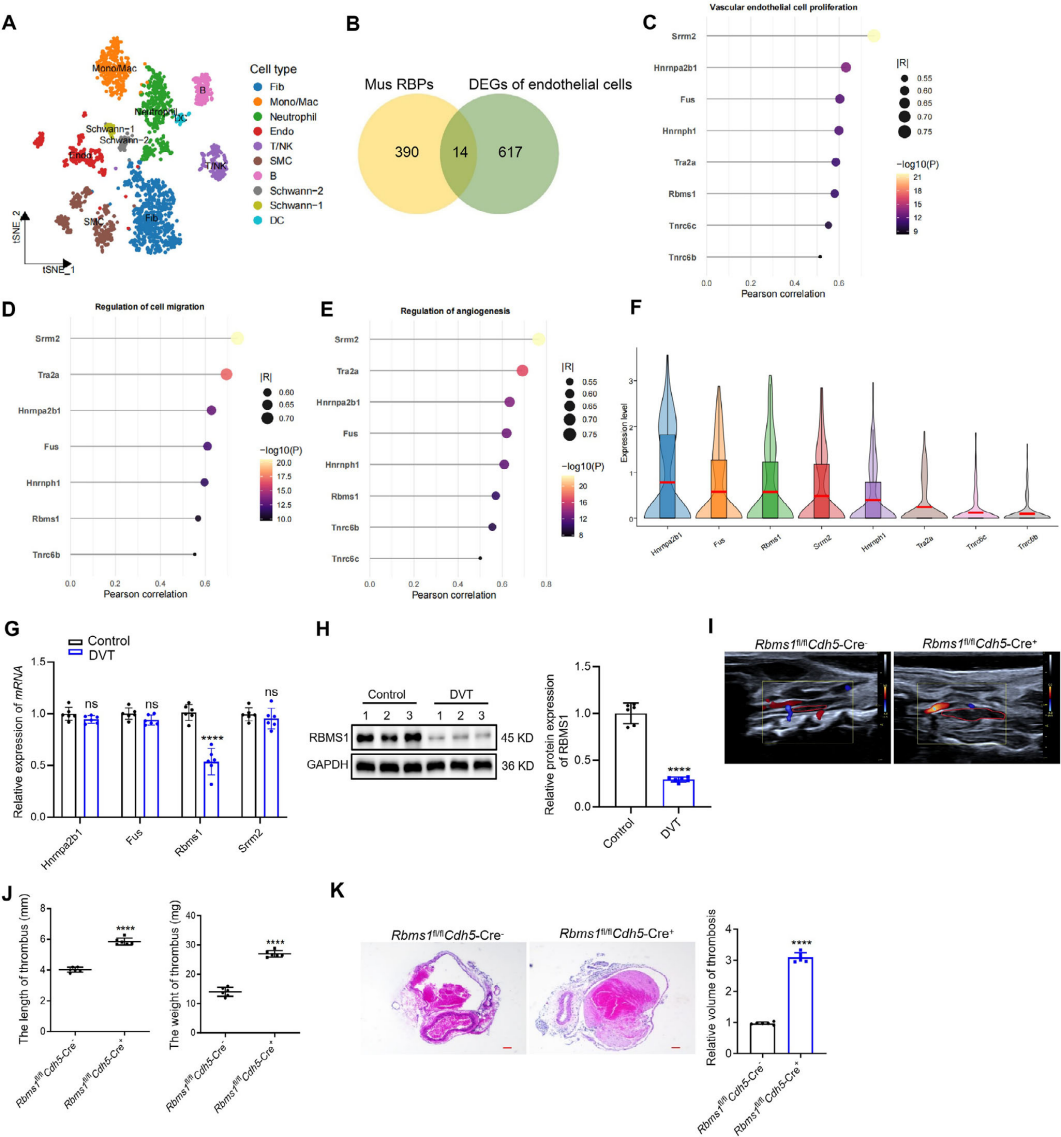
The downregulation of RBMS1 expression in vascular endothelial cells exacerbates DVT. (A) t‐SNE visualization showing the cellular composition of the DVT microenvironment. (B) Venn diagram showing 14 significantly altered RNA‐binding proteins (RBPs) in endothelial cells from DVT model mice. (C–E) Correlation analysis of RBP expression with endothelial functional signatures: proliferation (C), migration (D), and angiogenesis (E). (F) Expression levels of four candidate RBPs (*Hnrnpa2b1*, *Fus*, *Rbms1*, and *Srrm2*) in endothelial cells. (G) qRT‐PCR analysis of the mRNA expression of the four RBPs in endothelial cells isolated from DVT model mice and control mice (*n* = 6 per group). (H) Representative Western blot images of RBMS1 and GAPDH in endothelial cells from DVT model mice and control mice (*n* = 6 per group). (I) Representative murine Doppler ultrasound images of thrombi in control and *Rbms1*‐knockout DVT model mice. (J) Quantification of thrombus weight and length in the indicated groups (*n* = 6 per group). (K) H&E‐stained sections of thrombi from control and Rbms1‐knockout mice (magnification: × 40, scale bar = 100 µm). The data are presented as the mean± SEM. ^****^ *p* < 0.0001 by unpaired Student's t‐test. n represents the number of individual mice.

Although RNA‐binding proteins (RBPs) have been well documented in cardiovascular diseases [26, 27, 28], their role in DVT remains poorly understood. To systematically explore the function of RBPs in DVT, we analyzed single‐cell RNA sequencing data (GSE221978) from a mouse model of DVT. We applied t‐distributed stochastic neighbor embedding (t‐SNE), a non‐linear dimensionality reduction method that preserves local structures in high‐dimensional data, facilitating the visualization of distinct cell populations, to decipher the cellular composition of the DVT microenvironment (Figure 1A). With respect to endothelial cells, we identified genes whose expression was dysregulated in DVT compared with controls, and by intersecting these differentially expressed genes with a comprehensive murine RBP dataset, we identified 14 RBPs whose expression was significantly altered in endothelial cells from DVT model mice (Figure 1B). To prioritize the RBPs potentially involved in DVT pathogenesis, we performed correlation analysis between the expression of these 14 RBPs and the functional signatures of endothelial cells, including proliferation, migration, and angiogenesis (Figure 1C–E). This analysis highlighted eight RBPs that correlated strongly with endothelial functional phenotypes, suggesting their potential role in regulating endothelial homeostasis.

Among these candidates, *Hnrnpa2b1*, *Fus*, *Rbms1*, and *Srrm2* exhibited the highest expression levels in endothelial cells and were selected for further validation (Figure 1F). Quantitative real‐time polymerase chain reaction (qRT‐PCR) analysis of endothelial cells isolated from DVT mice confirmed the dysregulation of these genes, with *Rbms1* showing the most pronounced downregulation (Figure 1G). Western blotting validated the reduction in the level of the RBMS1 protein in DVT samples (Figure 1H). To determine whether RBMS1 influences thrombus formation, we generated endothelial cell‐conditional *Rbms1* knockout mice by crossing *Rbms1* ^flox/flox^ mice with *Cdh5*‐Cre mice. To verify the endothelial specificity of *Cdh5*‐Cre‐mediated recombination and exclude potential off‐target effects that could confound the thrombus phenotype, we isolated VECs, non‐VECs in the vascular tissues, and peripheral CD45^+^ immune cells from *Rbms1* ^fl/fl^ *Cdh5*‐Cre^+^ mice and their Cre^−^ littermates. Western blotting confirmed that RBMS1 was efficiently depleted only in VECs from Cre^+^ mice (Figure S1A), whereas its levels remained unchanged in non‐VECs (Figure S1B) and CD45^+^ cells (Figure S1C). Murine Doppler ultrasound imaging revealed significantly greater thrombus volume in endothelial cell‐conditional *Rbms1* knockout mice than in control mice (Figure 1I). Similarly, *Rbms1* deficiency led to a marked increase in both thrombus length and weight (Figure 1J). Hematoxylin and Eosin (H&E) staining further corroborated these observations, revealing substantially expanded thrombotic masses in conditional *Rbms1* knockout mice (Figure 1K). Together, these results established RBMS1 as a previously unrecognized regulator of DVT pathogenesis.

### 2.2 RBMS1 Serves as a Positive Regulator of VEC Function

**FIGURE 2:**
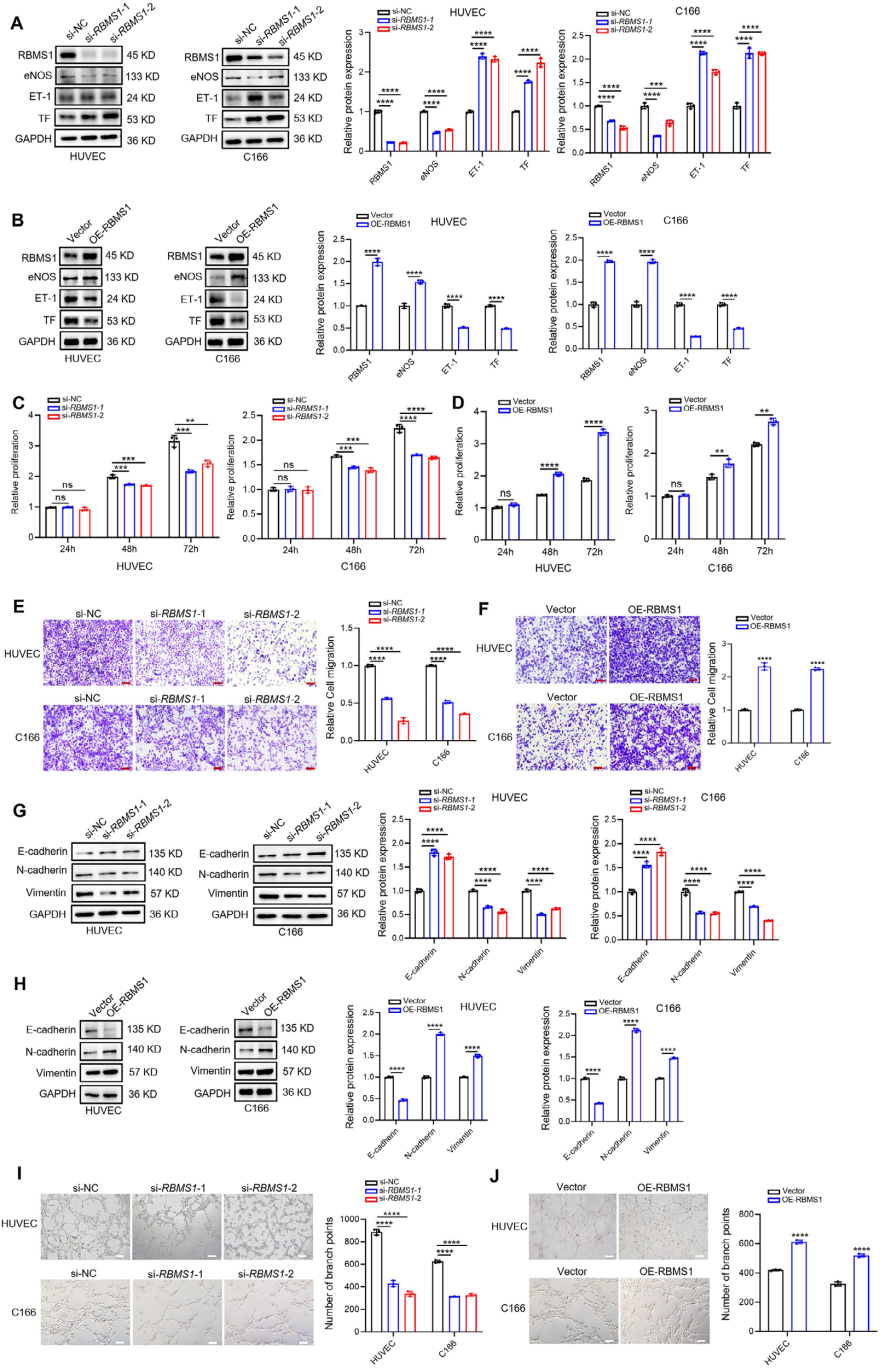
RBMS1 serves as a positive regulator of VEC function. (A, B) Western blot analysis showing the effects of RBMS1 knockdown and overexpression on endothelial function‐related proteins in HUVECs and C166 cells (*n* = 3). (C, D) Cell growth curves were determined using CCK‐8 assays following transfection with si‐RBMS1 or OE‐RBMS1 (*n* = 3). (E, F) Cell migration capacity was analyzed using Transwell assays. Representative images of cells that migrated through the porous membrane (crystal violet staining, magnification: × 100, scale bar = 100 µm) (*n* = 3). (G, H) Western blot analysis showing the effects of RBMS1 knockdown and overexpression on E‐cadherin, N‐cadherin, and vimentin expression in HUVECs and C166 cells (*n* = 3). (I, J) Representative bright‐field images of tube structures formed by HUVECs and C166 cells transfected with si‐*RBMS1* or OE‐RBMS1 on Matrigel (magnification: × 100, scale bar = 100 µm) and quantitative analysis of the number of branch points per field. *n* = 3 represents three independent biological replicates obtained from three independently performed experiments. Individual data points are shown, and data are presented as the mean± SEM. For panels A, C, E, G, and I, statistical significance was determined by one‐way ANOVA followed by Tukey′s multiple‐comparisons test; for panels B, D, F, H, and J, statistical significance was determined by unpaired Student's t‐tests. ^**^ *p* < 0.01, ^***^ *p* < 0.001, and ^****^ *p* < 0.0001. ns, not significant.

To elucidate the specific role of RBMS1 in the function of VECs, human umbilical vein endothelial cells (HUVECs) and C166 cells were transfected with either RBMS1‐targeting siRNA (si‐RBMS1) or an RBMS1 overexpression plasmid (OE‐RBMS1), with scrambled siRNA (si‐NC) or an empty vector as controls. After 48 h of transfection, a comprehensive evaluation was conducted to assess the influence of RBMS1 on cellular proliferation, migration, and tube‐forming capacity, and the expression of associated proteins. Western blot analysis revealed that RBMS1 knockdown resulted in the upregulation of endothelin‐1 (ET‐1) and tissue factor (TF) protein expression concomitant with the downregulation of endothelial nitric oxide synthase (eNOS) protein expression, whereas RBMS1 overexpression elicited the opposite effects. These findings position RBMS1 as a protective modulator in maintaining the homeostasis of VECs (Figure 2A, B). Subsequent Cell Counting Kit‐8 (CCK‐8) assays demonstrated that the suppression of RBMS1 expression significantly impaired the proliferative potential of VECs, whereas the overexpression of RBMS1 increased cellular proliferation (Figure 2C, D).

Transwell migration assays further revealed that RBMS1 knockdown markedly attenuated the migratory capacity of VECs, whereas increased RBMS1 levels promoted it (Figure 2E, F). Consistent with these observations, Western blot analysis revealed that RBMS1 knockdown increased E‐cadherin expression while reducing the levels of N‐cadherin and vimentin, and the opposite effects were observed after RBMS1 overexpression (Figure 2G, H). In tube formation assays, RBMS1 knockdown substantially inhibited endothelial tubulogenesis, resulting in sparse and fragmented networks with fewer branches. Conversely, RBMS1 overexpression markedly enhanced this process, promoting the formation of extensive, well‐connected tubular structures (Figure 2I, J).

### 2.3 RBMS1 Associates With ATG3 mRNA and Enhances its Stability in VECs

**FIGURE 3:**
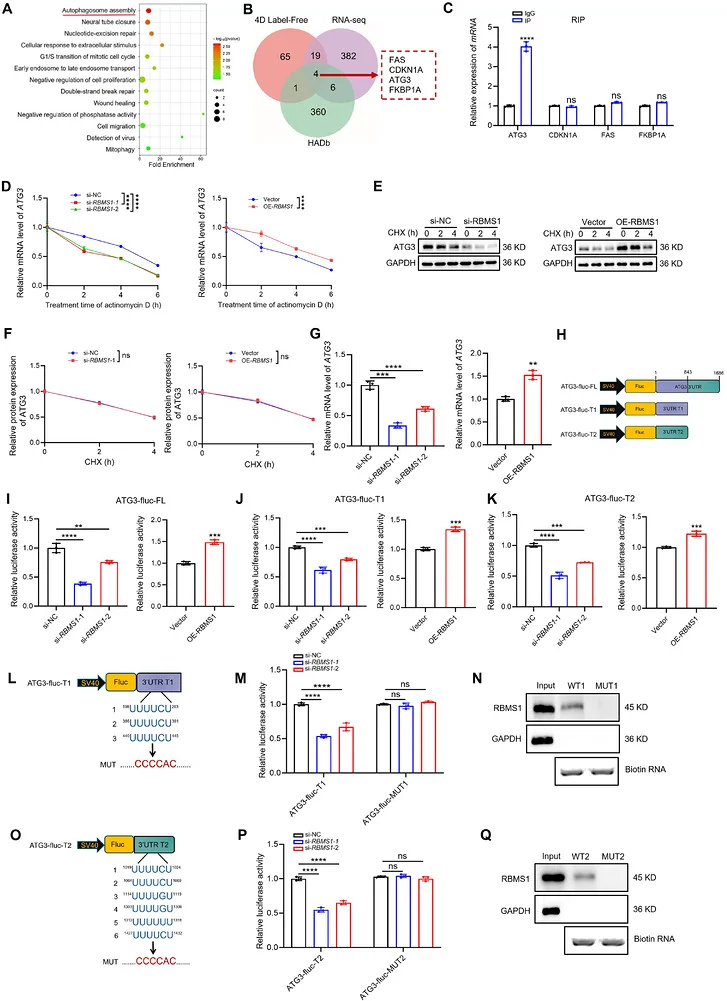
RBMS1 associates with ATG3 mRNA and enhances its stability in VECs. (A) GO enrichment analysis of differentially expressed proteins from 4D label‐free proteomics, sorted by *p*‐value. (B) Venn diagram showing the mRNAs predicted by the 4D label‐free proteomics, RNA‐seq, and the HADb database. (C) RIP assays were conducted with HUVECs using an anti‐RBMS1 antibody or normal IgG. The coprecipitated RNAs of potential targets were quantified by qRT‐PCR (*n* = 3). (D) ATG3 stability was assessed after treatment with actinomycin D for the indicated times (*n* = 3). (E, F) ATG3 protein stability was assessed after treatment with CHX for the indicated times (*n* = 3). (G) Relative mRNA levels of ATG3 in HUVECs were determined by qRT‐PCR after RBMS1 knockdown and overexpression (*n* = 3). (H) Schematic representation of the ATG3 luciferase reporter plasmids. ATG3‐fluc‐FL (the full length of the 3′‐UTR), ATG3‐fluc‐T1 (first−843rd nt region of the 3′‐UTR), and ATG3‐fluc‐T2 (844th–1686th nt region of the 3′‐UTR). (I) The ATG3‐fluc‐FL luciferase reporter plasmid was transiently transfected into HEK293T cells with either RBMS1 knockdown or overexpression. The relative luciferase activity was determined by normalizing the firefly luciferase activity to the Renilla luciferase activity (*n* = 3). (J) The ATG3‐fluc‐T1 luciferase reporter plasmid was transiently transfected into HEK293T cells with either RBMS1 knockdown or overexpression. The relative luciferase activity was determined by normalizing the firefly luciferase activity to the Renilla luciferase activity (*n* = 3). (K) The ATG3‐fluc‐T2 luciferase reporter plasmid was transiently transfected into HEK293T cells with either RBMS1 knockdown or overexpression (*n* = 3). (L) Schematic diagram of the wild‐type ATG3 3'UTR truncation T1 and the mutant MUT luciferase reporter constructs. (M) The ATG3‐fluc‐T1 and ATG3‐fluc‐MUT1 luciferase reporter plasmids were transiently transfected into HEK293T cells under conditions of either RBMS1 knockdown (*n* = 3). (N) RNA pull‐down assay with biotin‐labeled WT1 or MUT1 ATG3 3′‐UTR RNA. The retrieved RBMS1 protein was detected by Western blotting in HUVECs (*n* = 3). (O) Schematic diagram of the wild‐type ATG3 3'‐UTR truncation T2 and the mutant MUT luciferase reporter constructs. (P) The ATG3‐fluc‐T2 and ATG3‐fluc‐MUT2 luciferase reporter plasmids were transiently transfected into HEK293T cells under conditions of either RBMS1 knockdown (*n* = 3). The relative luciferase activity was determined by normalizing the firefly luciferase activity to the Renilla luciferase activity (*n* = 3). (Q) RNA pull‐down assay with biotin‐labeled WT2 or MUT2 ATG3 3′‐UTR RNA. The retrieved RBMS1 protein was detected by Western blotting in HUVECs (*n* = 3). *n* = 3 represents three independent biological replicates obtained from three independently performed experiments. For panels D and F, statistical significance was determined by two‐way ANOVA followed by Šídák′s multiple‐comparisons test; for panels G, I–K, and M, P, statistical significance was determined by one‐way ANOVA followed by Tukey′s multiple‐comparisons test; for panel C, statistical significance was determined by unpaired Student's t‐tests. ^**^ *p* < 0.01, ^***^ *p* < 0.001, and ^****^ *p* < 0.0001. ns, not significant.

We next sought to identify the downstream molecular targets through which RBMS1 exerts these functional effects on VECs, and to this end, we conducted integrated transcriptomic and proteomic profiling of HUVECs after RBMS1 knockdown using RNA Sequencing (RNA‐Seq) and 4D label‐free quantitative proteomics. This multiomics approach systematically captured differentially expressed mRNAs and proteins downstream of RBMS1. Gene Ontology (GO) enrichment analysis of significantly altered proteins revealed a pronounced enrichment in the biological process “autophagosome assembly” (Figure 3A). By intersecting differentially expressed genes from both the RNA‐Seq and proteomic data with entries from the Human Autophagy Database (HADb), we identified four overlapping candidates: FAS, CDKN1A, ATG3, and FKBP1A (Figure 3B). Subsequent RNA immunoprecipitation followed by quantitative PCR (RIP‐qPCR) revealed significant enrichment of ATG3 mRNA in RBMS1 immunoprecipitates compared with the IgG control, supporting an association between RBMS1‐containing ribonucleoprotein complexes and ATG3 mRNA, whereas no significant enrichment was detected for FAS, CDKN1A, or FKBP1A mRNAs under the same conditions (Figure 3C). To further prioritize ATG3, we examined FAS, CDKN1A, and FKBP1A protein levels in vascular endothelial cells from endothelial *Rbms1* conditional knockout mice and littermate controls. Western blotting revealed no significant changes in these proteins following endothelial *Rbms1* deletion (Figure S2). We next performed actinomycin D‐based mRNA stability assays to determine whether RBMS1 posttranscriptionally regulates ATG3. The results showed that RBMS1 knockdown significantly shortened the half‐life and reduced the stability of *ATG3* mRNA, whereas RBMS1 overexpression had the opposite effect (Figure 3D). In contrast, cycloheximide chase experiments revealed that neither RBMS1 knockdown nor overexpression altered the degradation rate of the ATG3 protein (Figure 3E, F), indicating that RBMS1 regulates ATG3 primarily at the mRNA level. Consistent with these findings, RBMS1 knockdown downregulated, whereas its overexpression upregulated, *ATG3* transcription (Figure 3G). Moreover, the protein levels of ATG‐related family members, including ATG5, ATG7, ATG13, and Beclin1, were also assessed under RBMS1 deficiency in both HUVECs and VECs, and no significant changes were detected by Western blotting (Figure S3A, B), ruling out global perturbation of autophagy and reinforcing the specific regulation of RBMS1 on ATG3.

Given that RBPs typically recognize elements in the 3′‐UTR of their target mRNAs [29, 30], we proceeded to map the functional cis‐region within the ATG3 3′‐UTR that underlies RBMS1‐dependent regulation. We generated luciferase reporter constructs containing either full‐length or truncated fragments of the *ATG3* 3′‐UTR (Figure 3H). In HEK293T cells, RBMS1 knockdown significantly suppressed the luciferase activity of the reporter bearing the full‐length *ATG3* 3′‐UTR (*ATG3*‐fluc‐FL), while RBMS1 overexpression increased its activity (Figure 3I). Moreover, the luciferase activities of *ATG3*‐fluc‐T1 and *ATG3*‐fluc‐T2 were significantly reduced or increased, respectively, upon RBMS1 depletion or overexpression, which implied that both truncated 3′‐UTR fragments (T1 and T2) contain RBMS1‐binding sites (Figure 3J, K). Based on previous reports that RBMS1 recognizes conserved motifs in the 3′‐UTR [22, 31, 32], we next searched for potential RBMS1‐binding motifs specifically within the T1 and T2 fragments of the ATG3 3′‐UTR. Notably, this search identified three potential binding sites within the T1 region and six within the T2 region, suggesting that RBMS1 may recognize multiple motifs across the ATG3 3′‐UTR (Figure 3L, O).

To validate these predicted motifs, we mutated them to the sequence CCCCAC within dual‐luciferase reporters and assessed their responsiveness to RBMS1 knockdown in HEK293T cells. The mutant constructs completely lost their response to RBMS1 reduction (Figure 3M, P), confirming that these cis‐elements are essential for RBMS1‐mediated regulation. Having established the functional requirement of these motifs, we asked whether RBMS1 modulates reporter activity through mRNA stability or translational control. Reporter‐mRNA decay assays revealed that RBMS1 knockdown accelerated WT1 and WT2 reporter mRNA decay, whereas the corresponding mutants showed blunted responses (Figure S4A–D), indicating that the ATG3 3’‐UTR elements mediate RBMS1‐dependent mRNA stabilization. Finally, to test whether RBMS1 associates with these RNA elements, we performed RNA pull‐down assays using biotinylated wild‐type or mutant ATG3 3′‐UTR RNAs incubated with cell lysates. Wild‐type RNA efficiently captured RBMS1 protein, whereas the mutant RNA showed markedly reduced binding (Figure 3N, Q). Taken together, these data demonstrate that RBMS1 stabilizes *ATG3* mRNA via direct interaction with its 3′‐UTR, thereby upregulating ATG3 expression without affecting protein turnover.

### 2.4 ATG3 Governs Autophagy and Modulates Key Functional Phenotypes in VECs

**FIGURE 4:**
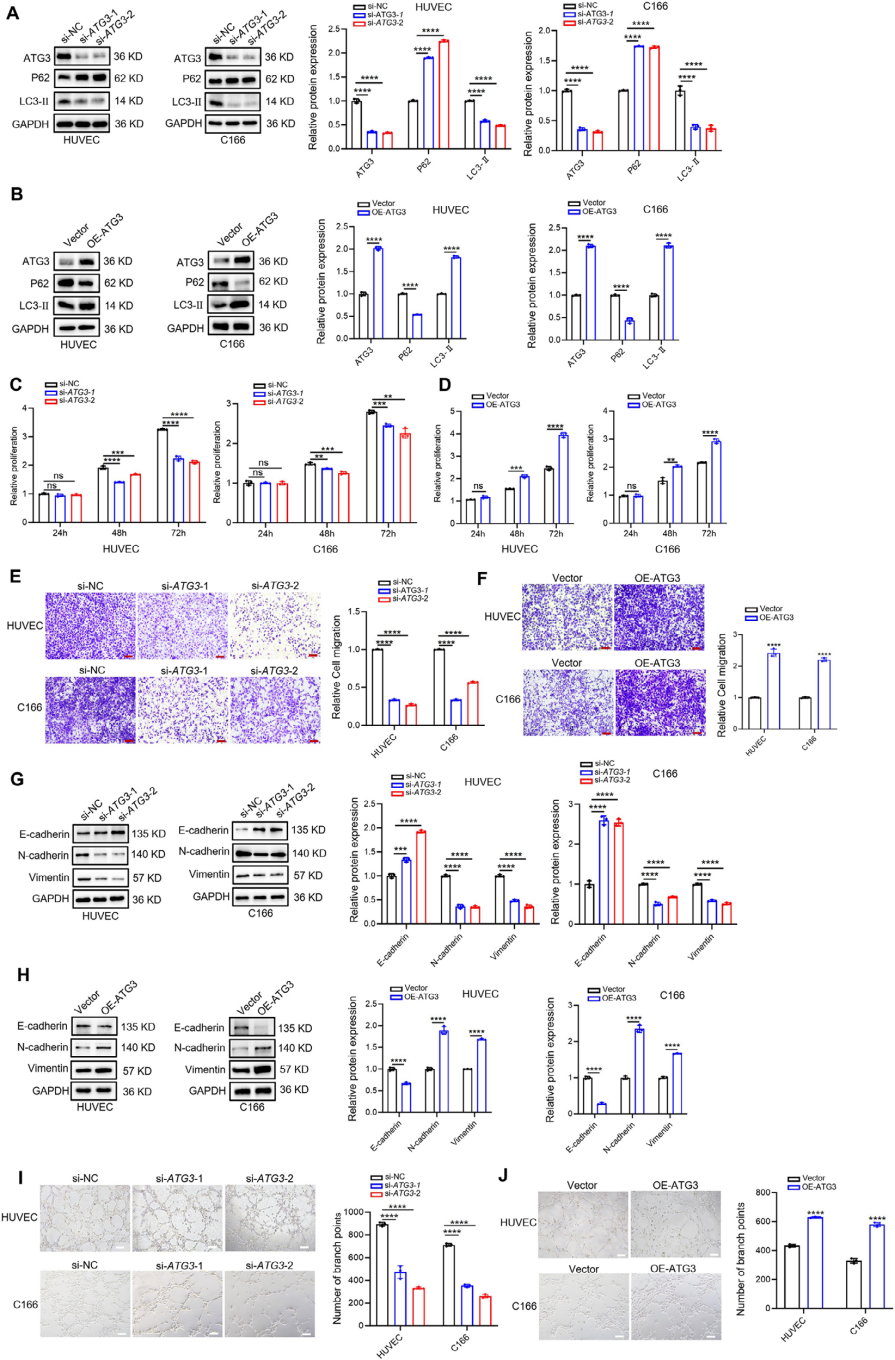
ATG3 governs autophagy and modulates key functional phenotypes in VECs. (A, B) Western blot analysis showing the effects of ATG3 knockdown and overexpression on endothelial function‐related proteins in HUVECs and C166 cells (*n* = 3). (C, D) Cell growth curves were determined by CCK‐8 assays following transfection with si‐ATG3 or OE‐ATG3 (*n* = 3). (E, F) Cell migration capacity was analyzed using a Transwell assay. Representative images of cells that migrated through the porous membrane (crystal violet staining, magnification: × 100, scale bar = 100 µm) (*n* = 3). (G, H) Western blot analysis showing the effects of ATG3 knockdown and overexpression on E‐cadherin, N‐cadherin, and vimentin expression in HUVECs and C166 cells (*n* = 3). (I, J) Representative bright‐field images of tube structures formed by HUVECs and C166 cells transfected with si‐ATG3 or OE‐ATG3 on Matrigel (magnification: × 100, scale bar = 100 µm) and quantitative analysis of the number of branch points per field. *n* = 3 represents three independent biological replicates obtained from three independently performed experiments. Individual data points are shown, and data are presented as the mean± SEM. For panels A, C, E, G, and I, statistical significance was determined by one‐way ANOVA followed by Tukey′s multiple‐comparisons test; for panels B, D, F, H, and J, statistical significance was determined by unpaired Student's t‐tests. ^**^ *p* < 0.01, ^***^ *p* < 0.001, and ^****^ *p* < 0.0001. ns, not significant.

To confirm that ATG3 acts as a molecule that coordinates the autophagy process to maintain vascular endothelial function, we transfected HUVECs and C166 cells with si‐ATG3 (for knockdown) or an overexpression (OE)‐ATG3 plasmid, using si‐NC and an empty vector as controls. After 48 h of transfection, we systematically assessed the functional effects of ATG3 modulation on endothelial proliferation, migration, tube formation, and autophagy‐related protein expression. Western blot analysis revealed that ATG3 knockdown reduced LC3‐II levels while increasing p62 accumulation, whereas ATG3 overexpression increased LC3‐II levels and decreased p62 levels, confirming that ATG3 is an essential regulator of autophagic flux in VECs (Figure 4A, B). Consistent with the functional link between autophagy and cell growth, the CCK‐8 assay results revealed that ATG3 suppression significantly attenuated VEC proliferation, whereas ATG3 overexpression increased proliferation (Figure 4C, D).

Transwell assays further revealed that ATG3 knockdown markedly impaired endothelial cell migration, whereas ATG3 overexpression significantly promoted migration (Figure 4E, F). Similarly, Western blot analysis showed that ATG3 knockdown increased E‐cadherin expression while decreasing N‐cadherin and vimentin expression, whereas ATG3 overexpression produced the opposite pattern (Figure 4G, H). In tube formation assays, ATG3 knockdown substantially inhibited endothelial network assembly, resulting in sparse, fragmented tubules with fewer branches, while ATG3 overexpression robustly enhanced the formation of extensive and interconnected tubular structures (Figure 4I, J). Collectively, these data establish that ATG3 not only governs autophagic activity but also regulates key functional phenotypes, including the proliferation, migration, and angiogenic capacity of VECs.

### 2.5 Endothelial ATG3 Deficiency Exacerbates DVT by Impairing Autophagy In vivo

**FIGURE 5:**
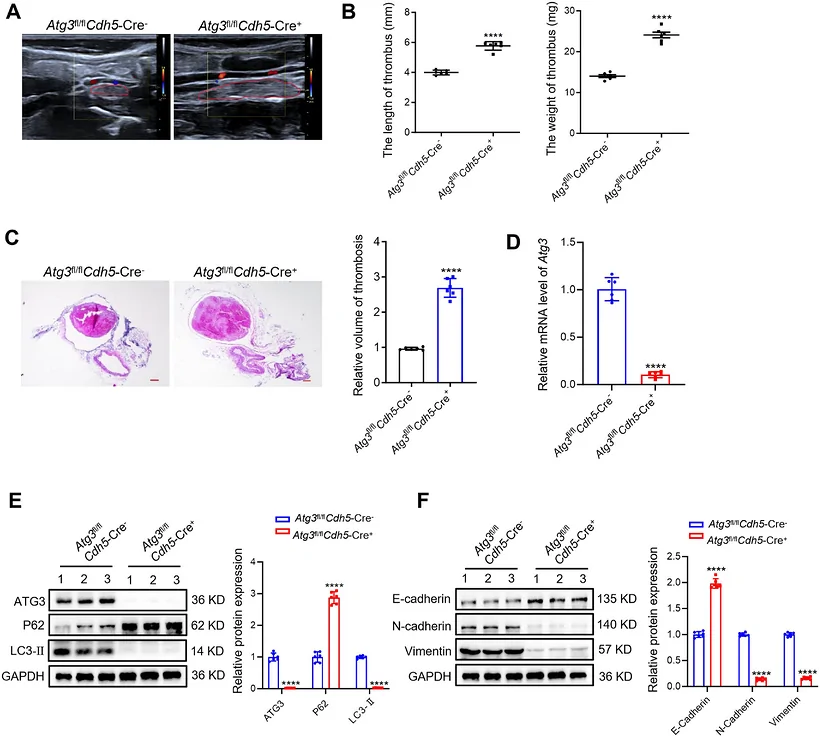
Endothelial ATG3 deficiency exacerbates DVT by impairing autophagy in vivo. (A) Representative vascular ultrasound images of thrombi in *Atg3*‐knockout DVT model mice. (B) Measurement of thrombus weight and length in different groups of mice (*n* = 6). (C) Thrombus size in *Atg3*‐knockout DVT model mice was determined by H&E staining (magnification: × 40, Scale bars = 100 µm). (D) qRT‐PCR analysis of the relative *Atg3* mRNA expression levels in endothelial cell‐conditional *Atg3* knockout mice (*n* = 6). (E) Western blot analysis of autophagy‐related protein expression in endothelial cell‐conditional *Atg3* knockout mice. (*n* = 6). (F) Western blot analysis of E‐cadherin, N‐cadherin, and vimentin expression in vascular endothelial cells isolated from endothelial cell‐conditional Atg3 knockout mice and control littermates (*n* = 6 per group). n represents the number of individual mice. ^****^ *p* < 0.0001 by unpaired Student's t‐test.

To functionally validate the contribution of ATG3‐mediated autophagy to DVT pathogenesis in vivo, we generated endothelial cell‐conditional *Atg3* knockout mice by crossing *Atg3* ^flox/flox^ mice with *Cdh5*‐Cre mice. We also confirmed that *Cdh5*‐Cre did not cause non‐specific deletion of ATG3 by isolating VECs, non‐VECs, and CD45^+^ immune cells from *Atg3* ^fl/fl^ *Cdh5‐*Cre^+^ mice and control littermates. Western blotting showed ATG3 depletion only in VECs (Figure S5A), while its expression remained unchanged in non‐VECs (Figure S5B) and CD45^+^ cells (Figure S5C), confirming endothelial‐specific recombination without off‐target effects. H&E staining combined with murine Doppler ultrasound revealed that the endothelial cell‐conditional *Atg3* deletion significantly exacerbated thrombus formation in a DVT model, demonstrating the aggravating effect of ATG3 loss on venous thrombosis (Figure 5A–C). Consistent with the phenotypic results, molecular analysis confirmed the efficient knockout of *Atg3* in the vascular endothelium of *Atg3* ^fl/fl^ *Cdh5*‐Cre mice, as evidenced by marked reductions in both *Atg3* mRNA and protein levels (Figure 5D, E).

This genetic ablation led to impaired autophagy, characterized by decreased LC3‐II levels and the concomitant accumulation of p62 (Figure 5E). Furthermore, analyses of migration‐related markers revealed that ATG3 knockout increased the expression of E‐cadherin while reducing the expression of N‐cadherin and vimentin (Figure 5F), indicating a shift toward a less migratory and more adhesive endothelial phenotype. In summary, our findings reveal that ATG3 participates in the pathogenesis of DVT by regulating autophagy in VECs.

### 2.6 Impaired Autophagy Contributes to Endothelial Dysfunction in DVT

**FIGURE 6:**
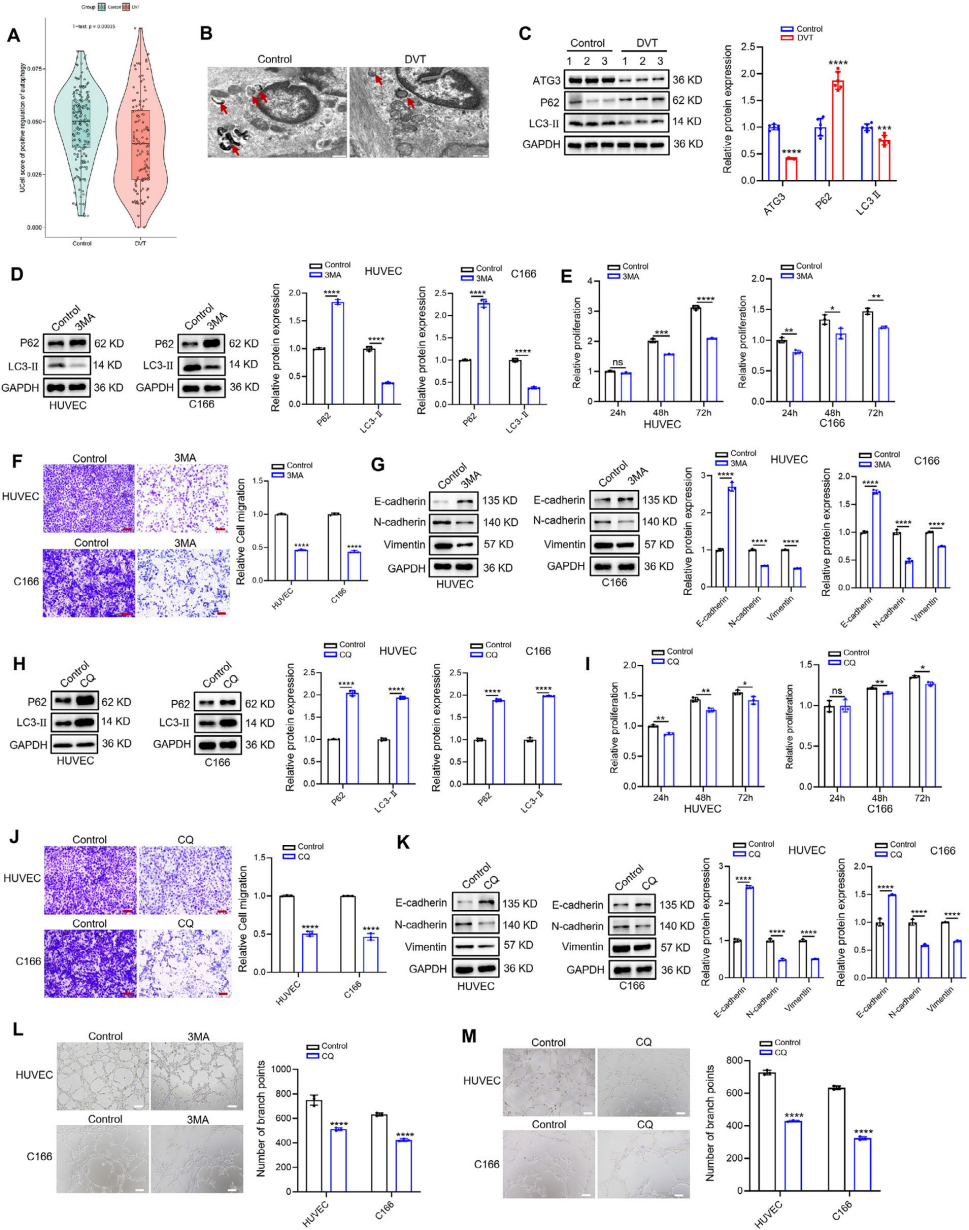
Impaired autophagy contributes to endothelial dysfunction in DVT. (A) Differential autophagic gene set scores in endothelial cells between the DVT and control groups. (B) TEM images showing the ultrastructures of VECs from DVT model mice and control mice. Red arrows indicate typical autophagic structures (autophagosomes or autolysosomes), characterized by double‐membrane structures enclosing undegraded cytoplasmic contents. (magnification: × 10000, Scale bar = 500 nm). (C) Western blot analysis of autophagy‐related protein expression in mouse VECs (*n* = 6). (D, H) Western blot analysis of autophagy‐related proteins (ATG3, LC3‐II, and p62) in HUVECs and C166 cells treated with 3‐MA or CQ (*n* = 3). (E, I) CCK‐8 assay showing cell proliferation (*n* = 3). (F, J) Transwell assay assessing cell migration ability (magnification: × 100, scale bar = 100 µm) (*n* = 3). (G, K) Western blot analysis of E‐cadherin, N‐cadherin, and vimentin expression (*n* = 3). (L, M) Tube formation assay on Matrigel (magnification: × 100, scale bar = 100 µm). The data are presented as the mean± SEM. ^*^ *p* < 0.05, ^**^ *p* < 0.01, ^***^ *p* < 0.001, ^****^ *p* < 0.0001 by unpaired Student's t‐test. ns, not significant.

Since ATG3 is a downstream target of RBMS1 and a key regulator of autophagy, we next examined whether autophagy is impaired in DVT and the consequent impact on endothelial function. First, we analyzed a publicly available single‐cell RNA‐seq dataset of DVT (GSE221978). Following standard processing through the Seurat pipeline, endothelial cell transcriptomes were extracted and subjected to gene set enrichment analysis using the UCell package. Notably, compared with control, endothelial cells from DVT samples exhibited significantly lower enrichment scores for the GO term “positive regulation of autophagy”, indicating the systemic downregulation of autophagic activity in DVT vasculature (Figure 6A). Consistent with the transcriptional signature, transmission electron microscopy (TEM) of vascular endothelial cells from DVT model mice revealed a marked reduction in both the number of autophagosomes and the number of autolysosomes relative to those in control tissues, further corroborating the impairment of autophagic flux (Figure 6B). Western blot analysis of endothelial cells isolated from the model mice confirmed the coordinated suppression of autophagy, characterized by decreased protein levels of ATG3 and the lipidated form of LC3‐II, as well as the accumulation of the autophagy substrate p62 (Figure 6C).

To determine whether autophagy is essential for vascular endothelial cell homeostasis, we pharmacologically inhibited distinct stages of autophagic flux in HUVECs and C166 cells using 3‐methyladenine (3‐MA, an inhibitor of autophagosome formation) and chloroquine (CQ, an inhibitor of autophagosome‒lysosome fusion). Western blot analysis confirmed that both inhibitors effectively disrupted autophagic features; that is, 3‐MA increased p62 accumulation and reduced the lipidated form of LC3‐II (Figure 6D). CQ increased p62 accumulation and the lipidated form of LC3‐II (Figure 6H). Autophagy inhibition significantly impaired VEC proliferation (Figure 6E, I) and migration (Figure 6F, J) and consistently altered the expression of key migration‐related proteins, such as N‐cadherin and vimentin, while upregulating E‐cadherin expression (Figure 6G, K). Furthermore, both 3‐MA and CQ treatment markedly attenuated the tube‐forming capacity of VECs in vitro (Figure 6L, M). These results collectively demonstrate that intact autophagic flux is required for maintaining the functional integrity of VECs.

### 2.7 RBMS1 Promotes Autophagy in VECs via ATG3 Stabilization

**FIGURE 7:**
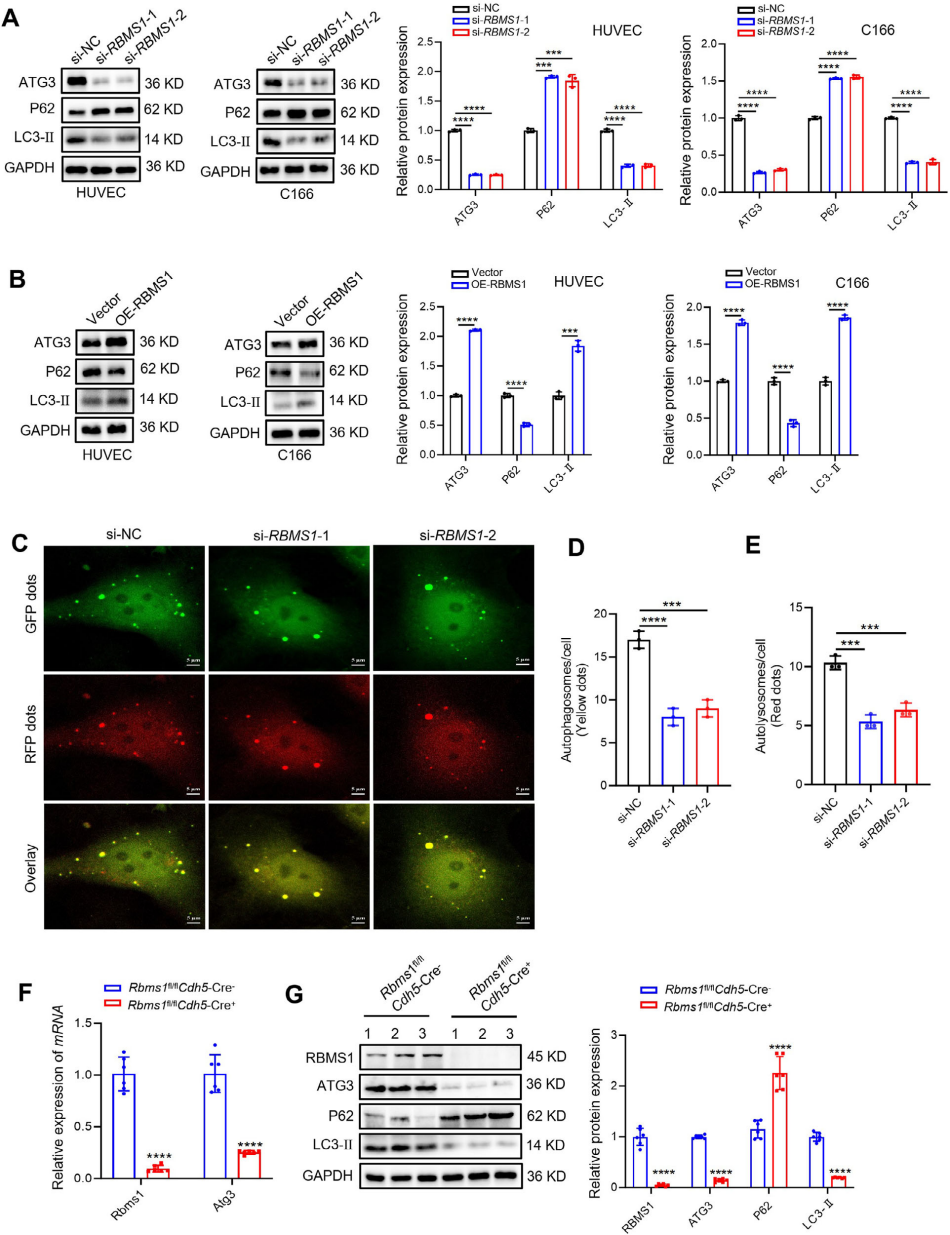
RBMS1 promotes autophagy in VECs via ATG3 stabilization. (A) Western blot analysis of autophagy‐related protein expression following RBMS1 knockdown in HUVECs and C166 cells (*n* = 3). (B) Western blot analysis of autophagy‐related protein expression following RBMS1 overexpression in HUVECs and C166 cells (*n* = 3). (C) In HUVECs transfected with si‐RBMS1, autophagic flux was assessed by confocal microscopy 48 h after infection with mRFP‐GFP‐LC3 adenovirus (magnification: × 400, Scale bars = 5 µm) (*n* = 3). (D, E) Quantification shows the number of (D) autophagosomes (yellow/red puncta, mRFP^+^GFP^+^) and (E) autolysosomes (red‐only puncta, mRFP^+^GFP^−^) per cell in each group. (F) qRT‐PCR analysis of the relative *RBMS1* and *ATG3* mRNA expression levels in endothelial cell‐conditional *Rbms1* knockout mice (*n* = 6). (G) Western blot analysis of autophagy‐related protein expression in endothelial cell‐conditional *Rbms1* knockout mice (*n* = 6). *n* = 3 represents three independent biological replicates obtained from three independently performed experiments. *n* = 6 represents the number of individual mice in each group. For panels A and D, E, statistical significance was determined by one‐way ANOVA followed by Tukey′s multiple‐comparisons test; for panels B and F, G, statistical significance was determined by unpaired Student's t‐tests. ^***^ *p* < 0.001, and ^****^ *p* < 0.0001.

To determine whether RBMS1 modulates autophagic activity in VECs through the regulation of ATG3, we first examined the direct effect of RBMS1 on autophagy. RBMS1 knockdown in both HUVECs and C166 cells significantly reduced LC3‐II levels and increased p62 accumulation, whereas RBMS1 overexpression increased LC3‐II levels and decreased p62 levels. These findings indicate that RBMS1 functions as a positive regulator of autophagy in VECs (Figure 7A, B). To further assess autophagic flux, we performed confocal microscopy analysis of HUVECs infected with an monomeric red fluorescent protein (mRFP)‐green fluorescent protein (GFP)‐LC3 adenoviral reporter. In this system, autophagosomes appear as yellow puncta (GFP^+^RFP^+^), whereas autolysosomes appear as red puncta (RFP^+^ only) because of GFP quenching in acidic compartments. RBMS1 knockdown significantly decreased the number of red puncta, indicating impaired autophagic flux (Figure 7C–E). Consistent with these in vitro observations, endothelial cell‐conditional *Rbms1* knockout in mice (*Rbms1* ^fl/fl^ *Cdh5*‐Cre^+^) led to a marked reduction in both *Rbms1* and *Atg3* expression at the mRNA and protein levels in isolated VECs, accompanied by decreased LC3‐II abundance and increased p62 expression compared with those in the control group (*Rbms1* ^fl/fl^ *Cdh5*‐Cre^−^) (Figure 7F, G).

Having established that RBMS1 knockdown potentially impairs autophagic flux in cultured VECs, we next asked whether this defect also occurs in vivo and whether it reflects reduced flux or late‐stage blockade, by administering CQ to endothelial cell‐conditional *Rbms1* knockout mice. Briefly, endothelial conditional *Rbms1* ^fl/fl^ *Cdh5*‐Cre^+^ mice and control littermates underwent DVT surgery, and 1 h post‐stenosis, CQ (60 mg/kg) or an equivalent volume of vehicle (saline) was delivered intraperitoneally. Subsequently, endothelial cells were isolated, and P62 and LC3‐II protein levels were determined by Western blot. The results showed that CQ treatment induced a marked accumulation of P62 and LC3‐II in control endothelial cells, whereas this response was significantly blunted in *Rbms1* conditional knockout (CKO) endothelial cells (Figure S6A), indicating that RBMS1 deficiency attenuates autophagic flux in vivo rather than blocking lysosomal degradation. In vitro, a CQ‐clamp assay further confirmed that RBMS1 knockdown blunted CQ‐induced LC3‐II accumulation and further elevated p62 levels in HUVECs (Figure S6B). Collectively, these data establish that RBMS1 promotes autophagic flux in VECs and that its loss leads to downregulation of ATG3 expression and consequent autophagy suppression both in vitro and in vivo.

### 2.8 RBMS1‐Dependent ATG3 Sustains Autophagy and Attenuates Thrombosis In vivo and In vitro

For further validation of the functional importance of RBMS1 and ATG3 in DVT pathogenesis, we performed in vivo rescue experiments by administering endothelial‐targeting (ENT serotype) Adeno‐associated virus (AAV) carrying RBMS1 or ATG3 overexpression sequences via tail vein injection into DVT model mice. As the data shown, Doppler ultrasound and HE staining consistently revealed markedly reduced thrombus size in mice overexpressing either RBMS1 (Figure S7A, B) or ATG3 (Figure S7D, E), and measurements of thrombus length and weight further confirmed that both factors significantly alleviated thrombus formation compared with empty‐vector controls (Figure S7C, F), indicating that restoration of either factor attenuates thrombus formation and supporting the functional relevance of the RBMS1 mediated ATG3 regulation in DVT pathology.

**FIGURE 8:**
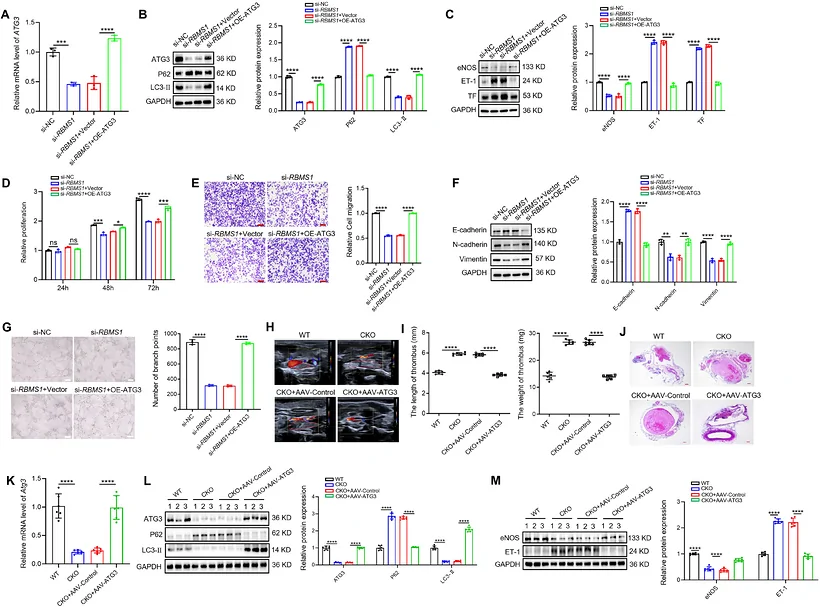
RBMS1‐dependent ATG3 sustains autophagy and attenuates thrombosis in vivo and in vitro. (A) The relative mRNA levels of ATG3 in HUVECs treated as indicated were determined by qRT‐PCR (*n* = 3). (B) Representative Western blot images of ATG3, p62, and LC3‐II in HUVECs under different treatment conditions (*n* = 3). (C) In HUVECs transfected with si‐RBMS1 and subsequently overexpressing ATG3, the expression of endothelial functional proteins was assessed by Western blotting (*n* = 3). (D) Cell growth curves were generated by CCK‐8 assays after si‐RBMS1 transfection followed by ATG3 overexpression (*n* = 3). (E) The migratory ability of HUVECs transfected with si‐RBMS1 and subsequently overexpressing ATG3 was assessed by a Transwell assay (magnification: × 100, Scale bars = 100 µm) (*n* = 3). (F) In HUVECs transfected with si‐RBMS1 and subsequently subjected to ATG3 overexpression, the expression levels of E‐cadherin, N‐cadherin, and vimentin were assessed by Western blotting (*n* = 3). (G) The tube formation ability of HUVECs transfected with si‐RBMS1 and subsequently overexpressing ATG3 was assessed by an in vitro vasculogenesis assay (magnification: × 100, Scale bars = 100 µm) (*n* = 3). (H) Thrombus size in different groups of mice was measured by murine Doppler ultrasound. (I) Quantitative analysis of thrombus length and weight in the indicated groups of mice (*n* = 6). (J) Thrombus size in different groups of mice was assessed by H&E staining (magnification: × 40, Scale bars = 100 µm). (K) Relative *Atg3* mRNA levels were determined by qRT‐PCR in VECs isolated from the following groups: WT, CKO, CKO+ AAV‐Control, and CKO+ AAV‐ATG3 (*n* = 6). (L) Western blot analysis was performed to assess the expression of autophagy‐related proteins in VECs isolated from the indicated groups of mice (*n* = 6). (M) The expression of endothelial function‐related proteins in VECs from different groups of mice was assessed by Western blotting (*n* = 6). *n* = 3 represents three independent biological replicates obtained from three independently performed experiments. *n* = 6 represents the number of individual mice in each group. ^*^ *p* < 0.05, ^**^ *p* < 0.01, ^***^ *p* < 0.001, and ^****^ *p* < 0.0001, as determined by two‐way ANOVA followed by Šídák′s multiple‐comparisons test; ns, not significant.

To establish that RBMS1 promotes autophagy primarily through ATG3, we performed rescue experiments in RBMS1 knockdown HUVECs by overexpressing ATG3. Notably, ATG3 overexpression effectively restored autophagic activity impaired by RBMS1 deficiency, as demonstrated by the recovery of ATG3 and LC3‐II protein levels and the reduction in p62 levels (Figure 8A–C). Consistent with the restoration of autophagic activity, significant functional recovery was also observed in terms of proliferation (Figure 8D), migration (Figure 8E, F), and tube formation ability (Figure 8G) of HUVECs. We therefore generated an AAV (ENT serotype) encoding Atg3 under the control of the TIE promoter (AAV‐TIE‐Atg3) and administered it via tail vein injection into *Rbms1* CKO mice to achieve predominantly endothelial‐directed Atg3 reconstitution, aiming to determine whether ATG3 could alleviate the exacerbated thrombosis phenotype. H&E staining and murine Doppler ultrasound revealed that compared with the control, *Rbms1* deletion markedly increased thrombus size, whereas endothelial‐targeted Atg3 overexpression substantially attenuated this pro‐thrombotic phenotype (Figure 8H–K). At the molecular level, *Rbms1* knockout downregulated both *Atg3* mRNA and protein expression and impaired autophagy, as indicated by decreased LC3‐II levels and increased p62 levels. Importantly, AAV‐mediated ATG3 overexpression in *Rbms1‐*deficient mice restored the expression of autophagy‐related proteins (Figure 8L) and rescued endothelial functional deficits (Figure 8M).

To assess whether the RBMS1‐ATG3 axis regulates a coagulation‐relevant endothelial phenotype, we measured VWF release into the culture supernatants of primary endothelial cells isolated from *Atg3*‐CKO mice and littermate controls, as well as from wild‐type (WT), *Rbms1*‐CKO, *Rbms1*‐CKO+AAV‐Control, and *Rbms1*‐CKO+AAV‐*Atg3* mice. The results showed that endothelial *Atg3* deletion significantly increased VWF release compared with littermate controls (Figure S8A). Similarly, VWF secretion was markedly elevated in endothelial cells from *Rbms1*‐CKO mice relative to WT controls. Administration of AAV‐Control did not reverse this increase, whereas endothelial‐targeted ATG3 re‐expression significantly reduced VWF release to levels comparable to WT controls (Figure S8B). These findings indicate that disruption of ATG3‐dependent autophagy correlates with enhanced endothelial VWF release, and that restoring ATG3 partially reverses this prothrombotic phenotype in *Rbms1*‐deficient mice.

### 2.9 Systemic Association of RBMS1 Downregulation With Impaired Autophagy in PBMCs From Patients With DVT

**FIGURE 9:**
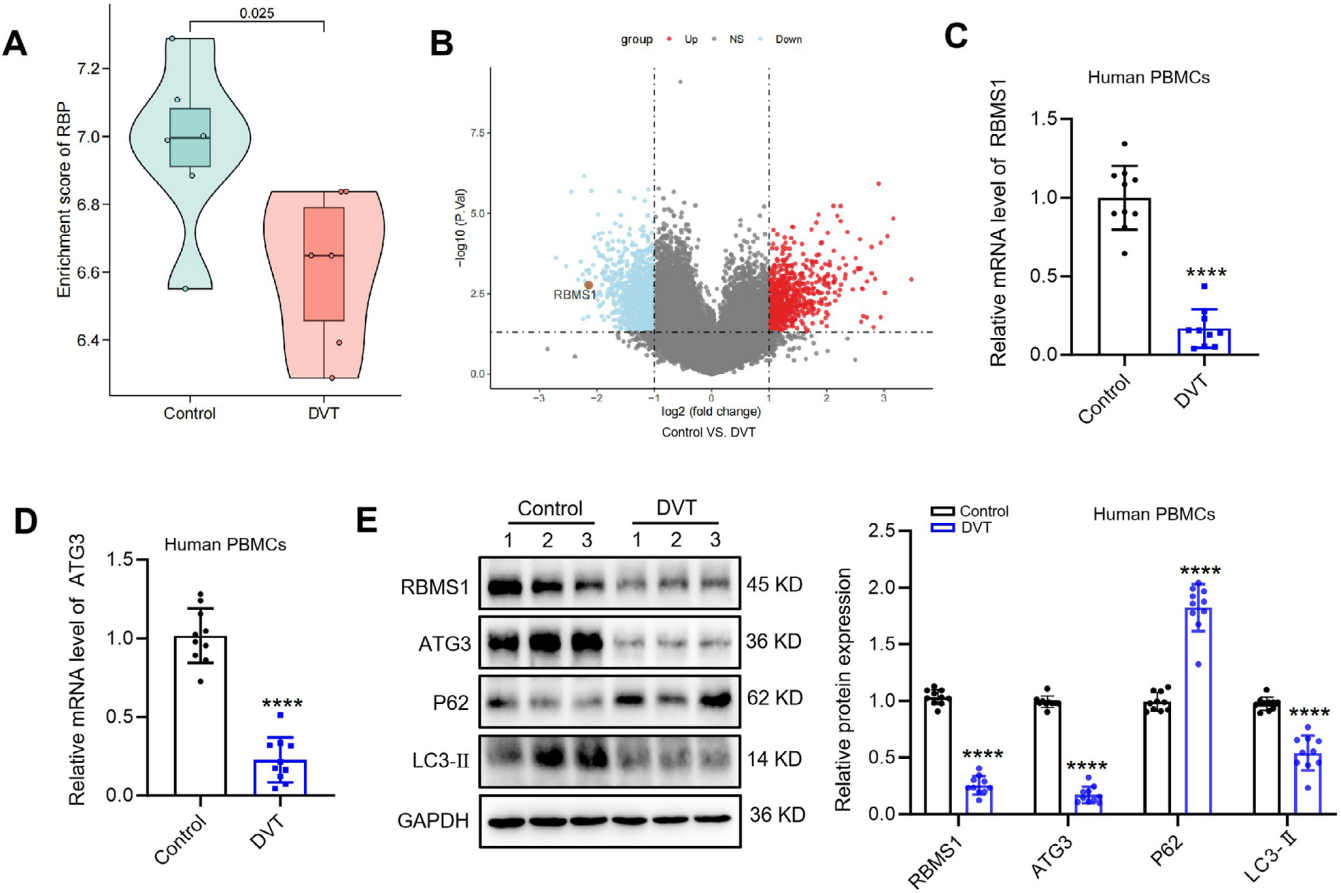
Systemic association of RBMS1 downregulation with impaired autophagy in PBMCs from patients with DVT. (A) Differential enrichment scores for RBP gene sets in PBMCs from the DVT and control groups in the microarray discovery cohort (*n* = 6 per group). (B) Volcano plot of the PBMC microarray analysis in the discovery cohort (*n* = 6 per group). (C) qRT‐PCR analysis of RBMS1 mRNA expression in PBMCs from patients with DVT and healthy controls in the expanded cohort (*n* = 10 per group). (D) qRT‐PCR analysis of *ATG3* mRNA expression in PBMCs from the expanded cohort (*n* = 10 per group). (E) Representative Western blot images and densitometric quantification of RBMS1, ATG3, LC3‐II, and p62 protein levels in PBMCs from patients with DVT and healthy controls (*n* = 10 biological samples per group). ^****^ *p* < 0.0001 by unpaired Student's t‐test for C, D, E.

To clinically validate the association between RBMS1 and deep vein thrombosis (DVT), we collected peripheral blood mononuclear cells (PBMCs) from patients diagnosed with DVT and performed comprehensive whole‐genome microarray profiling. Using the RBPs cataloged in the RBPDB and PROTEOMICSDB databases, we performed single‐sample gene set enrichment analysis (ssGSEA) on the transcriptomic data. The results revealed a significant reduction in the overall RBP enrichment score in DVT patients compared with that in healthy donors, indicating the broad dysregulation of posttranscriptional regulatory networks in DVT (Figure 9A). Volcano plot analysis of differentially expressed genes further revealed RBMS1 as one of the most prominently downregulated RBPs in the DVT cohort (Figure 9B). Consistent with this transcriptional signature, both the mRNA and protein expression levels of RBMS1 were markedly lower in PBMCs isolated from DVT patients than in those from controls (Figure 9C, E). In parallel, a significant decrease in *ATG3* mRNA expression was observed in the same patient‐derived samples (Figure 9D). Western blot analysis corroborated these findings, revealing the coordinated suppression of the autophagy pathway in DVT. Specifically, the expression of LC3‐II and ATG3 was significantly reduced while that of p62 was markedly elevated in the PBMCs of DVT patients (Figure 9E). Collectively, these clinical data suggest that RBMS1 expression is downregulated in DVT and associated with impaired autophagic activity, reinforcing its potential role in DVT pathogenesis. However, its clinical utility requires further validation in larger cohorts.

## 3 Discussion

This study systematically elucidates a novel mechanism in which the RNA‐binding protein RBMS1 inhibits DVT by stabilizing *ATG3* mRNA and promoting autophagy in VECs. As a key posttranscriptional regulator, RBMS1 plays an important role in maintaining vascular endothelial homeostasis. RBMS1 downregulation, coupled with impaired autophagy, constitutes a significant pathological link in the development of DVT. The pathogenesis of DVT involves multiple factors, including venous stasis, endothelial injury, and hypercoagulability, with endothelial dysfunction recognized as an initiating event [33]. While traditional research has focused on the coagulation cascade and platelet activation, the regulatory networks governing intrinsic endothelial protective mechanisms remain insufficiently understood [34, 35]. Single‐cell transcriptomic analysis of the murine DVT model demonstrated reduced *Rbms1* expression in vascular endothelial cells, whereas the analysis of patient‐derived PBMCs revealed a parallel systemic association between DVT and reduced RBMS1 expression. Using an endothelial cell‐conditional knockout mouse model, we confirmed that RBMS1 deficiency markedly exacerbated thrombus formation, indicating the protective function of RBMS1 in the endothelium.

We observed downregulation of RBMS1 expression in DVT patients. Current evidence indicates that RBMS1 is governed by an intricate, multilayered regulatory network. At the transcriptional level, the ETS variant transcription factor 1 directly binds the RBMS1 promoter to activate its expression [36]. Post‐transcriptionally, the m6A reader YTHDC2 associates with RBMS1 mRNA to promote its decay [37]. Concurrently, the oncogenic miR‐106b targets evolutionarily conserved sequences within the RBMS1 3’ UTR, driving translational repression and accelerated mRNA turnover [38]. Additionally, RBMS1 mRNA stability and subcellular compartmentalization are further fine‐tuned by 3′‐UTR cis‐regulatory elements and interacting RNA‐binding proteins in a cell‐type‐ and stage‐dependent manner [39]. However, the precise mechanisms driving RBMS1 dysregulation in DVT remain to be fully elucidated, and systematically investigation of this complex regulatory landscape could yield novel mechanistic insights and therapeutic avenues.

How RBMS1, an RNA‐binding protein, influences endothelial function through posttranscriptional regulation is well explained by this work. Our multiomics analysis revealed the autophagy‐related gene ATG3 as a key downstream target of RBMS1. Further mechanistic investigation revealed that RBMS1 associates with ATG3 mRNA through its 3′‐UTR, upregulating ATG3 expression by enhancing mRNA stability without affecting the protein degradation rate. ATG3, a key enzyme in LC3 lipidation during autophagy, directly influences autophagic flux [40, 41]. We confirmed that the downregulation of ATG3 expression leads to autophagy dysfunction, manifested as reduced LC3‐II expression and p62 accumulation, whereas ATG3 overexpression effectively restores autophagic activity. This discovery links RBP‐mediated posttranscriptional regulation with the crucial cellular degradation pathway of autophagy, revealing a novel mechanism for endothelial protection. Beyond DVT, RBMS1‐mediated ATG3 stabilization may represent a conserved protective module in other vascular diseases with endothelial compromise, such as atherosclerosis, ischemia‐reperfusion injury, and hypertension, offering a unifying framework for RBP‐mediated endothelial stress responses.

The functional integrity of endothelial cells depends on the precise regulation of autophagy. Using both genetic and pharmacological interventions, we demonstrate that autophagy impairment comprehensively compromises endothelial cell proliferation, migration, and tube‐forming capacity. Notably, knockdown of ATG3 recapitulates these functional defects and leads to aberrant expression of E‐cadherin and decreased N‐cadherin and vimentin, suggesting that ATG3 depletion drives an endothelial phenotypic switch toward a pro‐thrombotic state. In animal models, endothelial‐specific *Atg3* knockout significantly exacerbated thrombosis, accompanied by reduced autophagic flux and impaired endothelial integrity, further confirming the protective role of ATG3 in vascular homeostasis. Complementary pharmacological inhibition of autophagy with 3‐MA and CQ produced consistent functional impairments across proliferation, migration, and tube formation, corroborating the ATG3 genetic findings and reinforcing the conclusion that autophagy disruption, irrespective of the intervention, undermines endothelial function. Collectively, these results suggest that the ATG3‐dependent autophagic axis serves as a critical node in maintaining endothelial phenotype, and that fine‐tuned modulation of this pathway may offer a superior strategy for preserving vascular health in thrombosis.

Several limitations of this study should be acknowledged. First, in vivo experiments were conducted using a single murine model of DVT. Although this model is well‐established for studying venous thrombosis, the conclusions regarding RBMS1 function and its therapeutic relevance require validation in alternative models. Second, our rescue experiments employed AAV‐mediated overexpression of ATG3 driven by a strong endothelial‐directed promoter, which may result in abnormally high expression levels. These experiments were intended to establish a mechanistic link, but further studies using pharmacological modulation are needed to assess translational potential. Third, the human data in this study were derived from PBMCs and should therefore be interpreted as evidence of a systemic association rather than direct validation of an endothelial‐specific mechanism. In addition, the clinical analysis was based on a modest sample size of ten participants per group, limiting the generalizability of the findings. Larger studies incorporating human endothelial‐relevant specimens and independent clinical cohorts are needed to determine whether the RBMS1–ATG3 axis operates similarly in human vascular endothelium and to further evaluate the biomarker potential of RBMS1 in DVT.

In conclusion, this study elucidates the protective role of RBMS1 in DVT. Our findings indicate that RBMS1 associates with ATG3 mRNA through its 3′‐UTR and enhances its stability, thereby sustaining autophagic flux in VECs and attenuating thrombus formation. This posttranscriptional regulatory mechanism is essential for maintaining endothelial homeostasis and ultimately attenuating thrombus formation. The finding that RBMS1 modulates ATG3 expression and autophagic activity offers a novel conceptual framework for understanding DVT etiology, highlighting the importance of RNA‐binding protein‐mediated gene regulation in vascular pathophysiology.

## 4 Materials and Methods

### 4.1 Single‐Cell Transcriptomics Analysis

Single‐cell RNA sequencing (scRNA‐seq) data from the mouse inferior vena cava (GSE221978) were processed using the Seurat pipeline in R. After batch correction and integration via single‐cell variational inference (scVI), cell populations were annotated based on canonical marker genes. Differential expression analysis comparing vascular endothelial cells between DVT and control groups was performed, and differentially expressed genes were defined by |log2FC|> 0.5 and Benjamini‐Hochberg adjusted *p* < 0.005. Functional scores for proliferation, migration, and angiogenesis were computed with the UCell package.

### 4.2 Cell Culture and Transfection

HEK293T cells, human umbilical vein endothelial cells (HUVECs), and mouse endothelial cells (C166) (Procell Life Science, Wuhan, China) were maintained in DMEM supplemented with 10% fetal bovine serum (FBS) at 37°C with 5% CO_2_. Cells were transfected with specific siRNAs (GenePharma, Shanghai, China) or overexpression plasmids (MiaolingPlasmid, Wuhan, China) using Lipofectamine 3000 (Invitrogen, USA) in accordance with the manufacturer's instructions (sequences in Table 1). The construct for ATG3 overexpression was generated by inserting the ATG3 coding sequence (without the 3’‐UTR) into the pCDH‐CMV‐MCS‐EF1‐GFP vector, and no exogenous tag was fused.

**TABLE 1:** List of oligo sequences used in this study.

Si‐RNA oligos	Sequences (5'‐3')
Si‐NC	UUCUCCGAACGUGUCACGUTT
	ACGUGACACGUUCGGAGAATT
Si‐*RBMS1*‐1 (Homo sapiens)	GAUGCAACCUCAACCAUAUTT
	AUAUGGUUGAGGUUGCAUCTT
Si‐*RBMS1*‐2 (Homo sapiens)	GACGUCUAAUGACCAUUCUTT
	AGAAUGGUCAUUAGACGUCTT
Si‐*Rbms1*‐1 (Mus musculus)	CUCCUACAGAACCUUUACUTT
	AGUAAAGGUUCUGUAGGAGTT
Si‐*Rbms1*‐2 (Mus musculus)	CAGUACGCCACCUAUUACUTT
	AGUAAUAGGUGGCGUACUGTT
Si‐*ATG3*‐1 (Homo sapiens)	CCAGAACUUAUGACCUUUATT
	UAAAGGUCAUAAGUUCUGGTT
Si‐*ATG3*‐2 (Homo sapiens)	GCUGCAGAUAUGGAAGAAUTT
	AUUCUUCCAUAUCUGCAGCTT
Si‐*ATG3*‐1 (Mus musculus)	GAUGGAGUAUUCGGAUGAATT
	UUCAUCCGAAUACUCCAUCTT
Si‐*ATG3*‐2 (Mus musculus)	CAGCUGGAGAUCACUUAGUTT
	ACUAAGUGAUCUCCAGCUGTT

### 4.3 RNA Sequencing and Proteomics Analysis

Total RNA was extracted from HUVECs transfected with control or RBMS1‐targeting siRNA (*n* = 3/group) using TRIzol reagent (CWBIO, China). Transcriptome libraries were prepared with a VAHTS Universal V6 kit and sequenced on an Illumina platform by OE Biotech. For proteomics, proteins from similarly treated cells were subjected to 4D label‐free quantitative analysis by Jingjie post‐translational modification BioLabs (Hangzhou, China).

For omics analyses, biological replicate matrices were analyzed using standardized R‐based workflows. RNA‐seq and microarray data were normalized before differential expression testing, and Benjamini‐Hochberg false discovery rate (FDR) correction was applied to account for multiple comparisons. Differentially expressed transcripts were prioritized using |log2 fold change|> 1 and adjusted *p* < 0.05 unless otherwise stated. For 4D label‐free proteomics, only proteins quantified in at least two of three biological replicates per group were retained; differential proteins were screened using fold change> 1.5 and *p* < 0.05, and FDR‐adjusted values were retained for transparency. GO enrichment analyses were performed using the detected genes or proteins as the background, and overlapping candidates were defined by matched gene symbols across the RNA‐seq, proteomic, and autophagy‐database lists.

### 4.4 Quantitative Real‐Time PCR (qRT‒PCR)

Total RNA was extracted with TRIzol Reagent (CWBIO, Beijing, China) and reverse‐transcribed into cDNA. qRT‐PCR was carried out using SYBR Green (CWBIO, Beijing, China) and a QuantStudio 1 Plus instrument. The reaction was performed in triplicate, and target gene expression was normalized to GAPDH expression. Relative quantification was determined using the 2^−ΔΔCt^ method. The primer sequences are provided in Table 2.

**TABLE 2:** List of primer sequences used in this study.

RT‐qPCR Primers	Sequences (5'‐3')
*RBMS1* (Homo sapiens)	F: AGGTGCAAAGTCCTTCGTGG
	R: GTGACATGGTGTGCTCCATTG
*Rbms1* (Mus musculus)	F: CTACCGCAAGCAGCAGTCTC
	R: CAGCTGATCCCATCCTGAGT
*ATG3* (Homo sapiens)	F: AAACTGATGCTGGCGGTGAA
	R: GCTGCCGTTGCTCATCATAG
*Atg3* (Mus musculus)	F: GGGCTACAGGGGAAGAATTGA
	R: ACACCGCTTGTAGCATGGAA
*GAPDH* (Homo sapiens)	F: GCACCGTCAAGGCTGAGAAC
	R: TGGTGAAGACGCCAGTGGA
*Gapdh* (Mus musculus)	F: CTTTGTCAAGCTCATTTCCTGG
	R: TCTTGCTCAGTGTCCTTGC
*CDKN1A* (Homo sapiens)	F: TGTCTTGTACCCTTGTGCCT
	R: TGGTAGAAATCTGTCATGCTGGTC
*FAS* (Homo sapiens)	F: ATCACCACTATTGCTGGAGTCA
	R: TGTTCTGCTGTGTCTTGGACAT
*FKBP1A* (Homo sapiens)	F: GAAGCTCTGGAGGAAACCCTG
	R: CTCAGCTTCCTCTCCTTTGGG
*Hnrnpa2b1* (Mus musculus)	F: GTTGAGCCAAAACGTGCTGTA
	R: TTTCCAGACTGCCTATCGGTA
*Fus* (Mus musculus)	F: TCAAACGACTATACCCAACAAGC
	R: TGGCCGTATCCTGAAGTGTCA
*Srrm2* (Mus musculus)	F: TTTTGATCCTCAGAGACGTGC
	R: TCGAACAAGGCTGTATGGTTTG
*Rbms1* ^flox/flox^	F: AAGGAGAAAACCCCTATCTTTTGC
	R: AGACAGCAGCTCTTTATGAAACAC
*Atg3* ^flox/flox^	F: GATGTTCGTTGCCTTGCCCT
	R: ATTGGTCTATCTGCTGACTCTGTG
*Cdh5‐Cre* ^+^	F: CCCAGGCTGACCAAGCTGAG
	R: GTGAAACAGCATTGCTGTCACTT

### 4.5 Western Blotting

Proteins were extracted using RIPA lysis buffer and quantified with a bicinchoninic acid assay. Following separation via sodium dodecyl sulfate‐polyacrylamide gel electrophoresis (SDS‐PAGE), the proteins were transferred onto nitrocellulose membranes. The membranes were then blocked with skim milk and incubated overnight at 4°C with a primary antibody. After being washed three times with PBST, the membranes were incubated with an horseradish peroxidase‐conjugated secondary antibody. Protein bands were visualized using a chemiluminescence detection system. The primary antibodies used in this study included anti‐RBMS1 (ab150353), anti‐ATG3 (ab108251), anti‐GAPDH (ab181603), anti‐tissue factor (TF; ab318995), anti‐eNOS (ab76198), Anti‐CDKN1A (p21) (ab109199), and anti‐endothelin (ET)‐1 (ab117757), all sourced from Abcam (Cambridge, UK/USA), as well as anti‐p62 (39749S), anti‐LC3I/II (12741S), anti‐E‐cadherin (3195S), anti‐N‐cadherin (13116S), anti‐vimentin (5741S), anti‐Fas (96825T), and anti‐FKBP1A (94838T) which were obtained from Cell Signaling Technology (MA, USA).

### 4.6 RNA Immunoprecipitation (RIP)‐qPCR

The RNA immunoprecipitation (RIP) assays were performed using a commercial RNA‐Binding Protein Immunoprecipitation Kit (Geneseed Biotech, Guangzhou, China) in accordance with the manufacturer's instructions. Briefly, cell lysates were incubated with magnetic beads conjugated either with an anti‐RBMS1 antibody or with a normal IgG control. Following immunoprecipitation, the co‐precipitated RNAs were extracted and purified, and their enrichment of specific target mRNAs was quantified by qRT‐PCR.

### 4.7 RNA Pull‐Down

Biotin‐labeled RNA probes were transcribed in vitro using the HyperScribe T7 High‐Yield Biotin‐16 RNA Labeling Kit Plus (K1405, APExBIO, USA), with wild‐type or mutant ATG3 3′‐UTR DNA sequences driven by a T7 promoter as templates. After synthesis, probes were purified using RNA purification columns.

For RNA pull‐down, 50 pmol of biotinylated RNA probe was bound to 25 µL of streptavidin agarose in RNA‐binding Buffer for 30 min at room temperature. HUVECs were lysed on ice for 10 min using the kit‐provided lysis buffer, and cleared lysates were obtained by centrifugation at 13 000 g for 10 min at 4°C. The RNA‐agarose complex was then mixed with cell lysates and rotated at 4°C for 2 h. After three washes with wash buffer to remove non‐specific binding, bound proteins were eluted in SDS‐PAGE loading buffer at 100°C for 10 min and analyzed by Western blotting using an anti‐RBMS1 antibody.

### 4.8 RNA Stability Assay

HUVECs were treated with the transcriptional inhibitor actinomycin D (5 µg/mL; AbMole, USA). Total RNA was extracted at designated time points (0, 2, 4, and 6 h) after treatment. The remaining levels of target mRNAs were quantified by qRT‐PCR, and their half‐lives were subsequently calculated.

### 4.9 Protein Stability Assay

Protein stability was assessed by treating HUVECs with the protein synthesis inhibitor cycloheximide (CHX; 100 µg/mL; HY‐12320; MedChemExpress, USA). Following treatment, the cells were harvested at designated time points (0, 2, and 4 h). The remaining levels of the target proteins were analyzed by Western blotting, and their half‐lives were calculated on the basis of band intensity quantification.

### 4.10 Dual‐Luciferase Reporter Assay

The 3'‐UTR of ATG3 was cloned into the pGL3‐3M‐Luc reporter vector. The constructed plasmid was then cotransfected into HEK293T cells together with si‐NC, si‐RBMS1, vector, or OE‐RBMS1 plasmids. After 48 h of transfection, luciferase activity was measured using the Dual‐Luciferase Reporter Assay System (E1910; Promega, Madison, USA) and a GloMax 20/20 Luminometer.

### 4.11 mRFP‐GFP‐LC3 Autophagic Flux Assay

To investigate the role of RBMS1 in autophagy, HUVECs were transiently transfected with RBMS1‐specific siRNA to achieve gene knockdown. The cells were subsequently infected with mRFP‐GFP‐LC3 adenovirus for 48 h to visualize autophagic compartments. After fixation, images were acquired using a confocal microscope (LSM880 NLO; ZEISS, Germany). The number of yellow puncta (indicating autophagosomes) and red puncta (indicating autolysosomes) per cell was quantified with image analysis software. Autophagic flux was assessed by calculating the ratio of red to yellow puncta.

### 4.12 Cell Proliferation Assay

HUVECs and C166 cells were seeded in 96‐well plates at a density of 4000 cells per well and transfected with RBMS1‐targeting siRNAs. Cell viability was subsequently assessed at 0, 24, 48, and 72 h posttransfection using CCK‐8 assays (C0038; Beyotime, Shanghai, China). Specifically, CCK‐8 reagent was added to each well, followed by incubation for 30 min, after which the absorbance at 450 nm was measured with a spectrophotometric microplate reader.

### 4.13 Cell Migration Assay

Cell migration was assessed using Transwell chambers (8‐µm pore size; 3422; Corning, Shanghai, China). HUVECs and C166 cells were suspended in serum‐free medium, and 3000 cells were inoculated into the upper chamber, while medium containing 10% FBS was added to the lower chamber as a chemoattractant. After 24 h of incubation, nonmigrated cells on the upper surface were removed, and migrated cells on the lower surface were fixed, stained with crystal violet, and counted.

### 4.14 Tube Formation Assay

Matrigel (0827045; ABW; Shanghai, China) was polymerized in 96‐well plates at 37°C for 30 min. HUVECs were then seeded onto the Matrigel surface. After 4–8 h of incubation, tube formation was visualized under a microscope. Capillary‐like structures were quantified by measuring the junction points using ImageJ software with the Angiogenesis Analyzer plugin.

### 4.15 Construction and Generation of Endothelial Cell‐Conditional *Rbms1* Knockout Mice

Endothelial cell‐conditional *Rbms1* knockout mice were purchased from Cyagen Biosciences (Suzhou, China). Endothelial cell‐conditional *Rbms1* knockout mice (C57BL/6J, *Rbms1* ^fl/fl^ *Cdh5*‐Cre^+^) mice were generated by crossing *Rbms1* ^flox/flox^ mice with *Cdh5*‐Cre^+^ mice. The genotypes of *Rbms1* knockout mice were confirmed by PCR. The efficiency of *Rbms1* knockout was validated by qRT‐PCR and Western blotting. The primers used for the *Rbms1* and *Cdh5*‐Cre^+^ transgenes are listed in Table 2.

### 4.16 Construction and Generation of Endothelial Cell‐Conditional *Atg3* Knockout Mice

Endothelial cell‐conditional *Atg3* knockout mice were purchased from Cyagen Biosciences (Suzhou, China). Endothelial cell‐conditional *Atg3* knockout (C57BL/6J, *Atg3* ^fl/fl^ *Cdh5*‐Cre^+^) mice were generated by crossing *Atg3* ^flox/flox^ mice with *Cdh5*‐Cre^+^ mice. The genotypes of *Atg3* knockout mice were confirmed by PCR. The efficiency of *Atg3* knockout was validated by qRT‐PCR and Western blotting. The primers used for the *Atg3* and *Cdh5*‐Cre^+^ transgenes are listed in Table 2.

### 4.17 DVT Mouse Model and Treatment

C57BL/6J wild‐type mice (eight weeks old) were purchased from Beijing HFK Bioscience Company (Beijing, China). A mouse model of DVT was established using the inferior vena cava (IVC) stenosis method [42, 43]. All animal experiments were approved by the Institutional Animal Care and Use Committee of Shandong University of Traditional Chinese Medicine (Approval No: SDUTCM20240905001). All animal experiments were randomized, and the outcome assessments were blinded to genotype and treatment allocation. Group sizes were determined based on our preliminary inferior vena cava (IVC) stenosis experiments [44, 45]; the sample size was based on a preliminary independent experiment in which thrombus length at 48 h after DVT induction was defined as the primary outcome. In this pilot study, a clear difference in thrombus length was observed between groups, with relatively low variability within each group. Assuming equal variances, these preliminary data were used to estimate variability and guide sample size calculation. Sample size was determined using a two‐sided unpaired Student's t‐test (α = 0.05, equal allocation). Based on these calculations and to ensure adequate statistical power while maintaining ethical use of animals, a total of six mice per group were included in the final study.

For in vivo rescue experiments, we used two complementary strategies. First, to assess whether RBMS1 or ATG3 overexpression alone is sufficient to attenuate DVT, we administered endothelial‐targeting (ENT serotype) AAVs encoding RBMS1 or ATG3 (AAV‐RBMS1 or AAV‐ATG3; GeneChem, Shanghai, China), which preferentially transduce vascular endothelial cells following systemic administration, or the corresponding empty‐vector control (AAV‐Control), to wild‐type C57BL/6J mice via tail‐vein injection (100 µL, titer 5.13 × 10^1^ ^2^ v.g./mL). Two weeks after AAV delivery, the DVT model was established as described above, and thrombi were harvested 48 h later for H&E staining and Doppler ultrasound imaging.

Second, to determine whether ATG3 reconstitution in the endothelium rescues the pro‐thrombotic phenotype caused by RBMS1 loss, we injected AAV‐ATG3 or AAV‐Control into endothelial cell‐conditional *Rbms1* knockout mice and their control littermates, using the same dose and timing. Mice were divided into 4 groups: (1) DVT group (WT: *Rbms1* ^fl/fl^ *Cdh5*‐Cre^−^ mice), (2) DVT Rbms1 knockout group (CKO: *Rbms1* ^fl/fl^ *Cdh5*‐Cre^+^ mice), (3) DVT *Rbms1* knockout+ Adeno‐associated virus (AAV) control group (CKO+ AAV‐Control mice), and (4) DVT *Rbms1* knockout+ AAV‐ATG3 group (CKO+ AAV‐Atg3 mice). One hundred microliters (5.13 × 10^1^ ^2^ v.g./mL) of AAV‐control or AAV‐ATG3 was injected into *Rbms1* knockout mice via the tail vein.

### 4.18 Transmission Electron Microscopy (TEM)

Autophagic structures within vascular tissues were evaluated at the ultrastructural level using TEM. The microscope was operated at an accelerating voltage of 100 kV to optimize contrast and minimize beam damage to the tissue samples. Images were captured using a Hitachi HT‐7800 digital camera system.

### 4.19 Isolation of Vascular Endothelial Cells, Non‐Endothelial Cells, and CD45^+^ Immune Cells

Vascular endothelial cells (VECs) and non‐endothelial cells (non‐VECs) were isolated from the inferior vena cava of *Rbms1* ^fl/fl^Cdh5‐Cre^+^ mice, *Atg3* ^fl/fl^Cdh5‐Cre^+^ mice, and their respective littermate controls (*Rbms1* ^fl/fl^Cdh5‐Cre^−^ mice and *Atg3* ^fl/fl^Cdh5‐Cre^−^). VECs were isolated using a Mouse Vascular Endothelial Cell Isolation Kit (VE2011M; TBD Sciences, Tianjin, China) according to the manufacturer's instructions. Briefly, the inferior vena cava was dissected, the adventitia and adipose tissue were removed, and the lumen was rinsed with phosphate‐buffered saline. The tissue was minced into approximately 1 mm^3^ pieces, placed on a 70 µm cell strainer, and gently ground with tissue homogenization rinse solution to collect a single‐cell suspension, which was then centrifuged at 400 g for 10 min. The pellet was resuspended in tissue sample dilution buffer. In a 15 mL centrifuge tube, 2 mL of separation solution 1 and 1 mL of separation solution 2 were sequentially added to form a density gradient. The cell suspension was carefully layered on top and centrifuged at 450 g for 30 min. Cells from the second layer were collected as VECs, washed twice with washing buffer and rinsing buffer (400 g for 10 min, followed by 250 g for 10 min). The remaining cell fractions were collected as non‐VECs.

For immune cell isolation, peripheral blood was collected from the same mice. Peripheral blood mononuclear cells (PBMCs) were isolated by density gradient centrifugation using Mouse Mononuclear Cell Separation Medium (LDS1090; TBD Sciences, Tianjin, China). CD45^+^ immune cells were then positively selected using CD45 MicroBeads (Miltenyi Biotec, mouse CD45 MicroBeads, 130‐052‐301) following the manufacturer's protocol. All isolated cell populations were immediately subjected to protein extraction and Western blot analysis.

### 4.20 In Vivo and In Vitro Autophagic Flux Assays

To assess autophagic flux in vivo, endothelial cell‐conditional *Rbms1* knockout mice and their littermate controls were subjected to DVT surgery as described above. 1 h after surgery, CQ (60 mg/kg body weight; HY‐17589A, MedChemExpress, USA) or an equal volume of sterile saline (vehicle control) was administered via intraperitoneal injection, as previously described [46]. At 48 h post‐injection, vascular endothelial cells were isolated from the inferior vena cava, and p62 and LC3‐II protein levels were subsequently examined by Western blot.

For the in vitro autophagic flux assay, HUVECs were transfected with RBMS1‐targeting siRNA (si‐*RBMS1*) or negative control siRNA (si‐NC). At 24 h after transfection, the cells were treated with CQ (20 µm) or an equal volume of vehicle for an additional 48 h. The cells were subsequently harvested, and the protein levels of p62 and LC3‐II were determined by Western blotting. All experiments were performed in three independent biological replicates.

### 4.21 Hematoxylin and Eosin (H&E) Staining

The inferior vena cava vascular tissue of mice was fixed in 4% paraformaldehyde, embedded in paraffin, and sectioned at a thickness of 4 µm. Following deparaffinization and rehydration, the sections were stained with H&E in accordance with standard protocols. The stained sections were dehydrated, cleared, and mounted with neutral balsam. Histological morphology was examined under a light microscope (E100; Nikon, Japan).

### 4.22 Murine Doppler Ultrasound Imaging

Mice were anesthetized using isoflurane inhalation. The thoracic or abdominal region was gently shaved, and a depilatory cream was applied to remove residual hair to ensure optimal acoustic coupling. Thrombosis in the murine inferior vena cava was assessed using a small‐animal ultrasound imaging system (VINNO6 LAB; VINNO, Suzhou, China).

### 4.23 Clinical Sample Analysis

Peripheral blood mononuclear cells (PBMCs) were isolated from patients with deep vein thrombosis (DVT, *n* = 6) and healthy controls (*n* = 6). For the discovery‐stage analysis, genome‐wide mRNA expression profiling was performed using PBMCs from six patients with DVT and six healthy controls (*n* = 6 per group) with a human mRNA microarray (4 × 180K; Agilent Technologies) by OE Biotech Co., Ltd. (Shanghai, China), following standard protocols. For targeted expression analysis, an independent validation cohort comprising ten patients with DVT and ten healthy controls was analyzed. This study was approved by the Ethics Committee of the Affiliated Hospital of Shandong University of Traditional Chinese Medicine (Approval No: (2019) Lun Shen No. (008)‐KY), with written informed consent obtained from all participants. Clinical samples were analyzed as independent biological samples. The PBMCs microarray and validation experiments were designed as exploratory clinical validation of the mechanistic findings rather than as a powered diagnostic biomarker study.

### 4.24 ELISA Measurement of Endothelial von Willebrand Factor (VWF)

Endothelial cells were purified from isolated cell suspensions using anti‐mouse CD31 MicroBeads (Miltenyi Biotec, 130‐097‐418) according to the manufacturer's instructions. The purified cells were cultured in endothelial growth medium‐2 supplemented with endothelial growth factors, 10% FBS, and 1% penicillin–streptomycin on fibronectin‐coated plates. Culture supernatants were collected, and VWF levels were measured using a mouse VWF ELISA kit (Keyybio, KYY‐0422M1) according to the manufacturer's protocol. Each biological replicate consisted of endothelial cells isolated from an individual mouse.

### 4.25 Statistical Analysis

All the data are presented as the mean± standard error of the mean (SEM). The sample size (n) represents the number of independent biological replicates, individual animals, or human participants, as specified in the corresponding figure legends. Comparisons between two independent groups were performed using an unpaired two‐sided Student's t‐test. Comparisons among three or more groups involving one independent variable were performed using one‐way analysis of variance (ANOVA), followed by Tukey′s multiple‐comparisons test. Experiments involving two independent variables were analyzed using two‐way ANOVA, followed by Šídák′s multiple‐comparisons test with multiplicity adjustment, as appropriate. All statistical tests were two‐sided, and an alpha level of 0.05 was used. A *p*‐value of less than 0.05 was considered statistically significant. Statistical analyses were performed using GraphPad Prism version 10.1.

## Author Contributions

C.C., Z. Z., B. W., and X. L. conceived and designed the study and acquired funding. C.C. performed the molecular biology experiments and drafted the original manuscript. X. L. conducted the bioinformatics analysis. N. F. and Y. Z. carried out the animal studies. X. Y. and Q. H. performed the in vitro functional assays. W.L. and Y.C. conducted the gene expression assays. M. Z., F. L., and F. S. were responsible for clinical sample collection. All the authors reviewed, edited, and approved the final version of the manuscript.

## Funding

This study was funded by the National Natural Science Foundation of China (82575078, 81673981, 82274575), the Major Basic Research Project of Shandong Natural Science Foundation (ZR2023ZD56), the Science & Technology Cooperation Program of Shandong (2025KJHZ035), the Co‐construction project of the State Administration of TCM (GZY‐KJS‐SD‐2023‐034 and GZY‐KJS‐SD‐2023‐046), the Shandong Province Taishan Scholar Project (No. tstp20240513), the National Youth Qihuang Scholar Training Program, Clinical Research Special Project of Shandong University of Traditional Chinese Medicine (LCKY202427), the Shandong Provincial Traditional Chinese Medicine Science and Technology Project (M20241908, M20241719), and Shandong University of Traditional Chinese Medicine Bianque Scholar Climbing Program‐Li Xia, the Undergraduate Education Reform Project of Shandong Province (Z2024231).

## Ethics Statement

All animal experiments were approved by the Institutional Animal Care and Use Committee of Shandong University of Traditional Chinese Medicine (Approval Number: SDUTCM20240905001). Ethics approval for clinical sample research was granted by the Ethics Committee of the Affiliated Hospital of Shandong University of Traditional Chinese Medicine (Approval Number: (2019) Lun Shen No. (008)‐KY). We obtained written informed consent from all the subjects.

## Conflicts of Interest

The authors declare no conflicts of interest.

## Supporting information




**Supporting File1**: advs77393‐sup‐0001‐SuppMat.docx.

**Supporting File2**: advs77393‐sup‐0002‐SuppMat.pdf.

## Data Availability

The data that support the findings of this study are openly available in NCBI at https://www.ncbi.nlm.nih.gov/geo/query/acc.cgi?acc=GSE307102, reference number GSE307102.
