# Supplementary material for: An RNA‐Binding Protein RBMS1 Promotes Endothelial Cell Autophagy to Maintain Vascular Homeostasis and Suppress Deep Vein Thrombosis
Source: Adv Sci (Weinh). 2026 Aug 27;13(63):e77393. doi: 10.1002/advs.77393 (PMC13521309; doi:10.1002/advs.77393)

Original data of the corresponding images

Original data of the corresponding images in Figure 1

Figure.1H

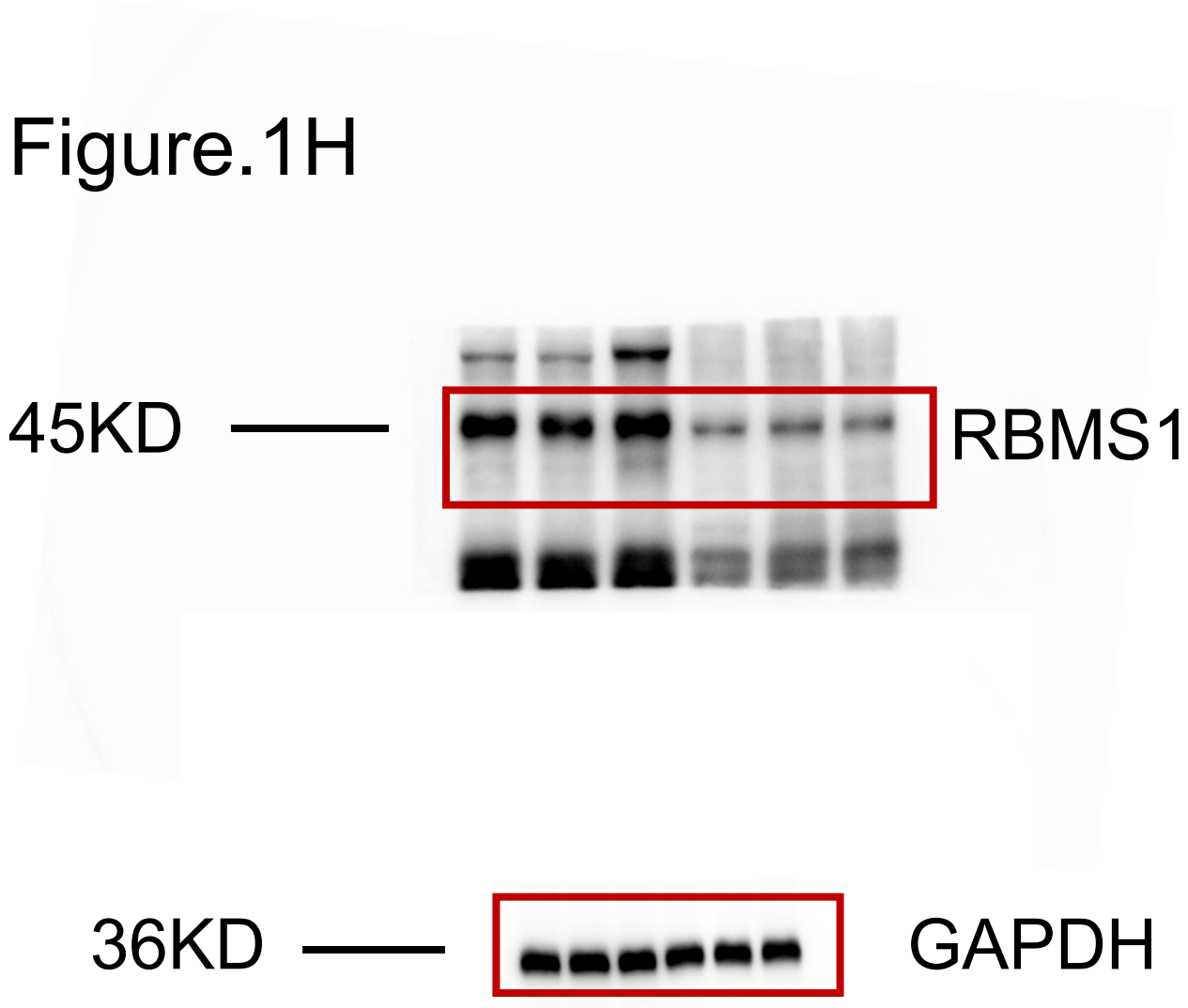

Figure.1I

Rbms1$^{fl/fl}$*Cdh5*-Cre^{-} *Rbms1*$^{fl/fl}$*Cdh5*-Cre^{+}

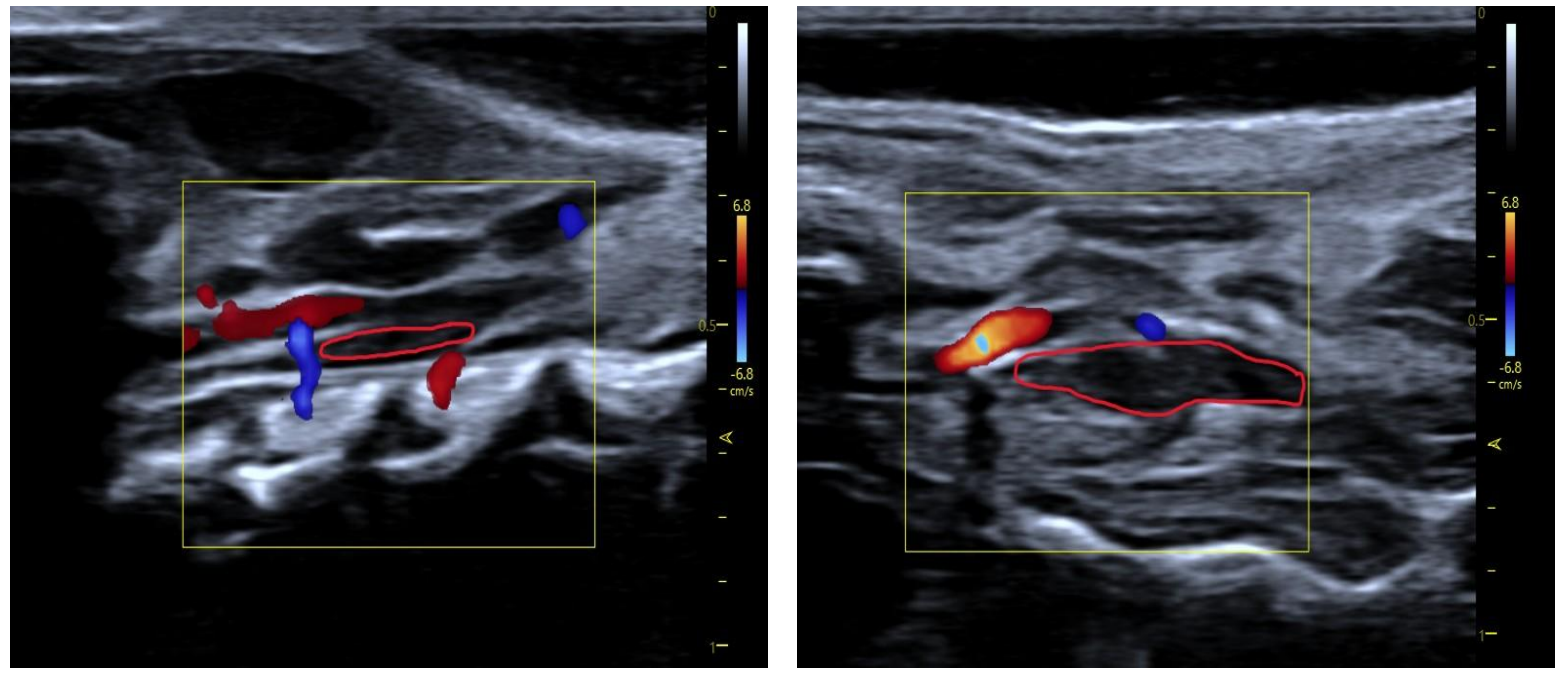

Figure.1K

Rbms1$^{fl/fl}$*Cdh5*-Cre^{-} *Rbms1*$^{fl/fl}$*Cdh5*-Cre^{+}

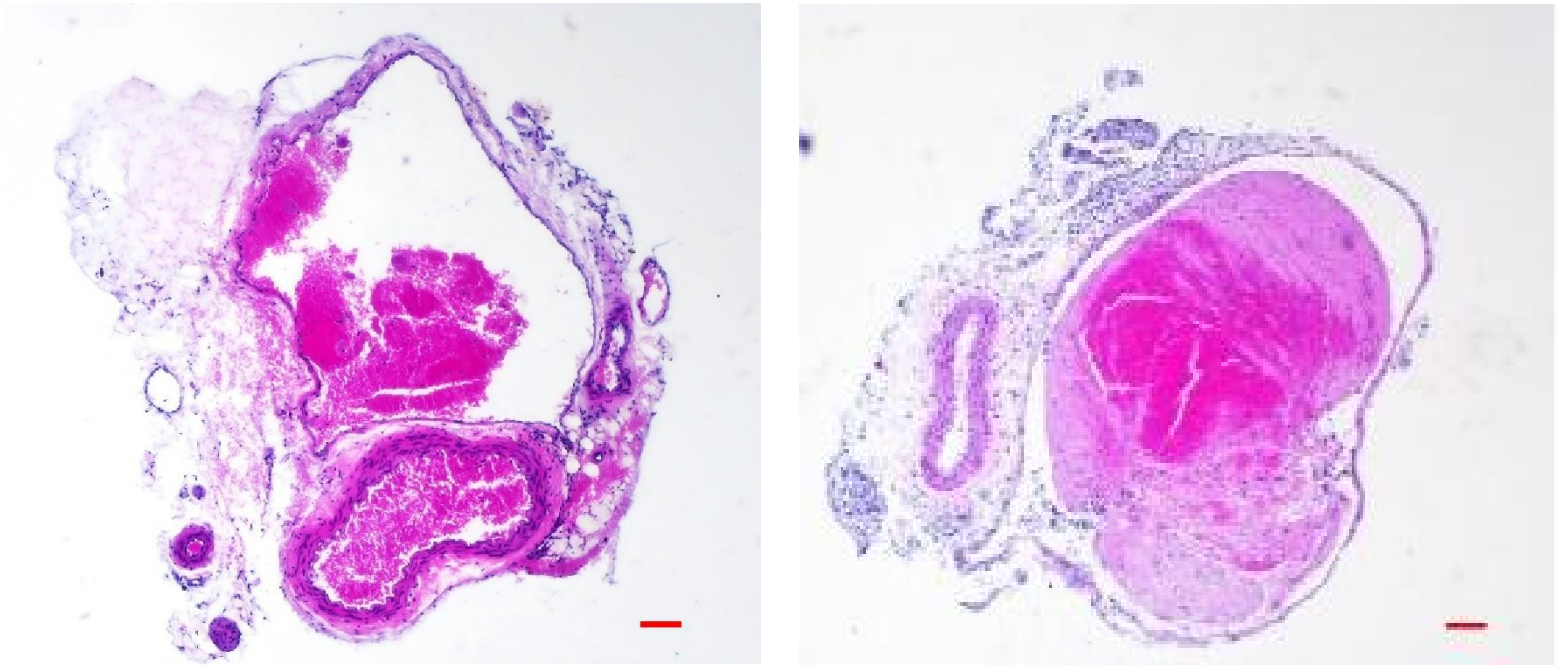

Original data of the corresponding images in Figure 2

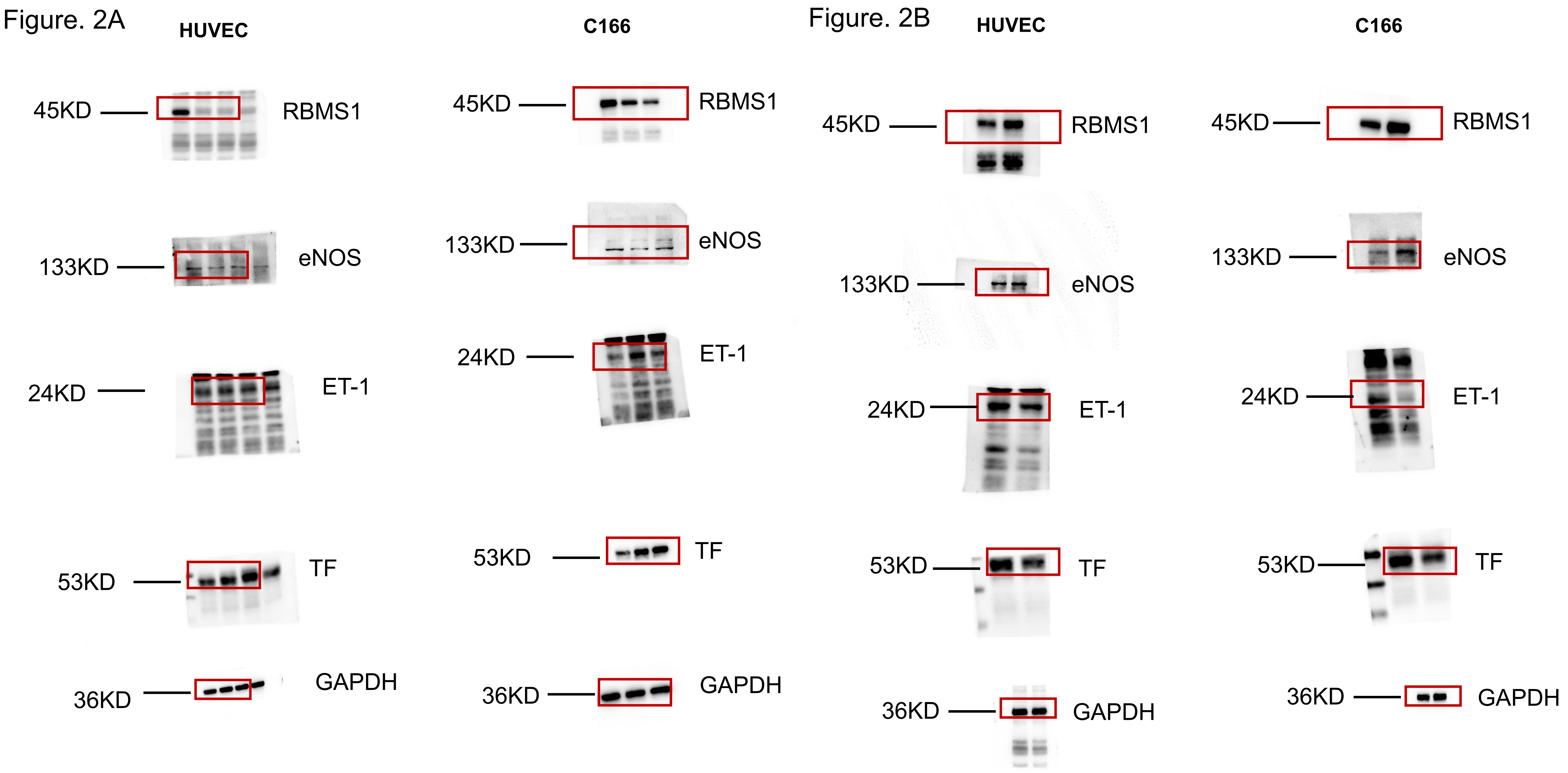

Figure. 2G

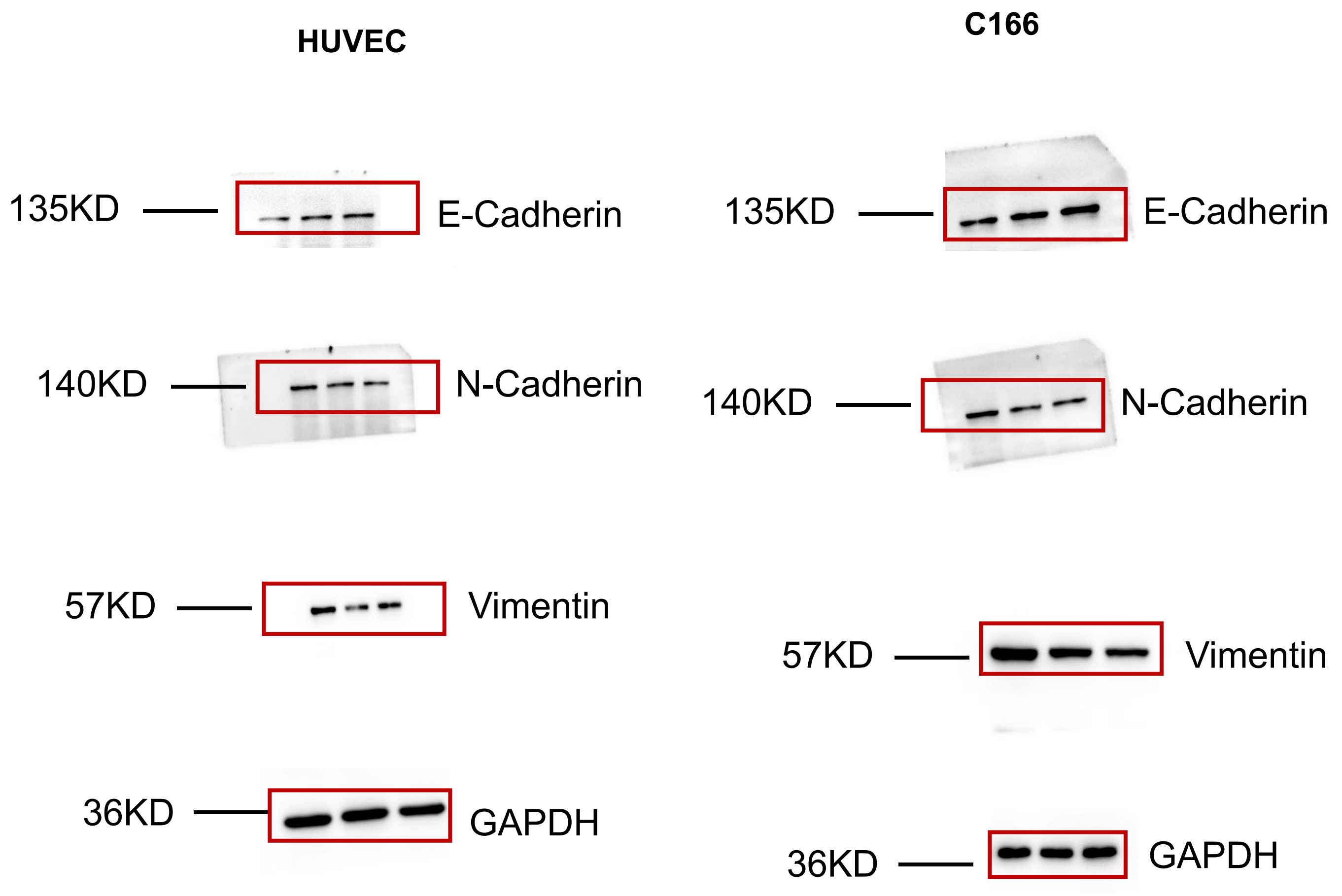

HUVEC
C166
135KD
E-Cadherin
135KD
E-Cadherin
140KD
N-Cadherin
140KD
N-Cadherin
57KD
Vimentin
57KD
Vimentin
36KD
GAPDH
36KD
GAPDH

Figure. 2H

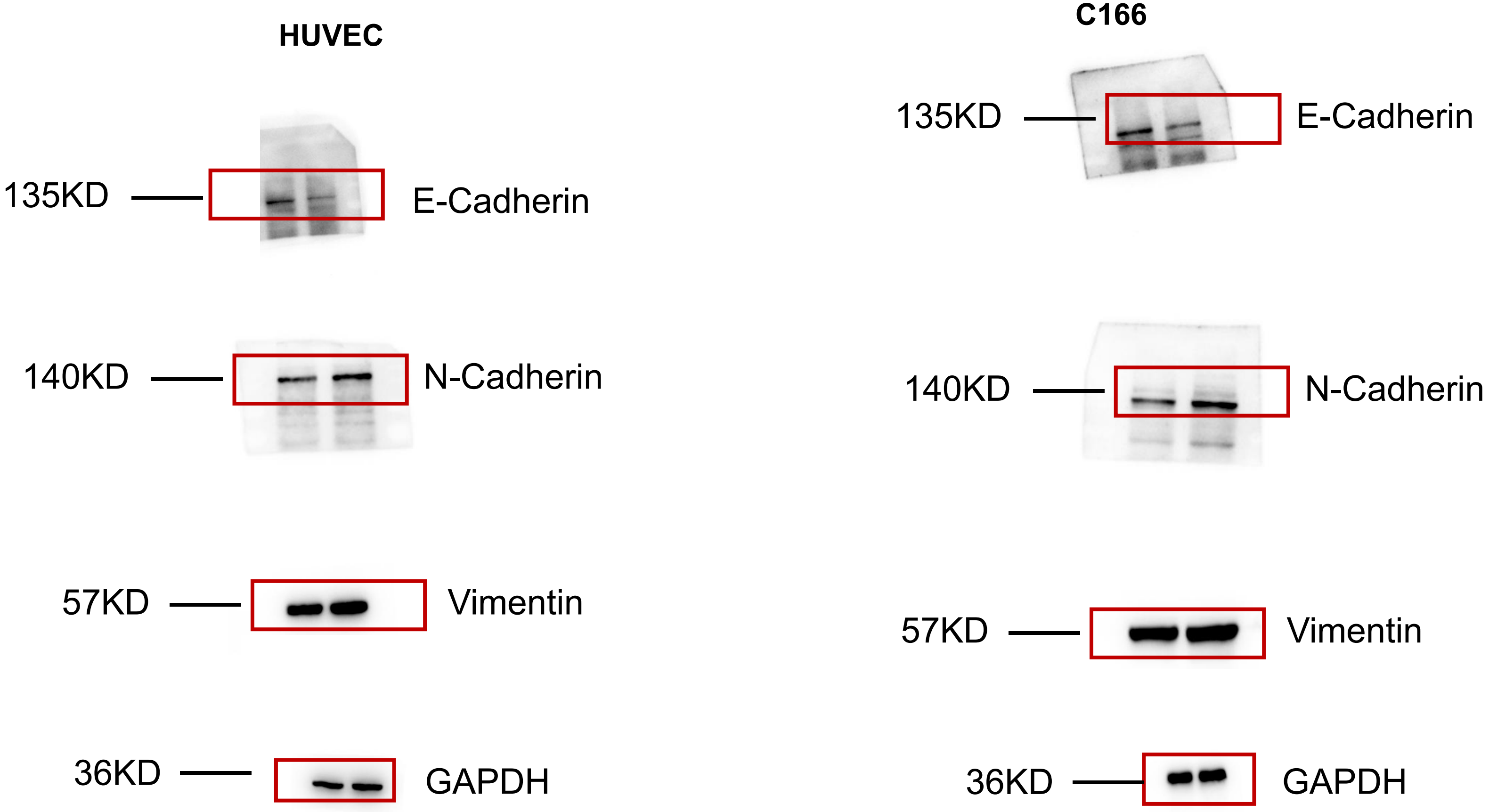
HUVEC
135KD
E-Cadherin
140KD
N-Cadherin
57KD
Vimentin
36KD
GAPDH
C166
135KD
E-Cadherin
140KD
N-Cadherin
57KD
Vimentin
36KD
GAPDH

Figure. 2E

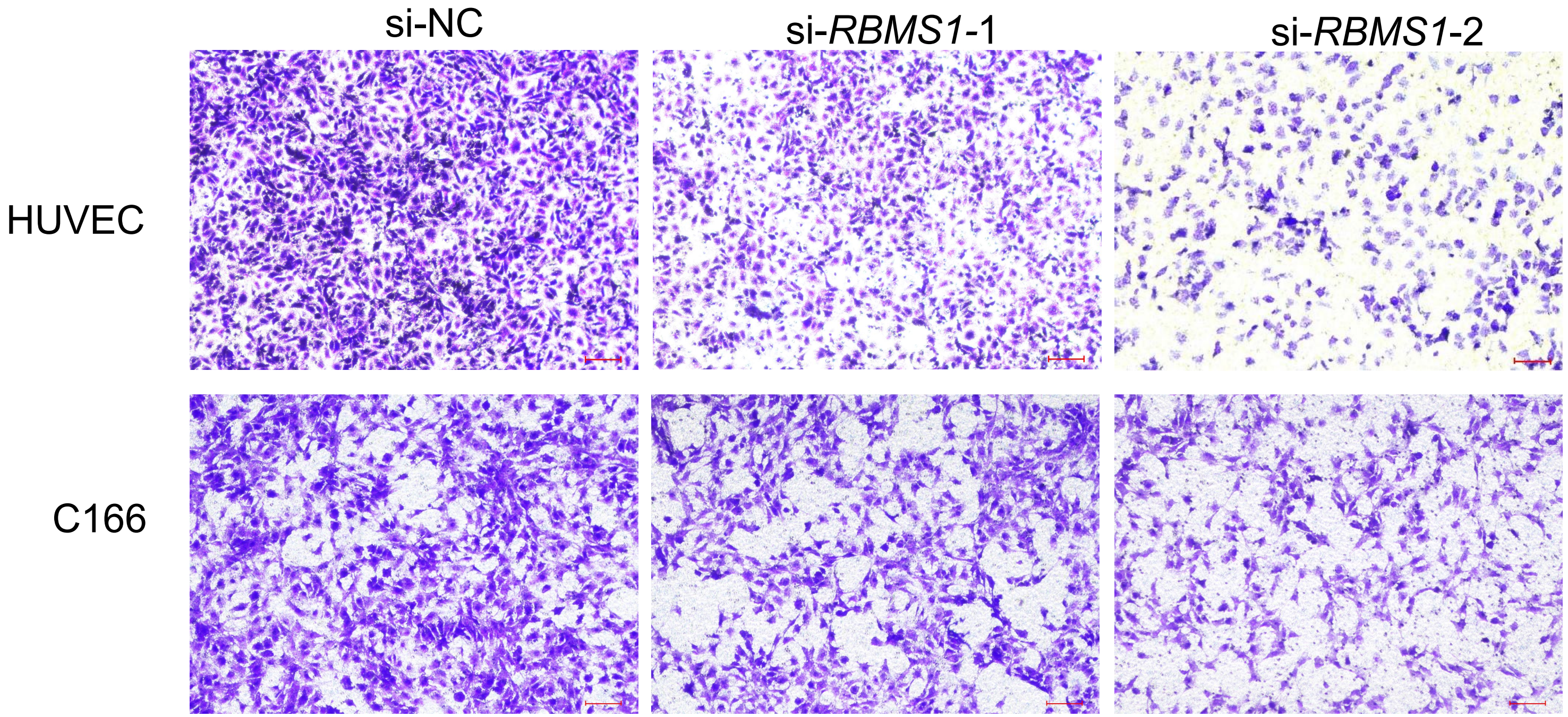

Figure. 2F

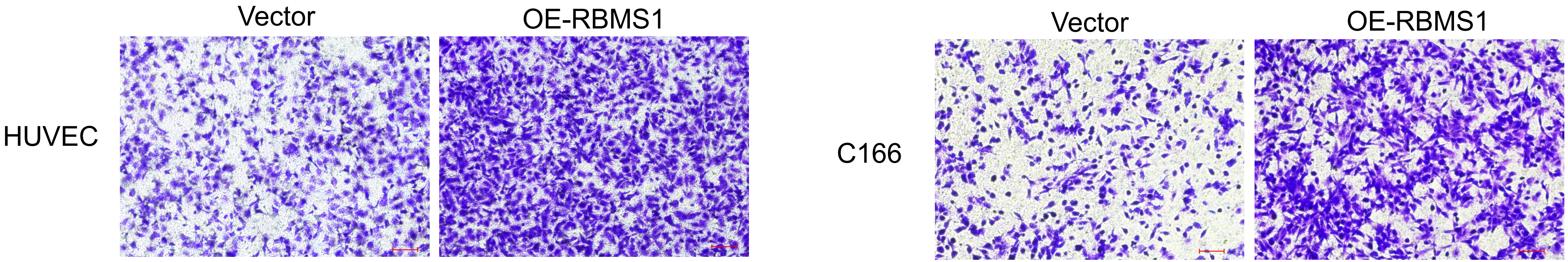

Figure. 2I

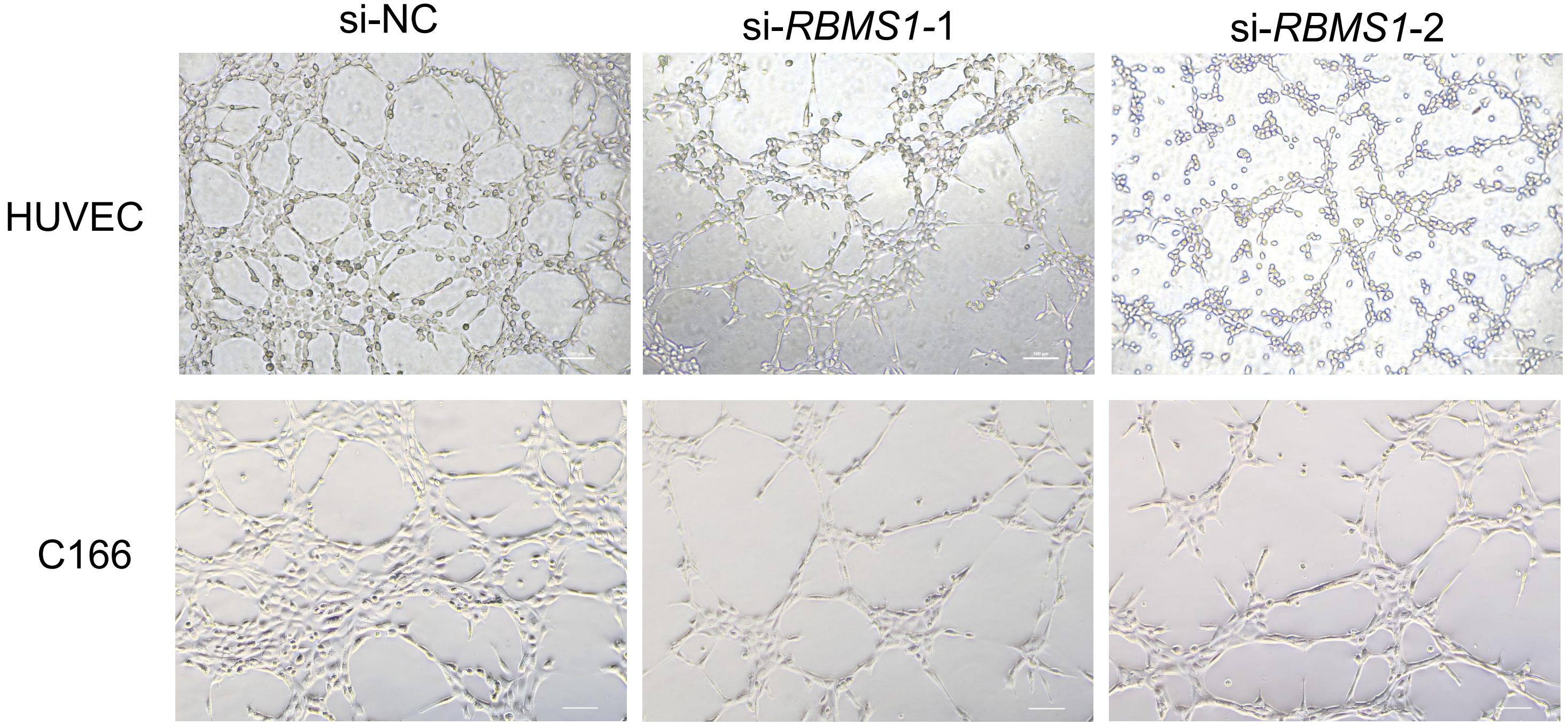

Figure. 2J

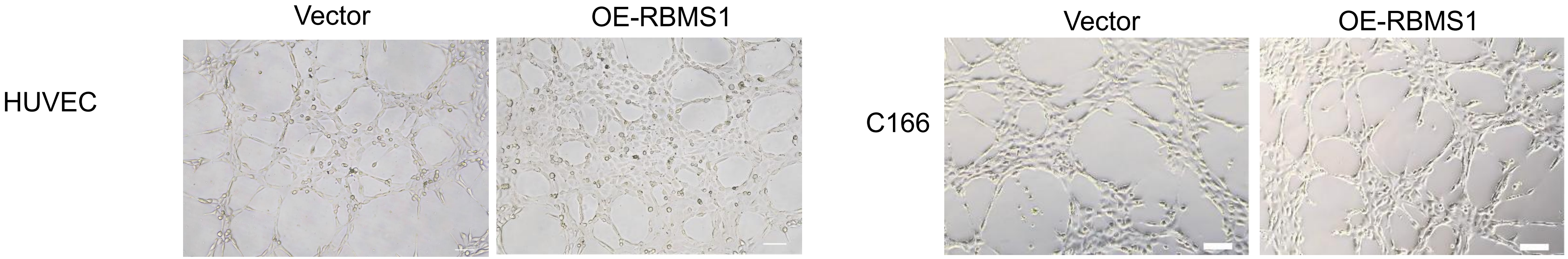

Original data of the corresponding images in Figure 3

Figure. 3E

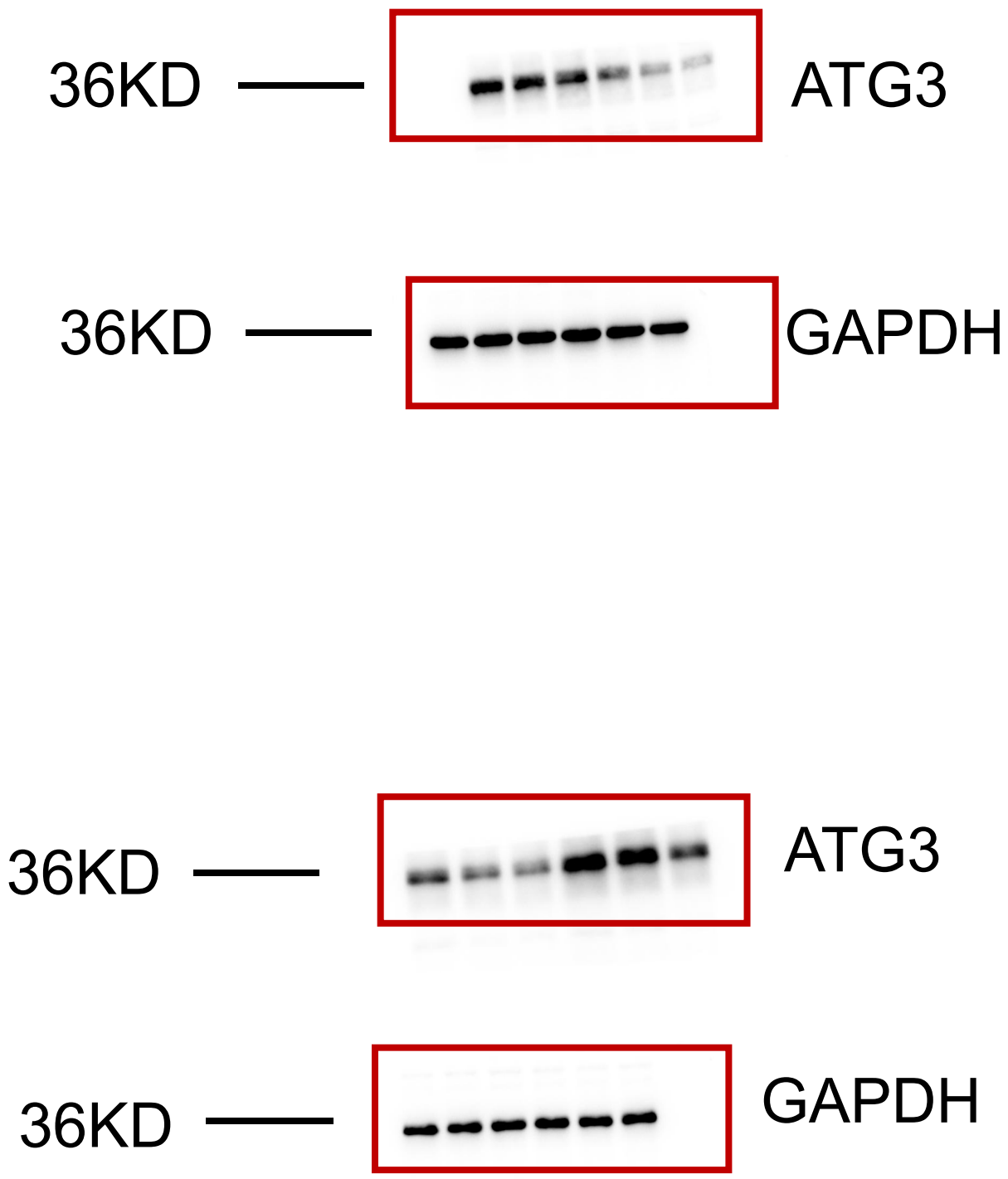

Figure. 3N

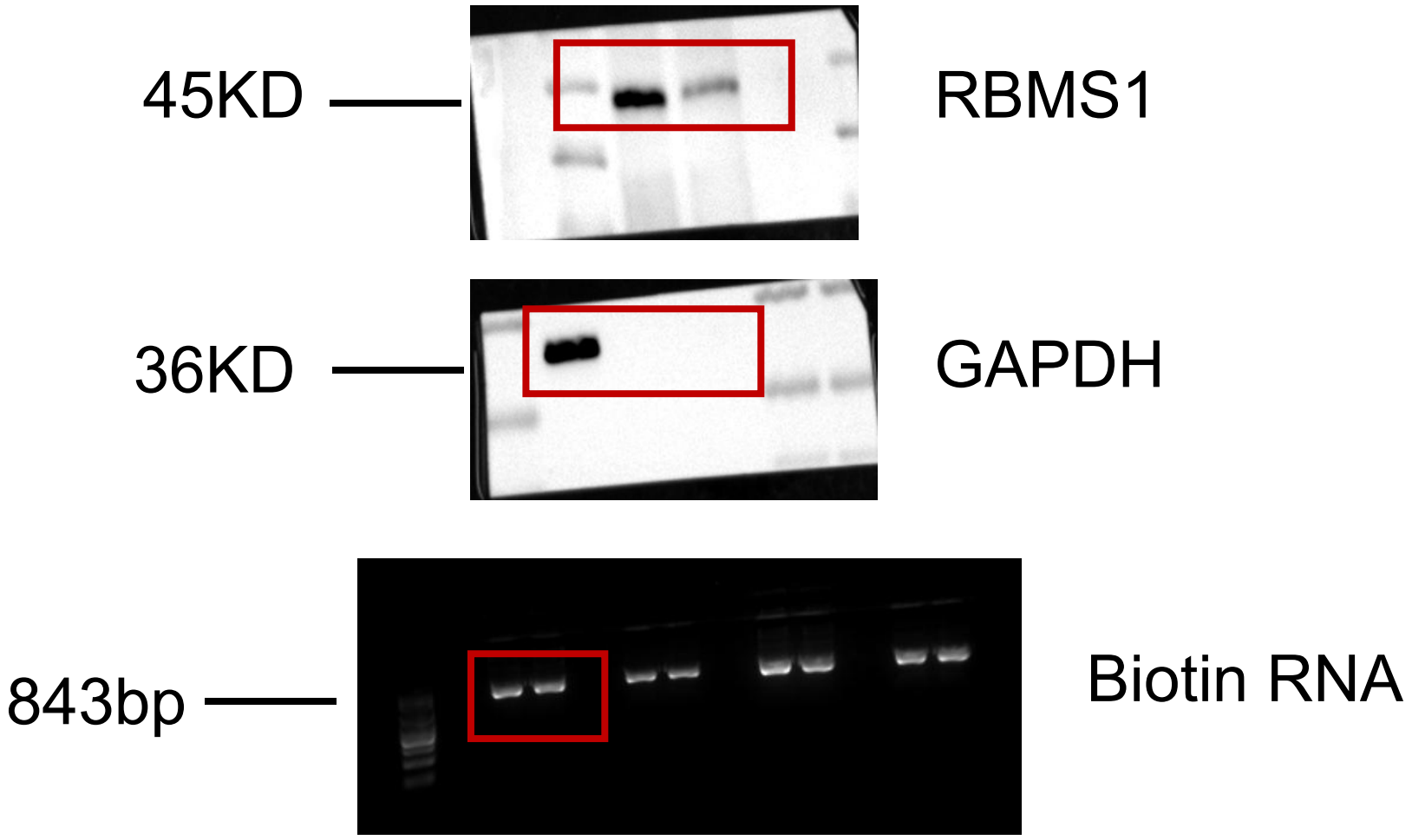

Figure. 3O

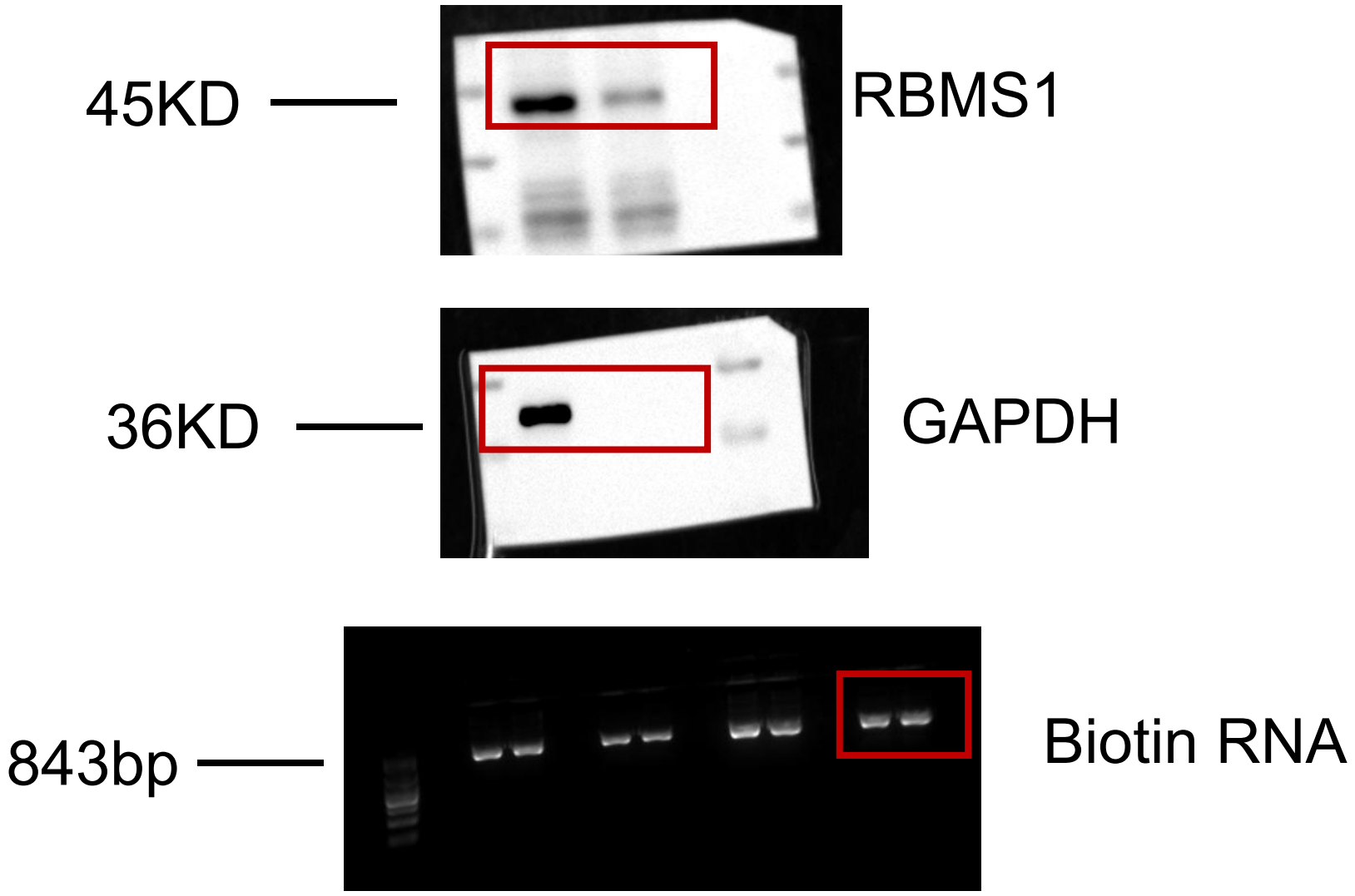

Original data of the corresponding images in Figure 4

Figure. 4A

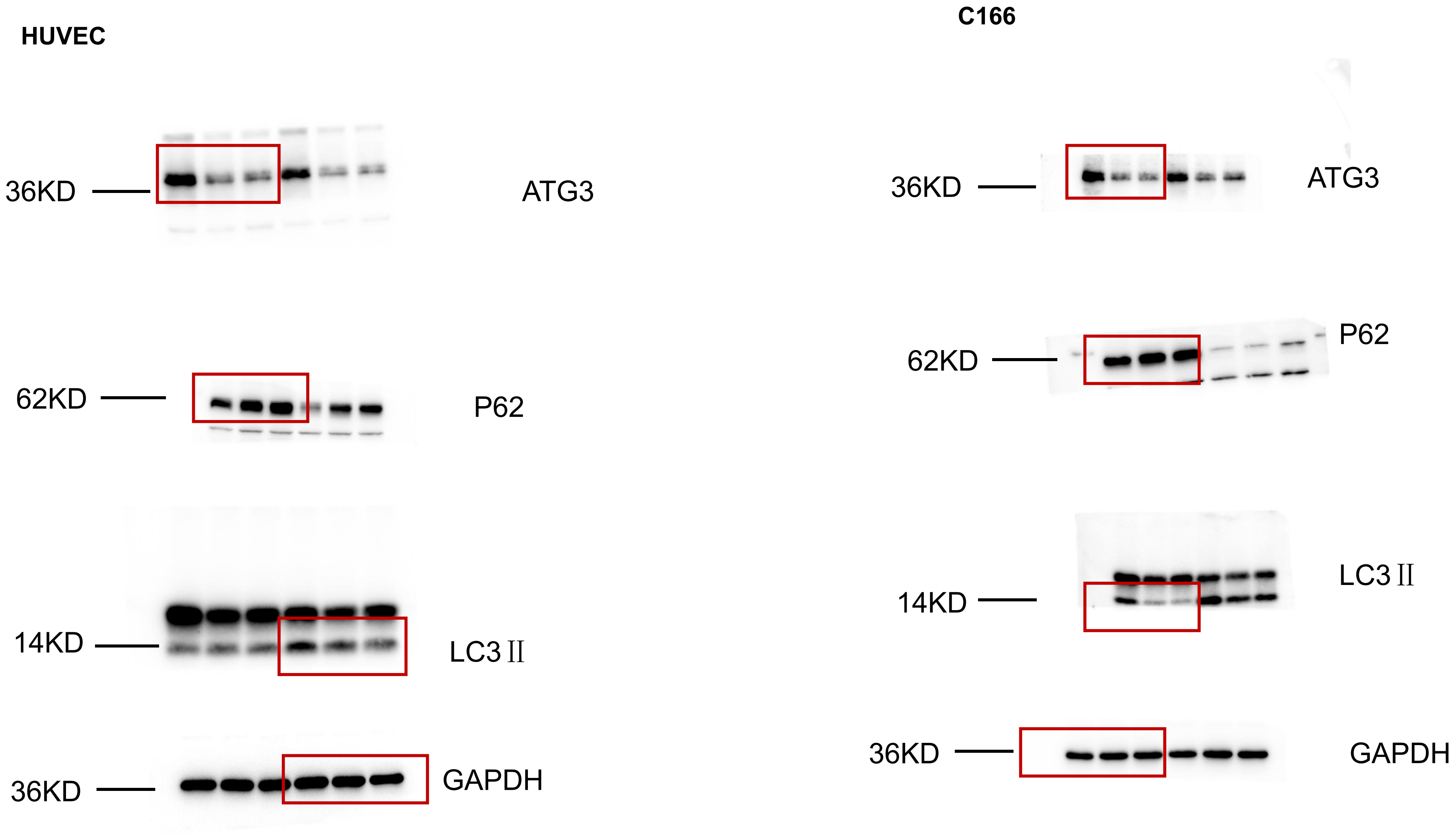

Figure. 4B

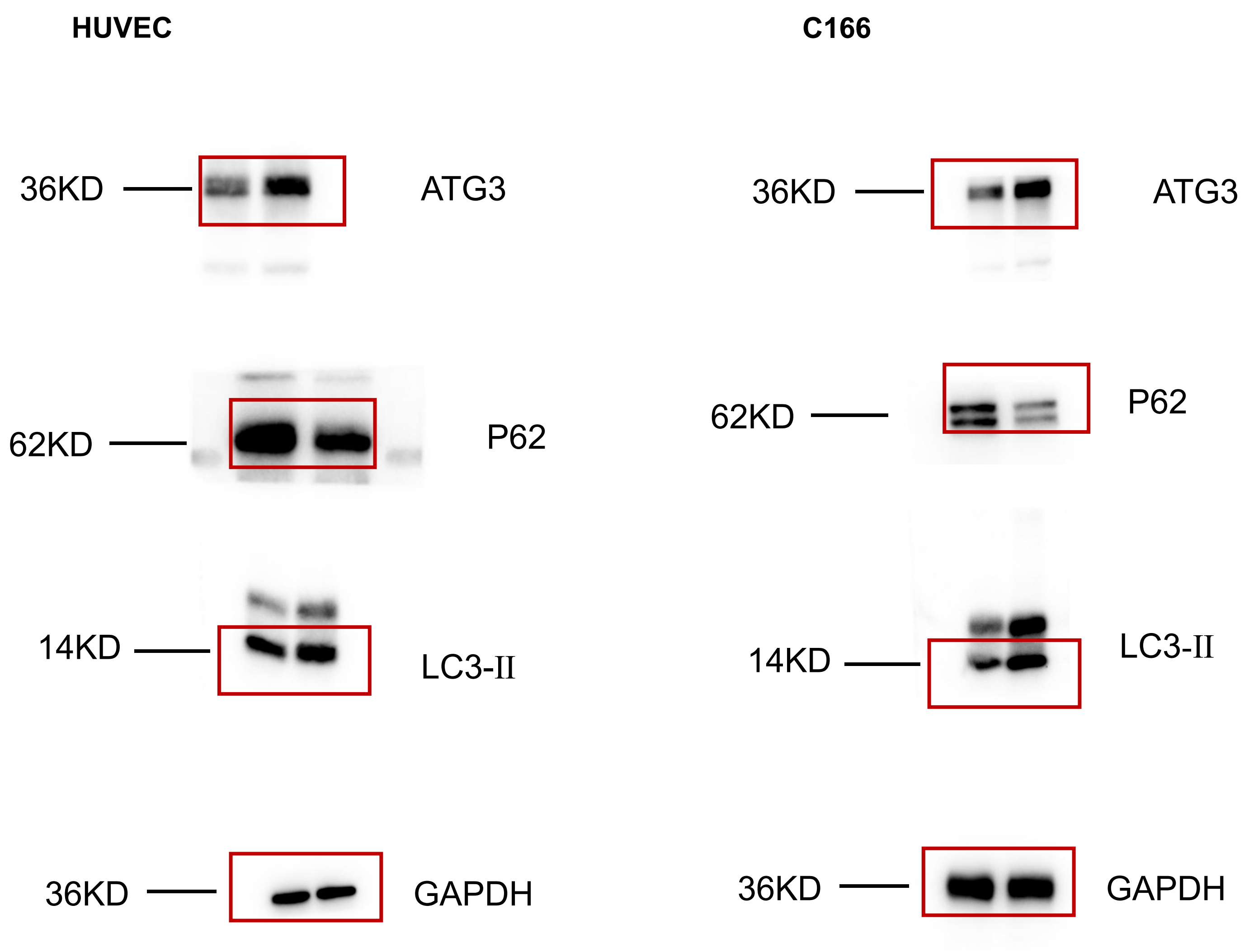

HUVEC
C166
36KD
ATG3
36KD
ATG3
62KD
P62
62KD
P62
14KD
LC3-II
14KD
LC3-II
36KD
GAPDH
36KD
GAPDH

Figure. 4G

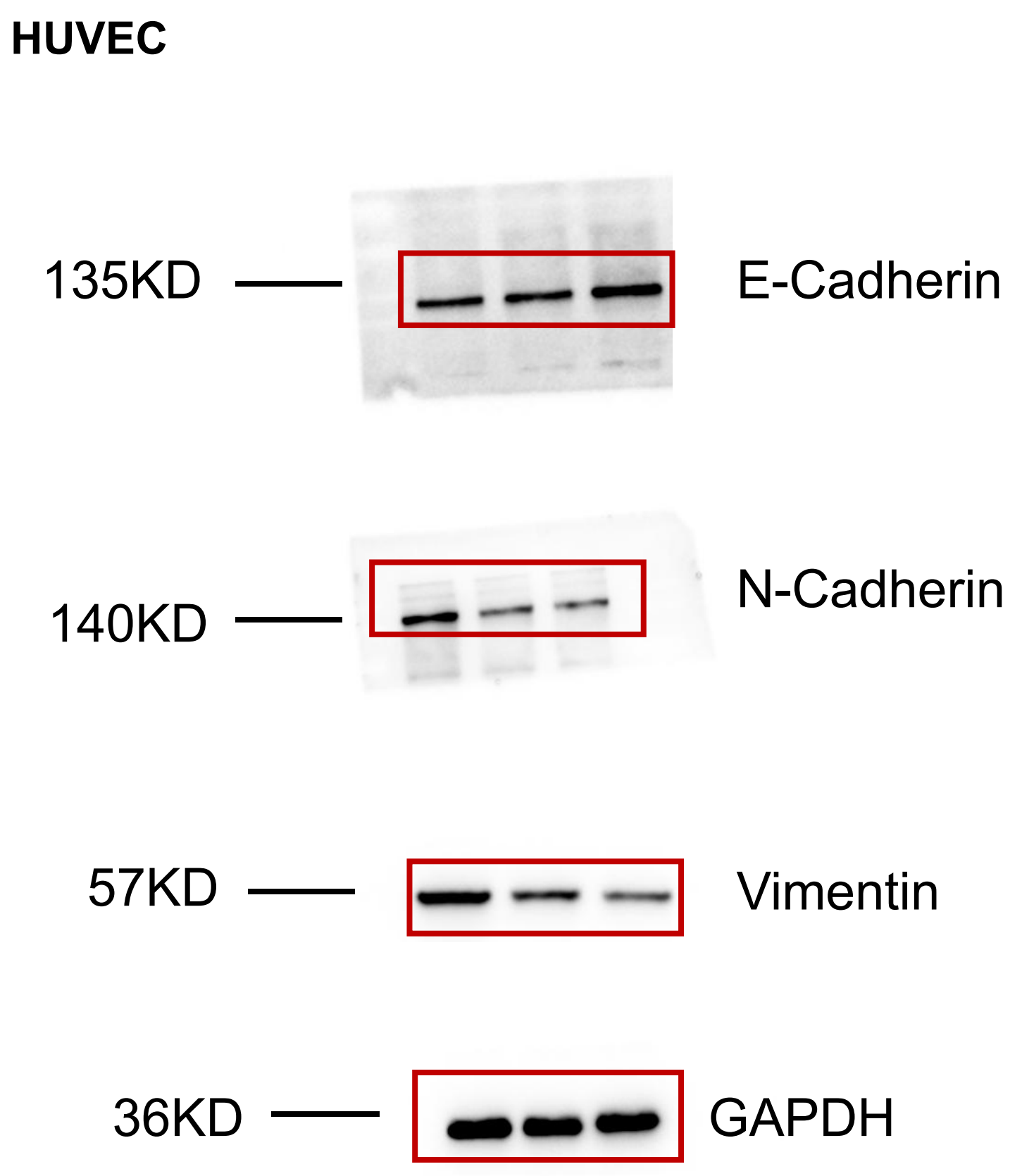
HUVEC
135KD
E-Cadherin
140KD
N-Cadherin
57KD
Vimentin
36KD
GAPDH

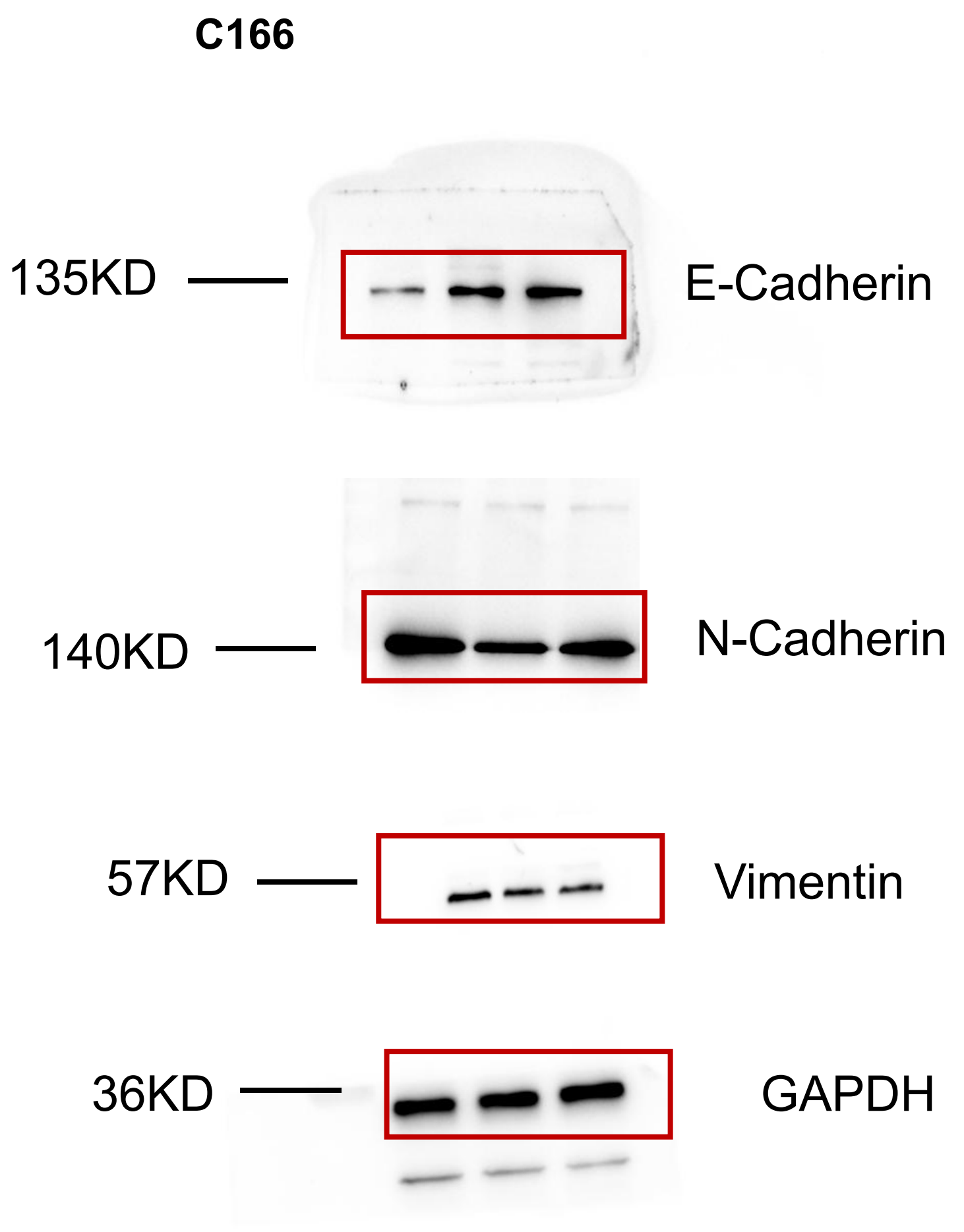
C166
135KD
E-Cadherin
140KD
N-Cadherin
57KD
Vimentin
36KD
GAPDH

Figure. 4H

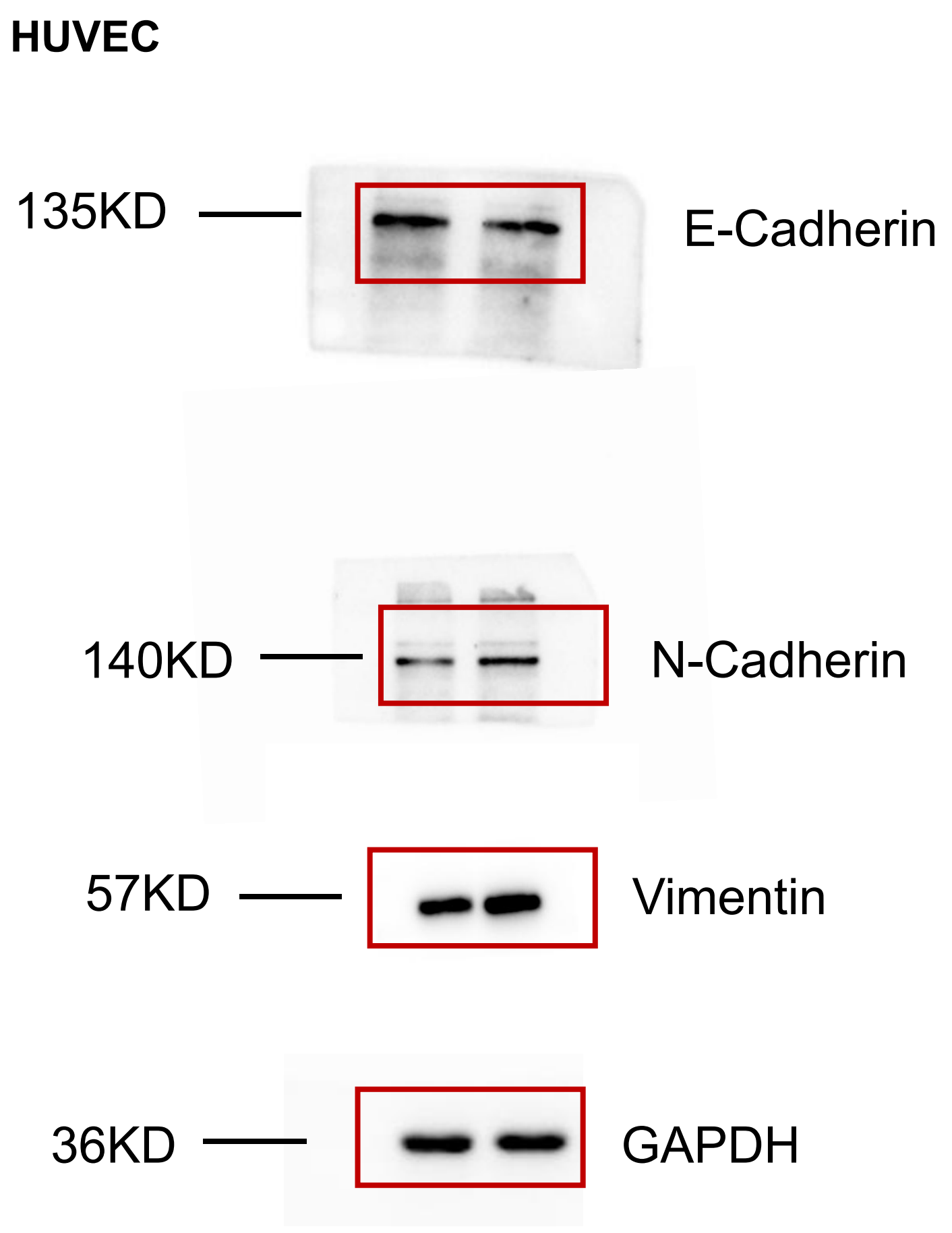
HUVEC
135KD
E-Cadherin
140KD
N-Cadherin
57KD
Vimentin
36KD
GAPDH

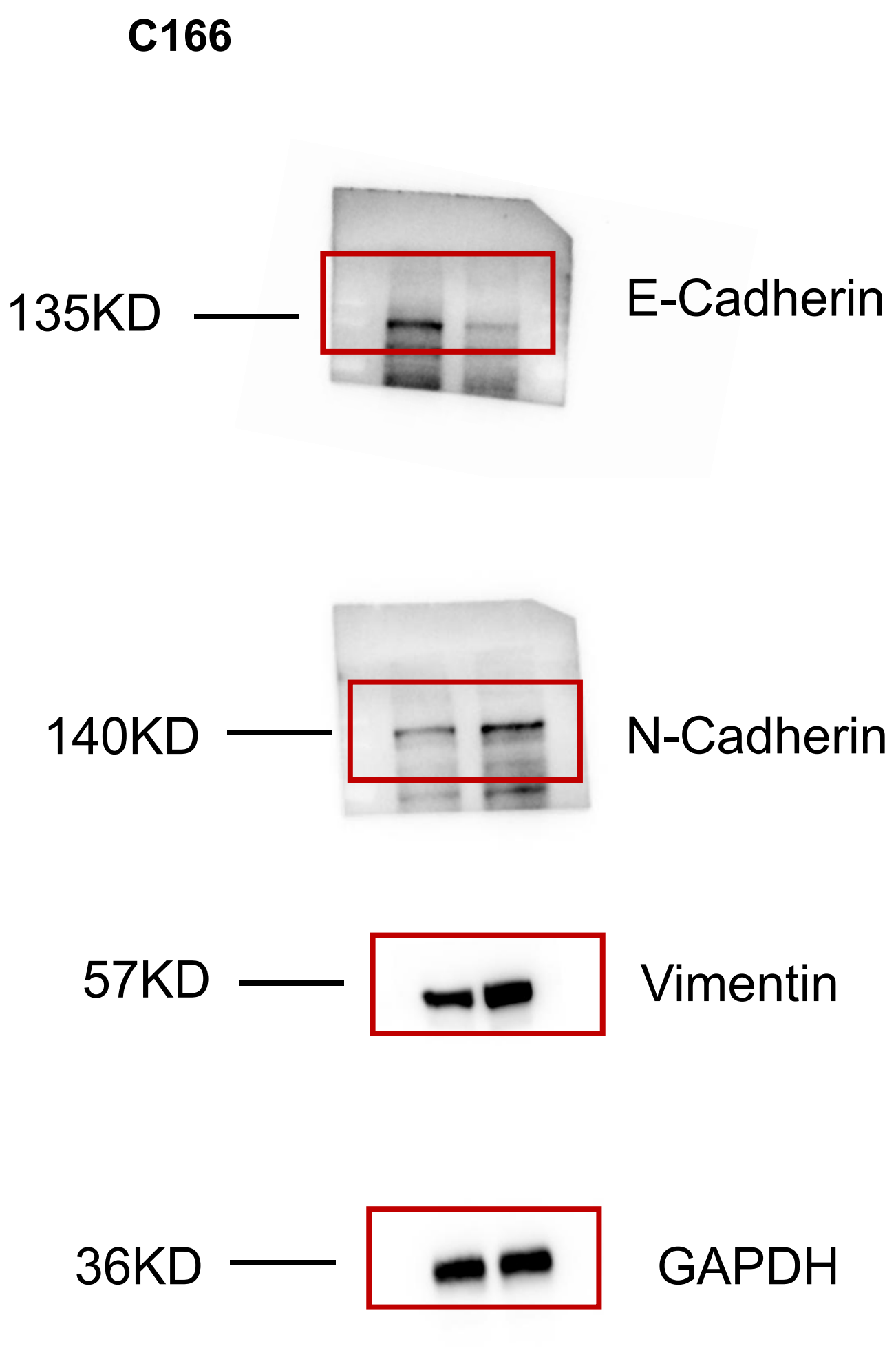
C166
135KD
E-Cadherin
140KD
N-Cadherin
57KD
Vimentin
36KD
GAPDH

Figure. 4E

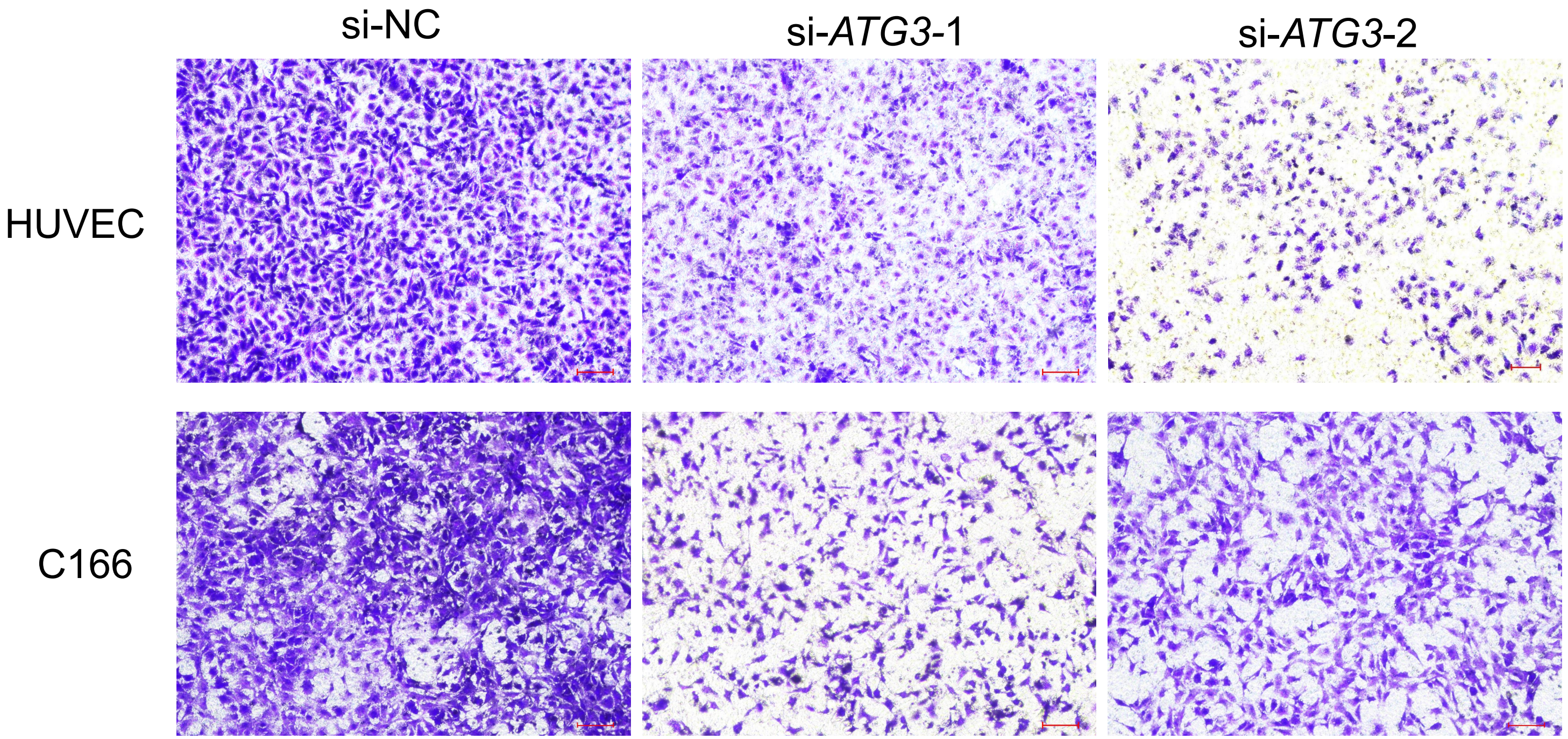

Figure. 4F

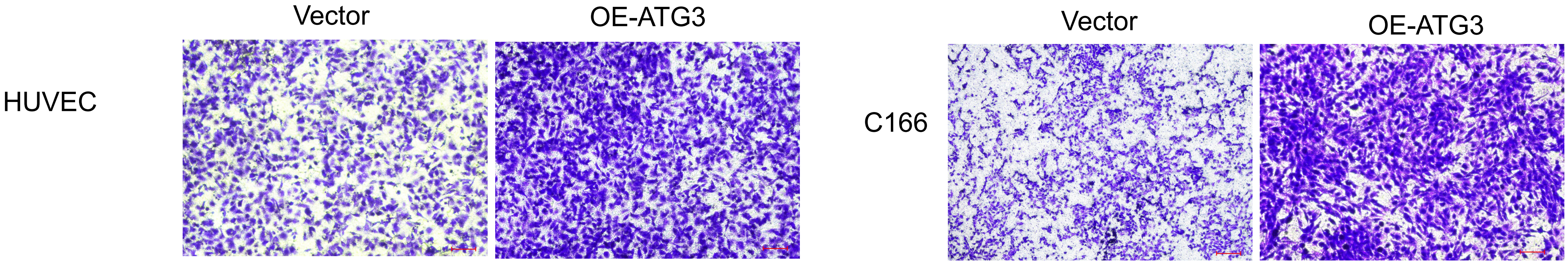

Figure. 4I

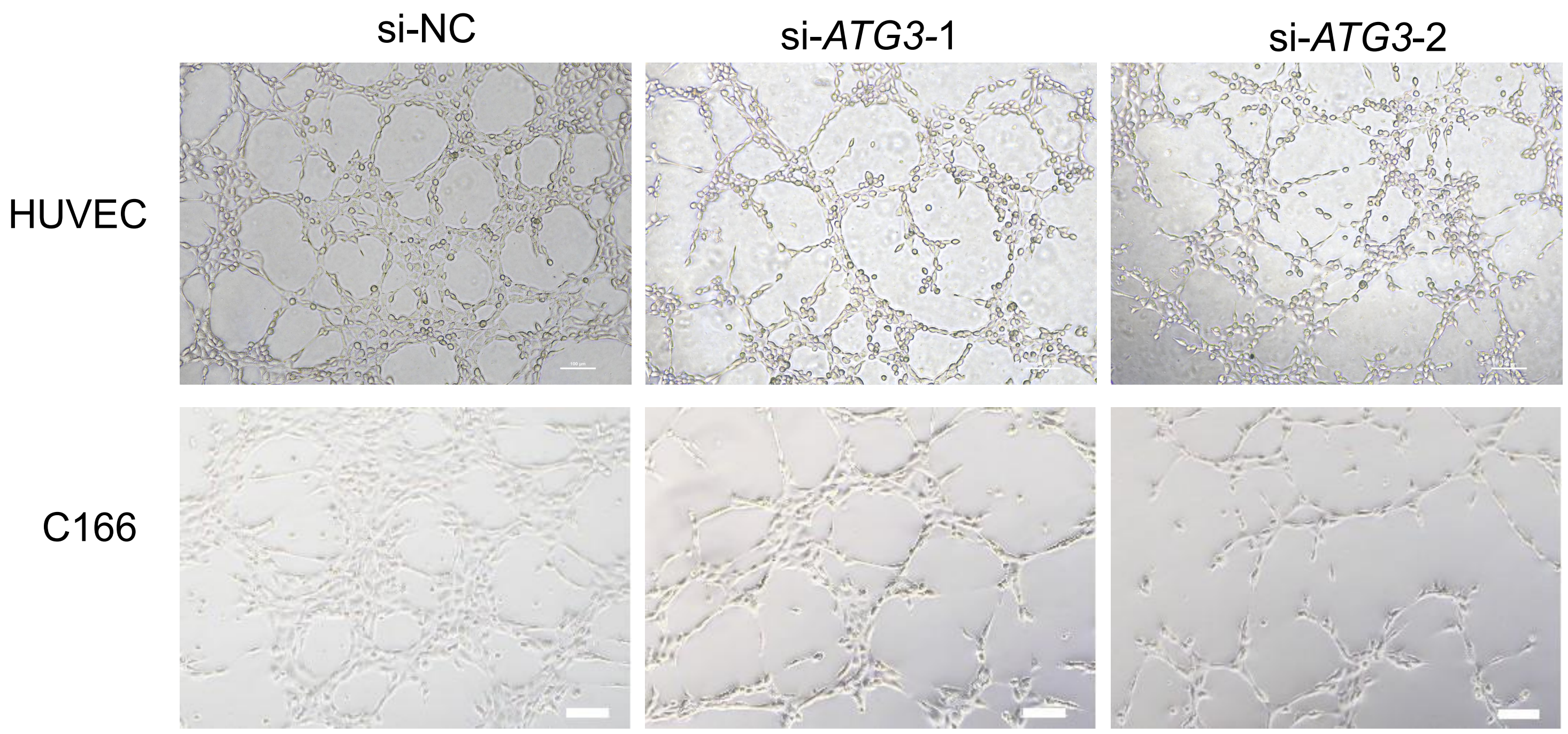

Figure. 4J

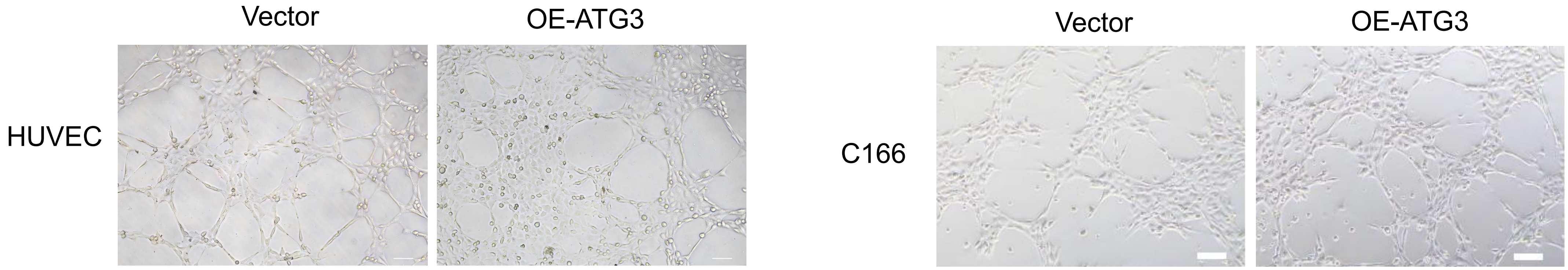

Original data of the corresponding images in Figure 5

Figure. 5E

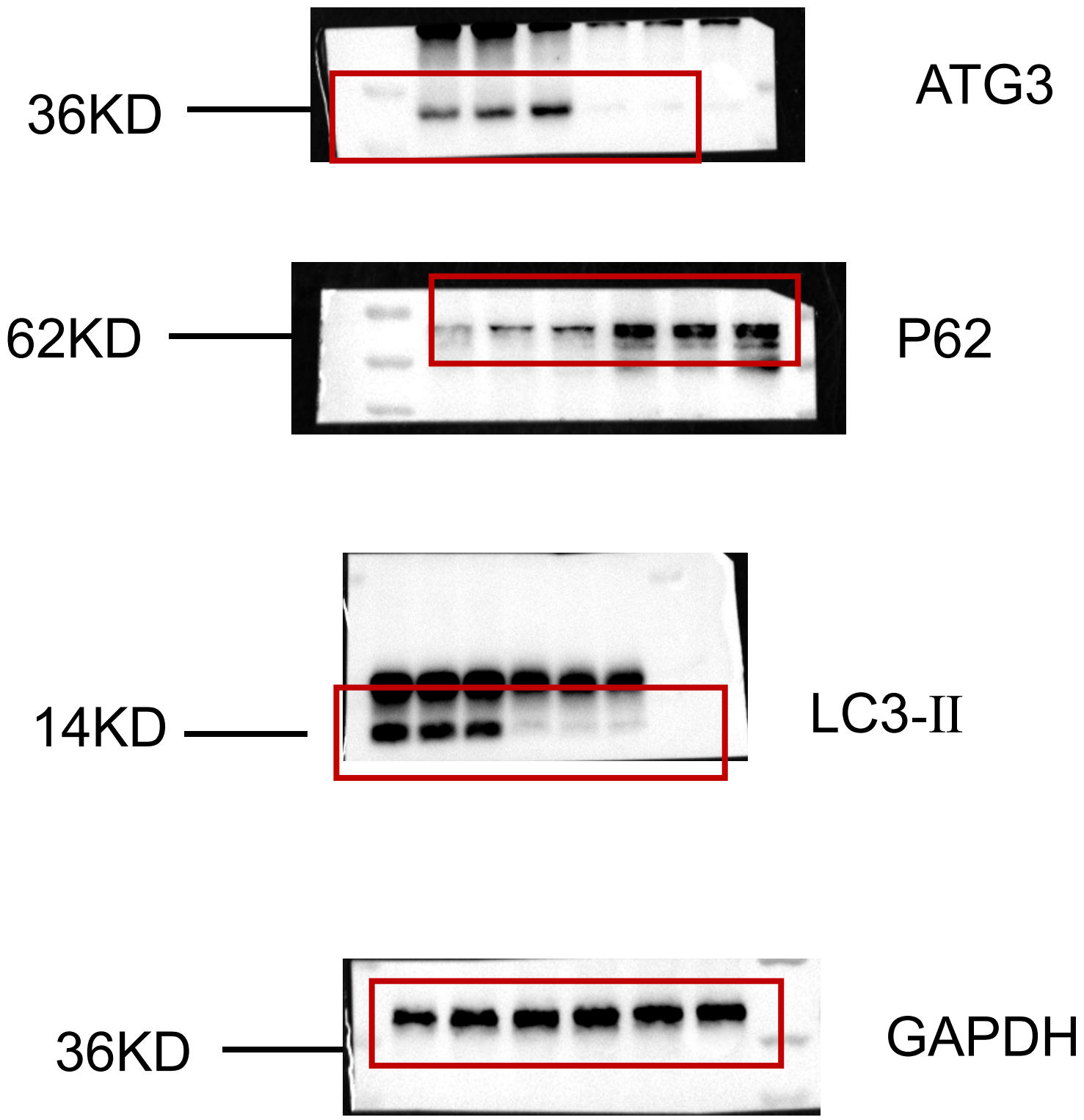

Figure. 5F

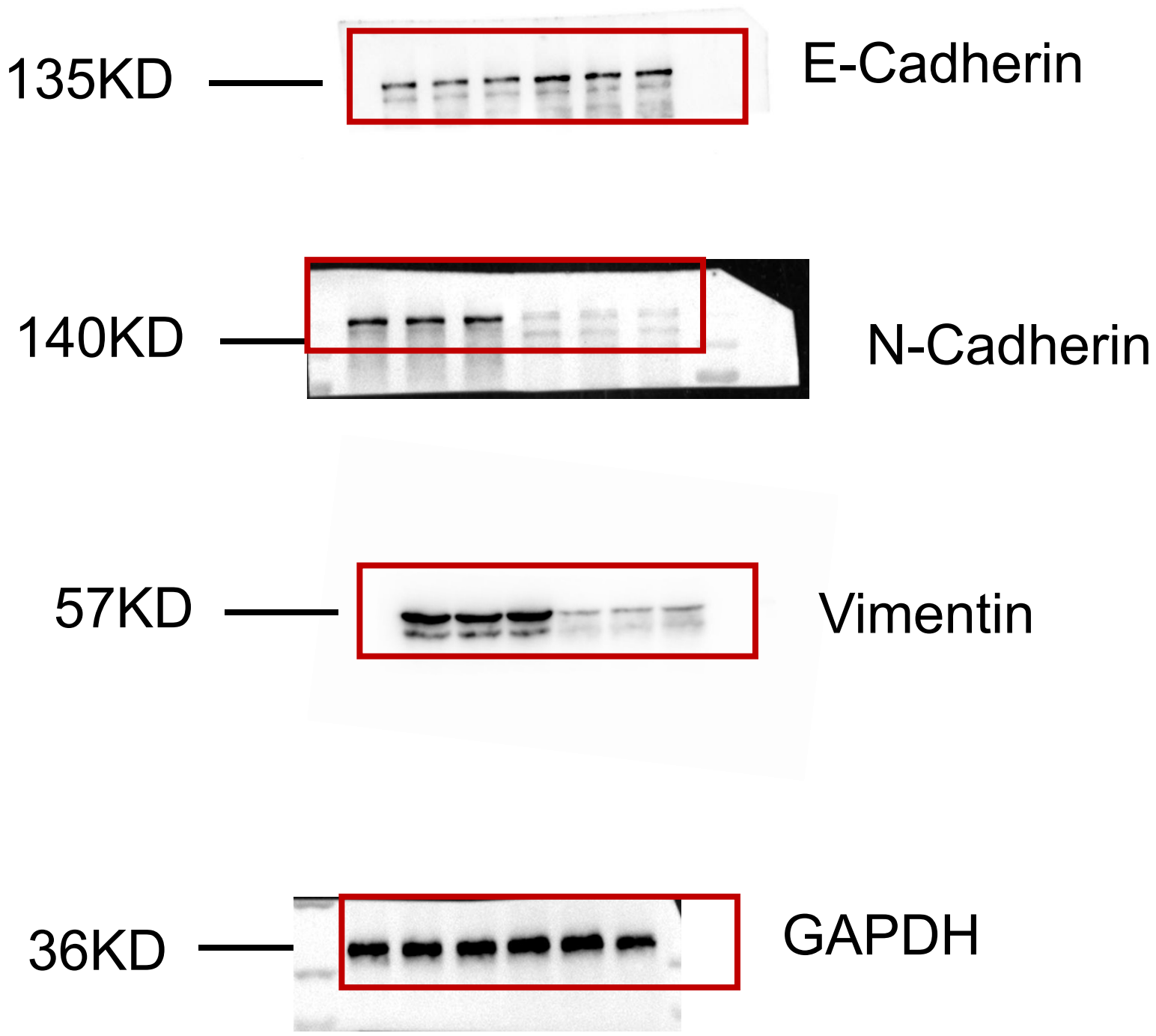

Figure. 5A

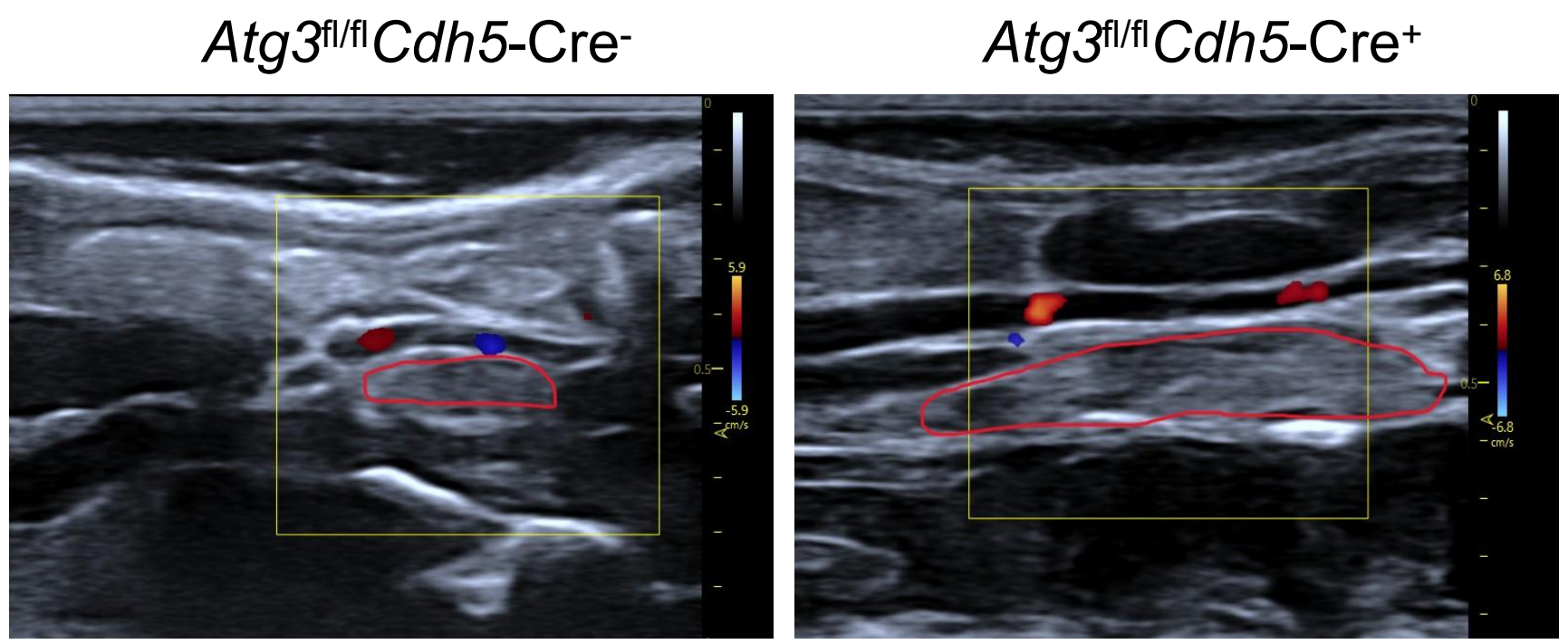

Figure. 5C

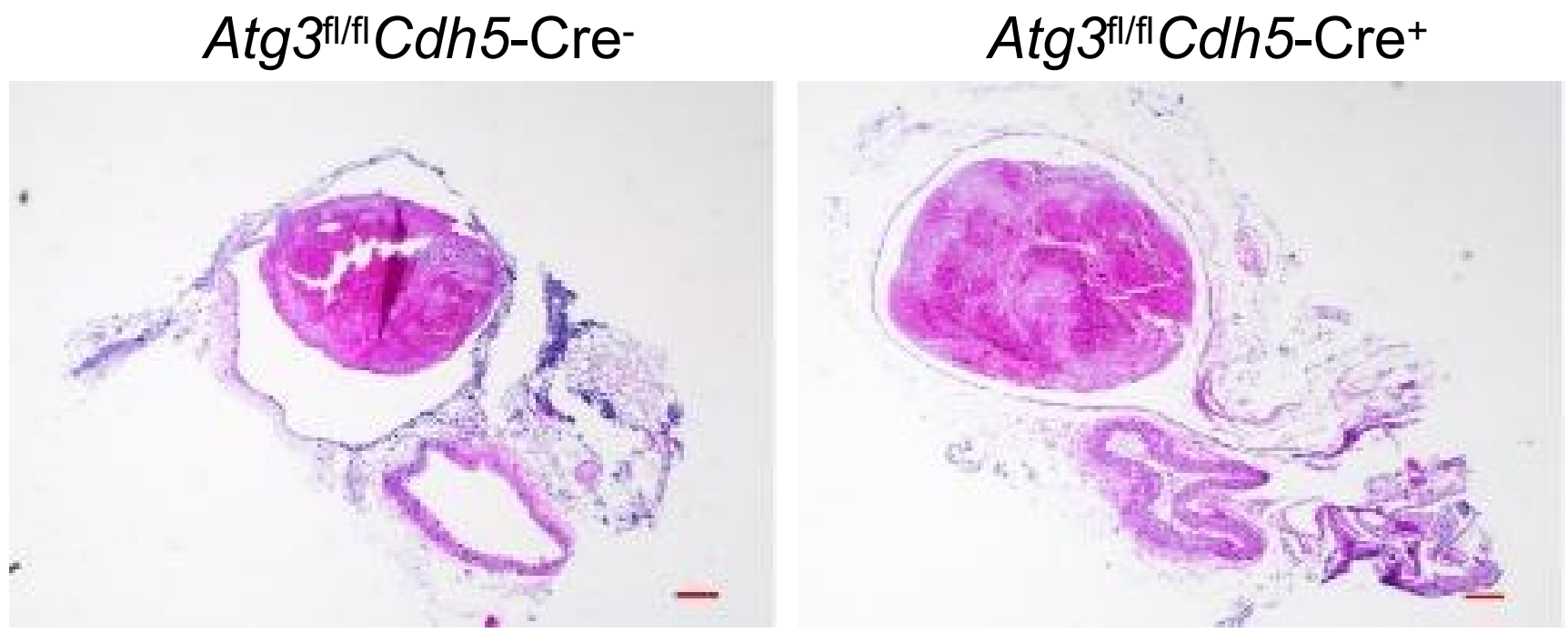

Original data of the corresponding images in Figure 6

Figure. 6C

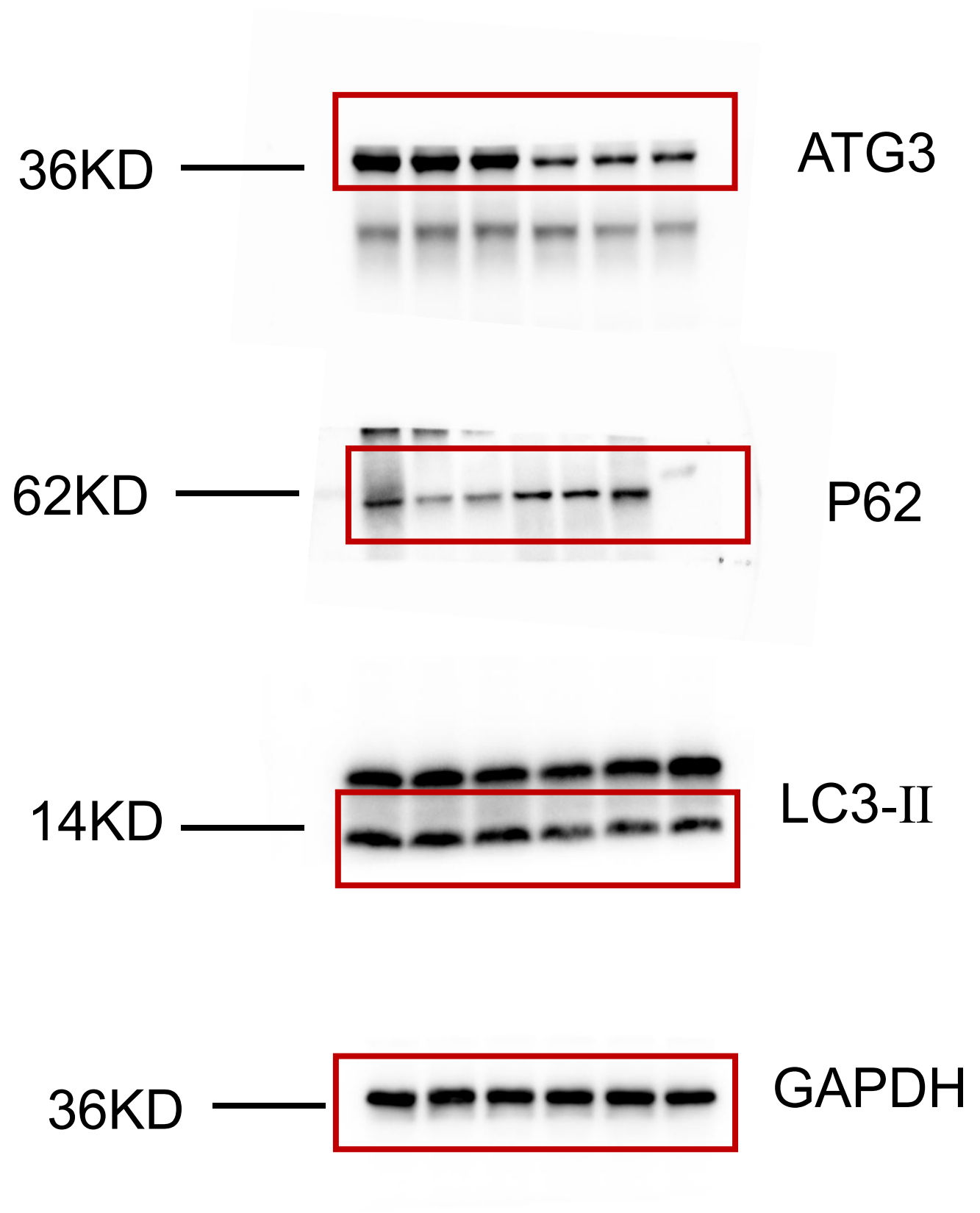

Figure. 6D

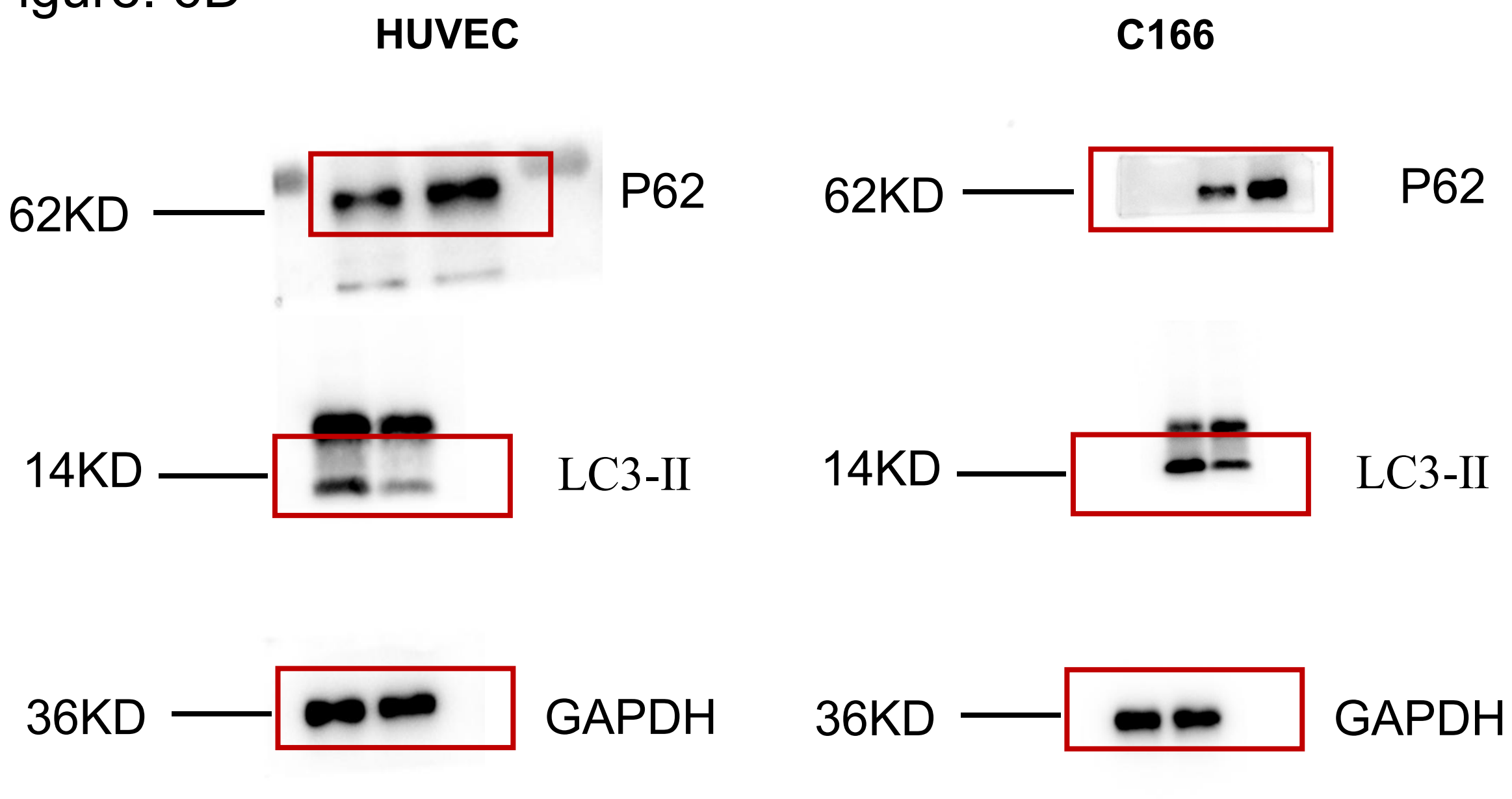

Figure. 6G

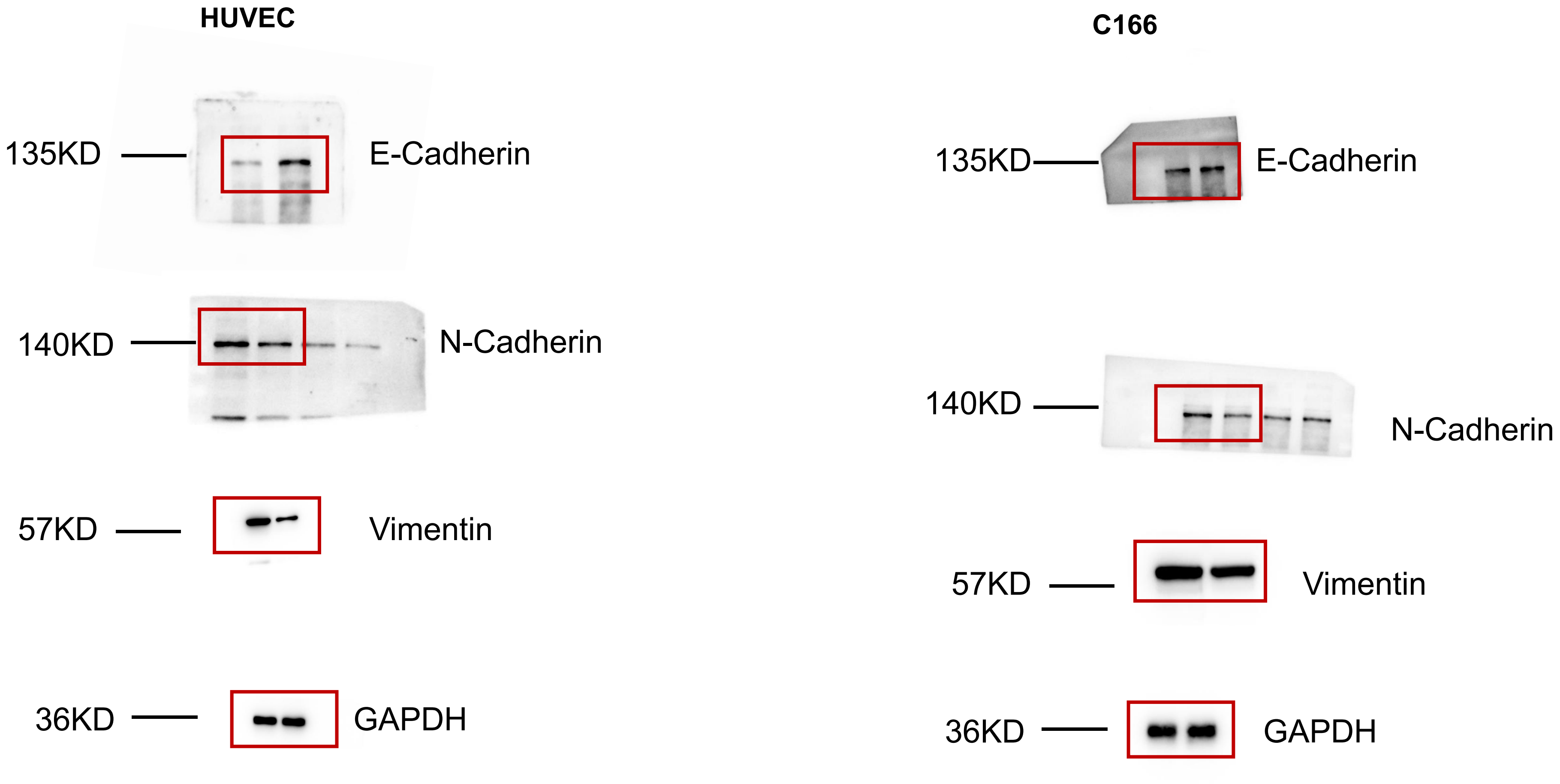

HUVEC
135KD
E-Cadherin
140KD
N-Cadherin
57KD
Vimentin
36KD
GAPDH
C166
135KD
E-Cadherin
140KD
N-Cadherin
57KD
Vimentin
36KD
GAPDH

Figure. 6H

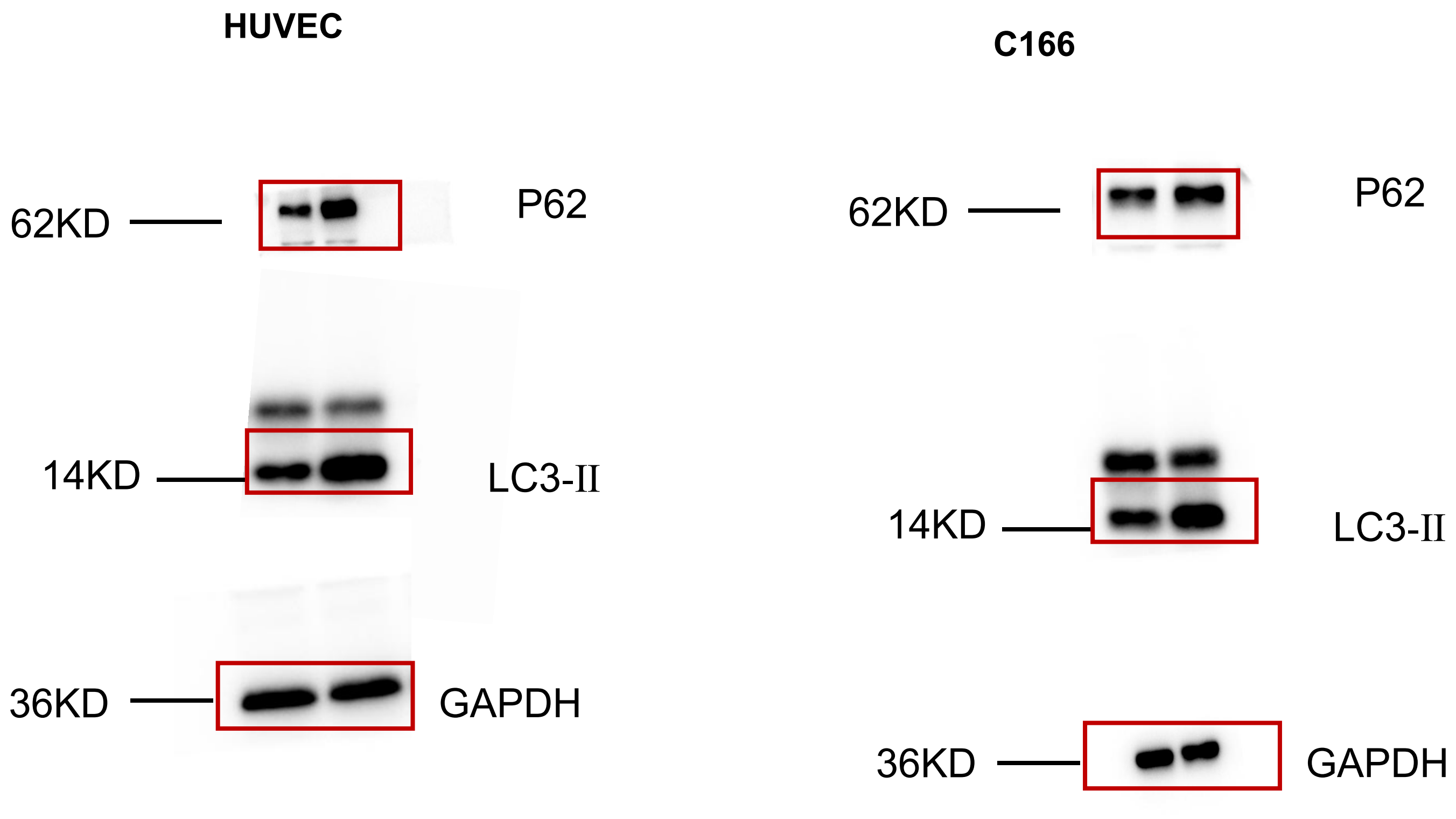
HUVEC
C166
62KD
P62
14KD
LC3-II
36KD
GAPDH
62KD
P62
14KD
LC3-II
36KD
GAPDH

Figure. 6K

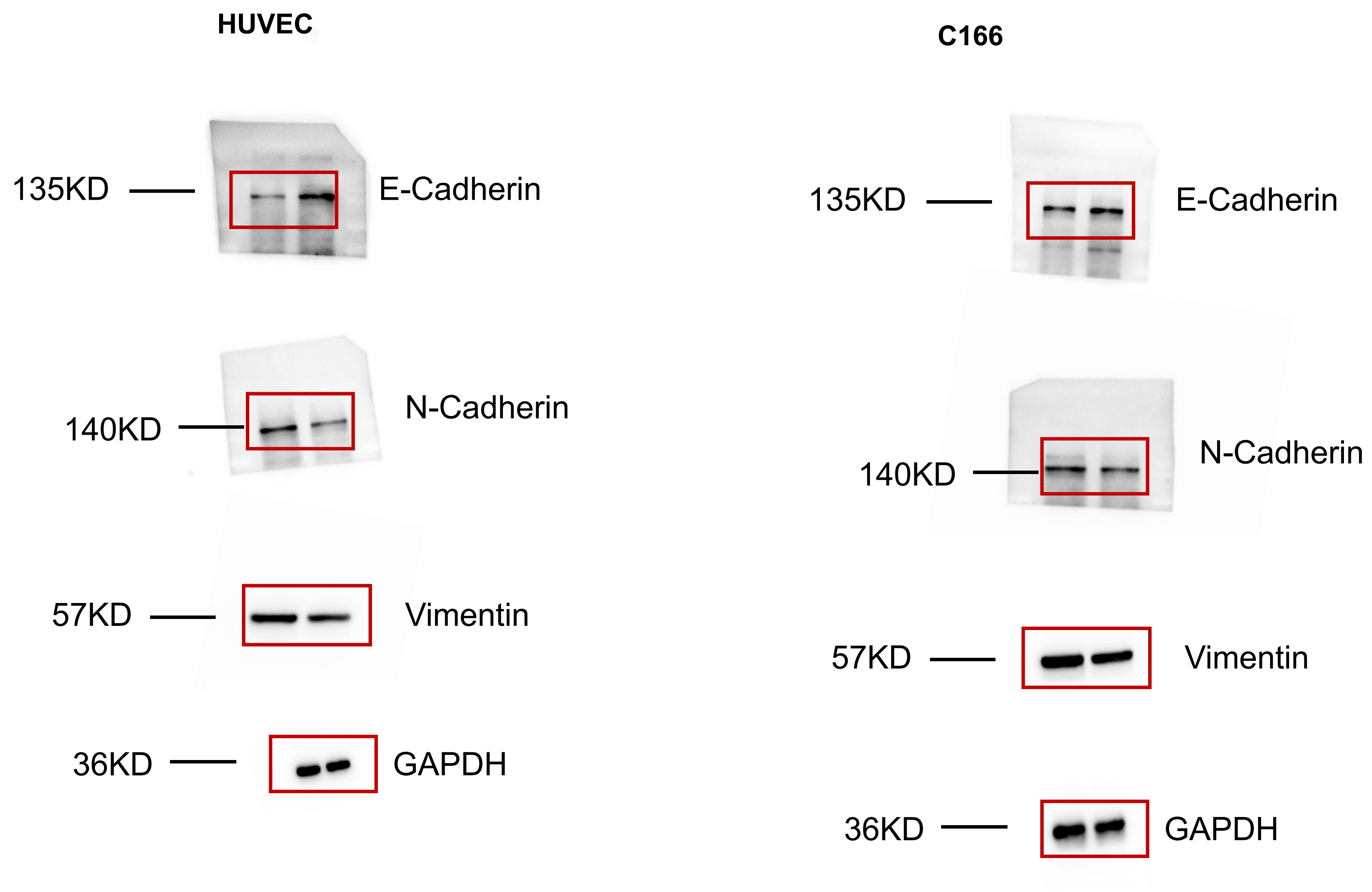
HUVEC
135KD
E-Cadherin
140KD
N-Cadherin
57KD
Vimentin
36KD
GAPDH
C166
135KD
E-Cadherin
140KD
N-Cadherin
57KD
Vimentin
36KD
GAPDH

Figure. 6B

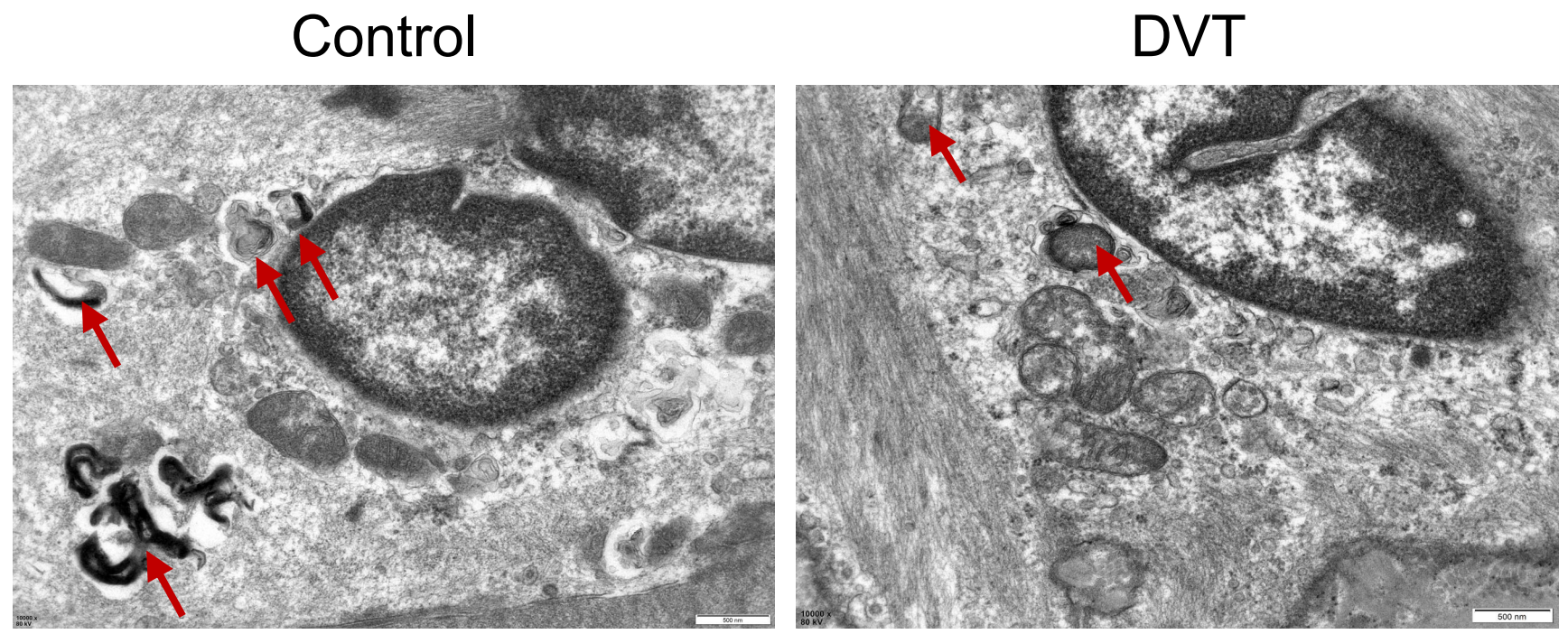

Figure. 6F

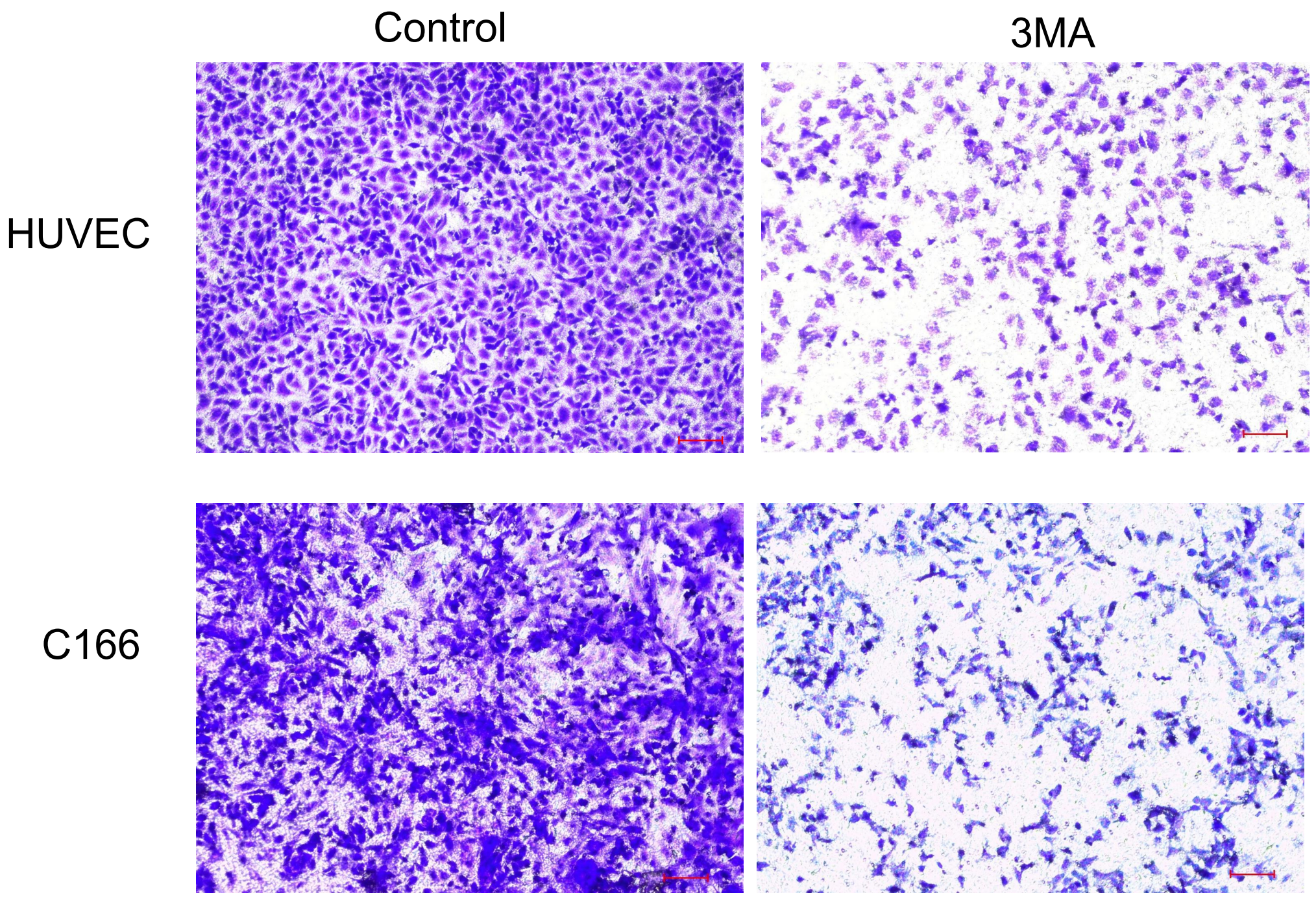

Figure. 6J

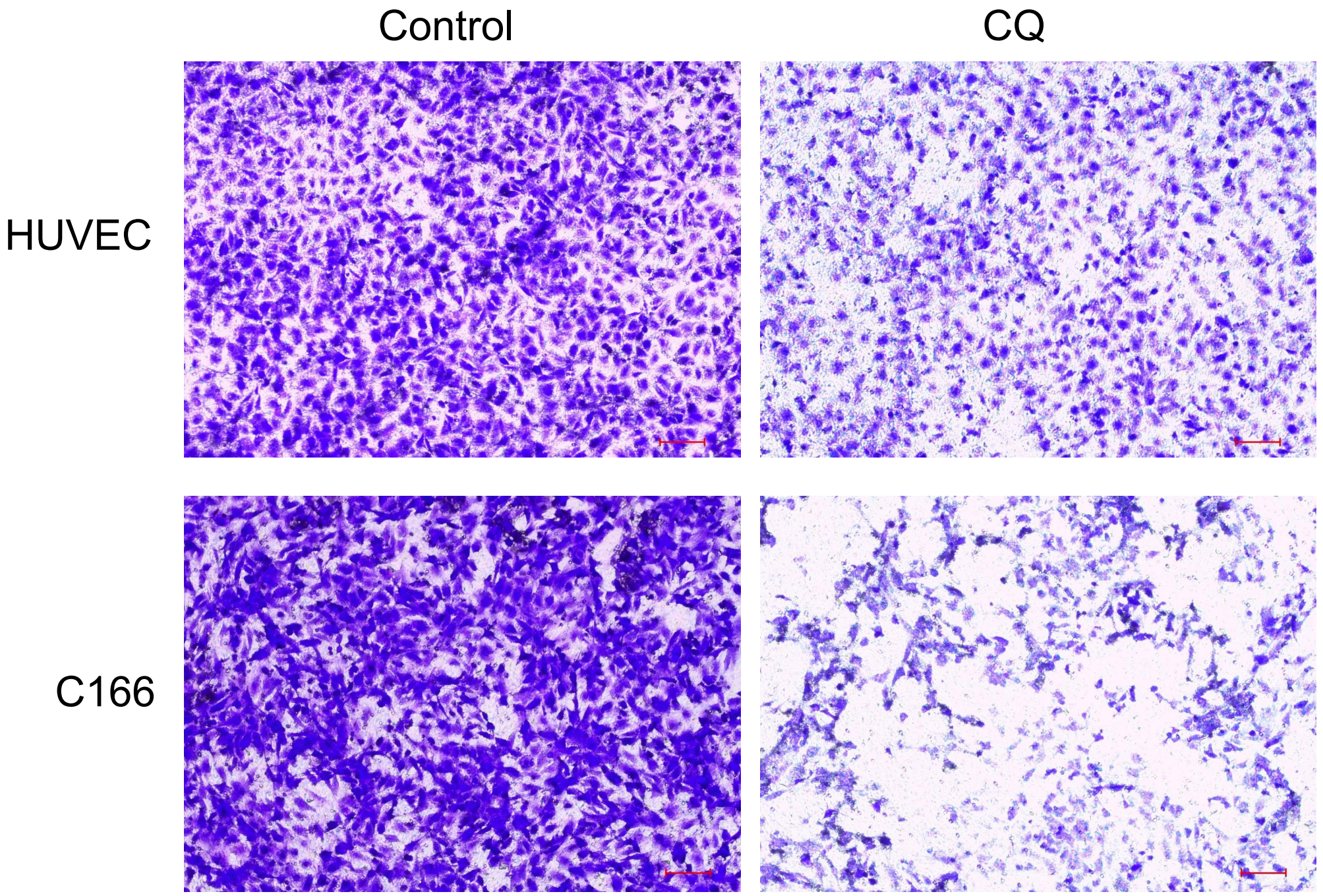

Figure. 6L

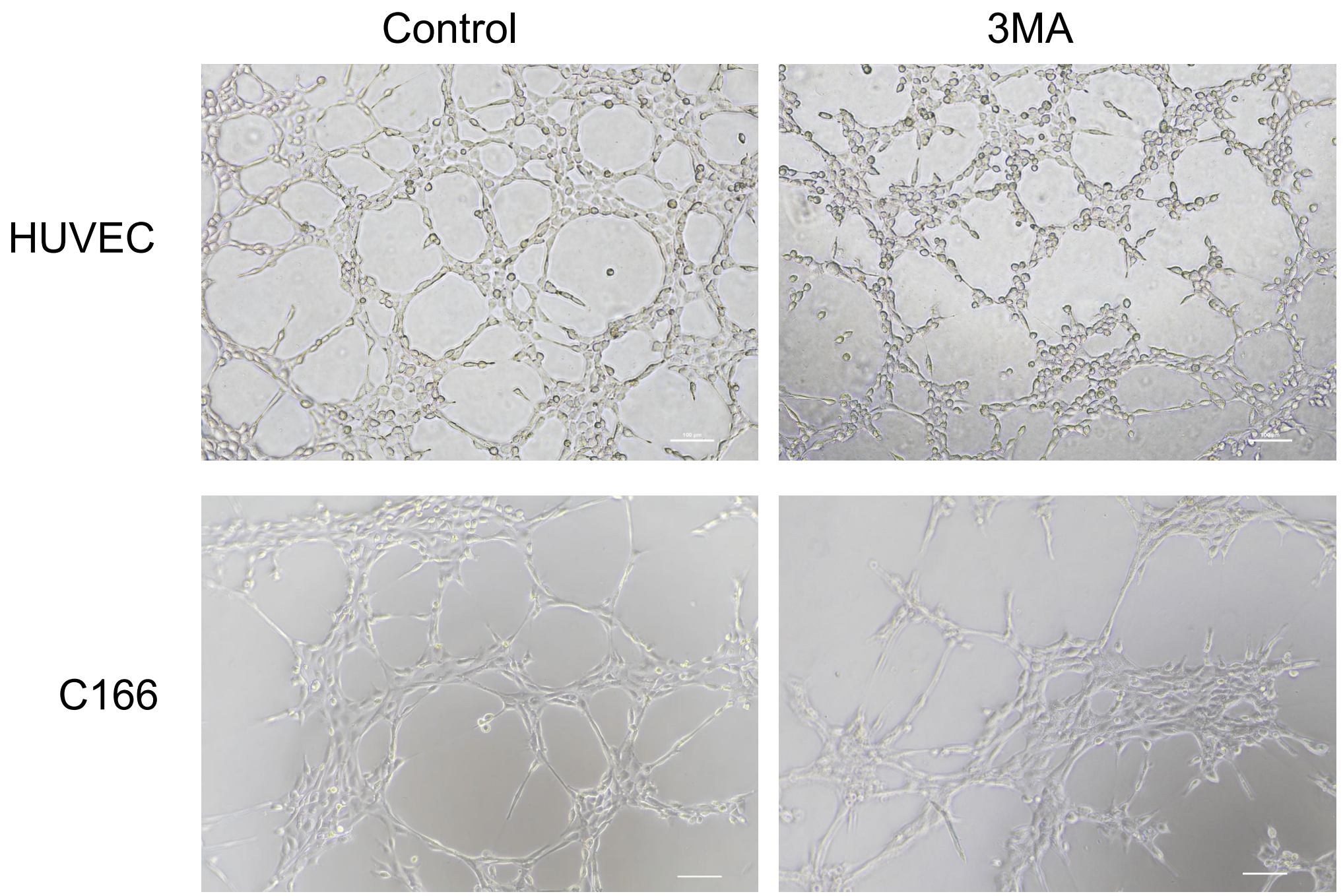

Figure. 6M

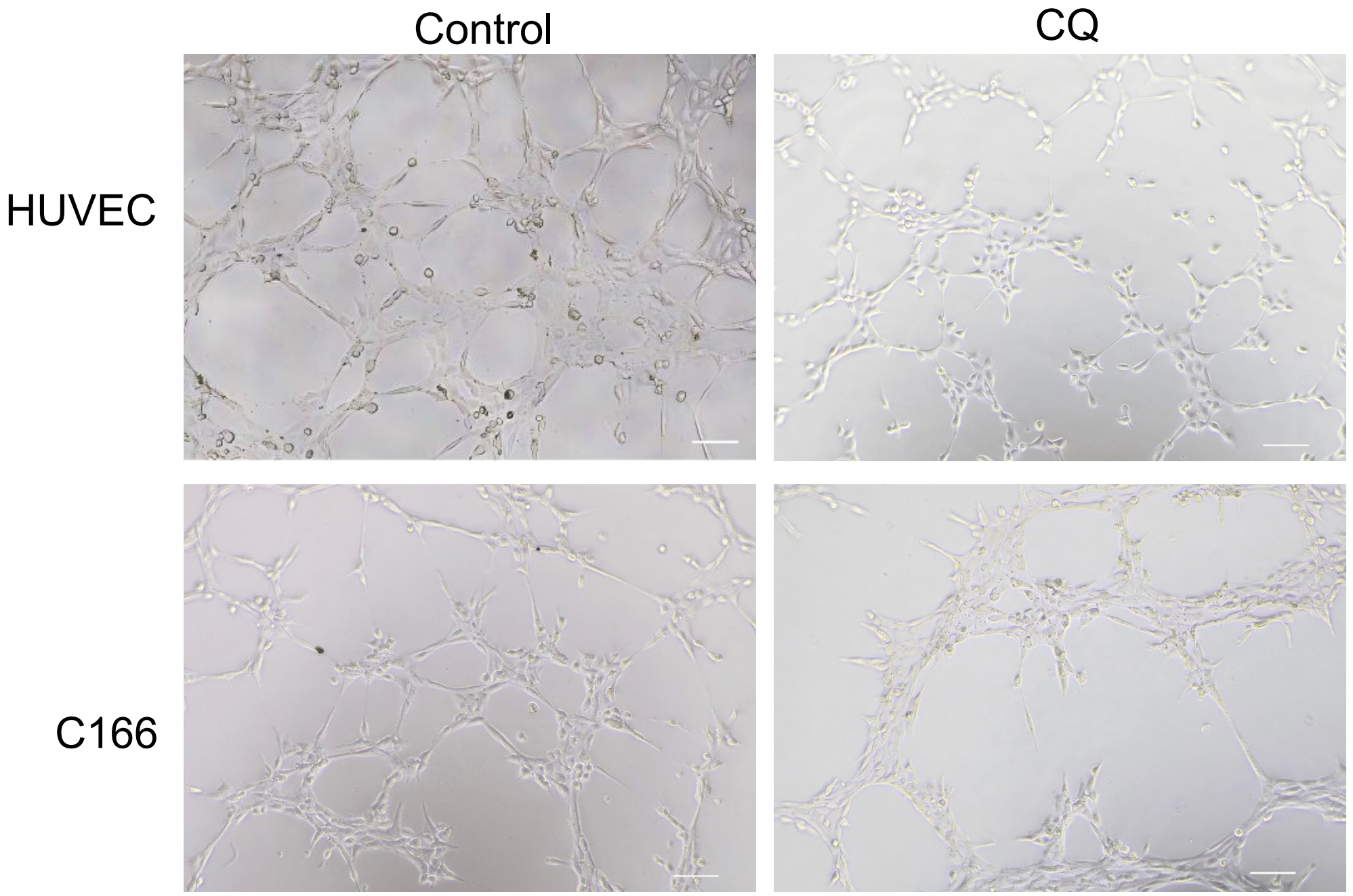

Original data of the corresponding images in Figure7

Figure. 7A

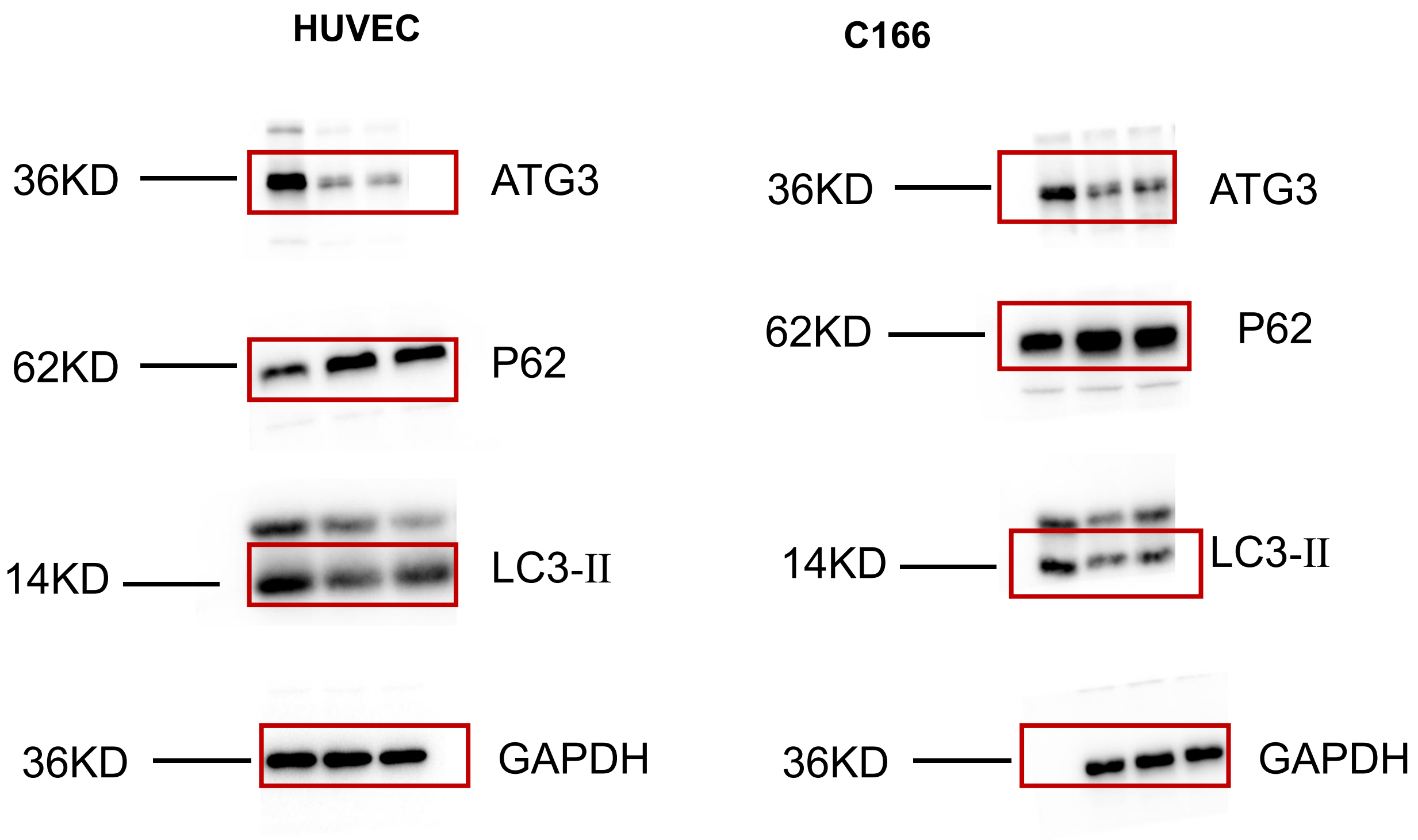

Figure. 7B

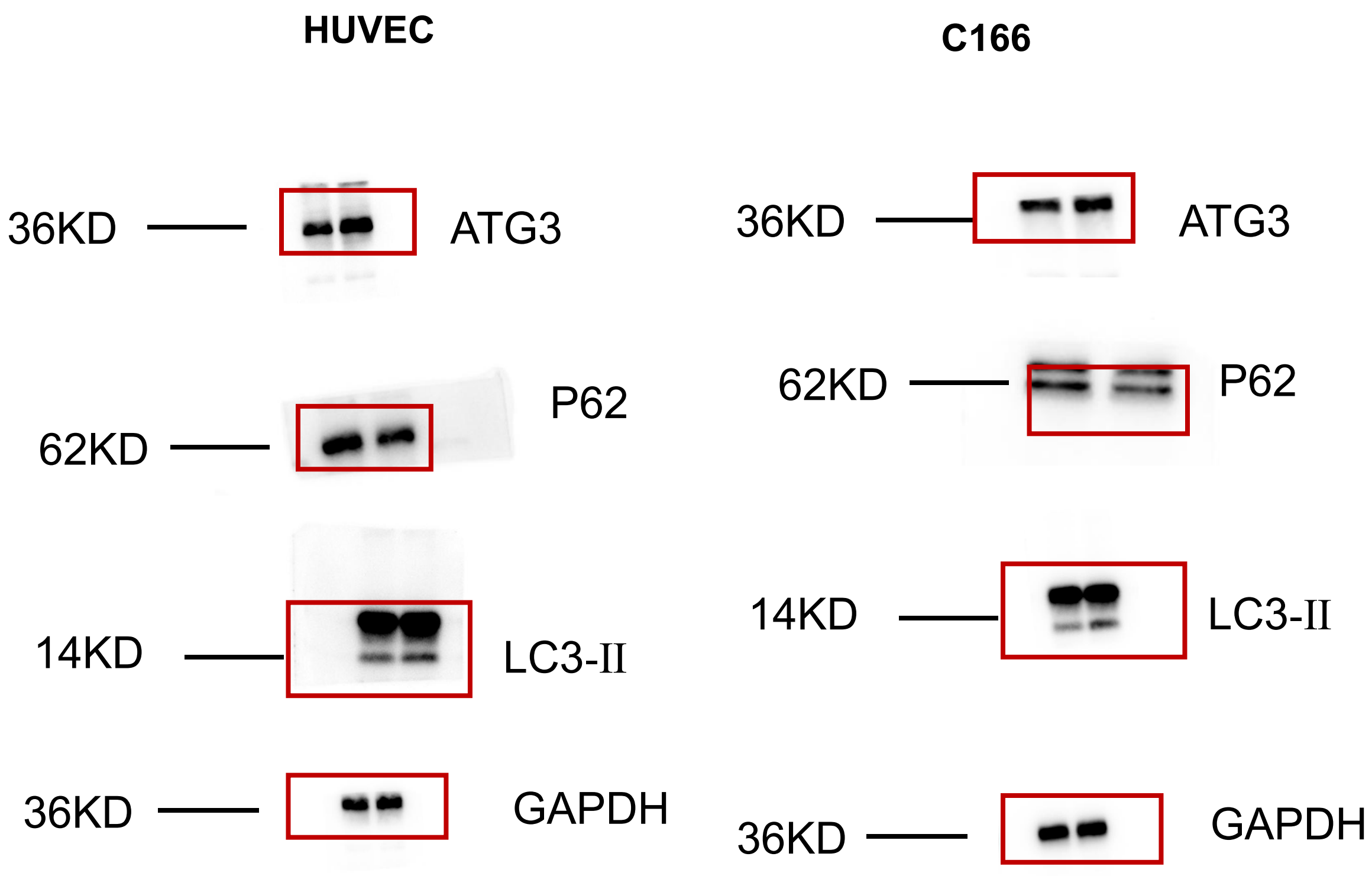

Figure. 7G

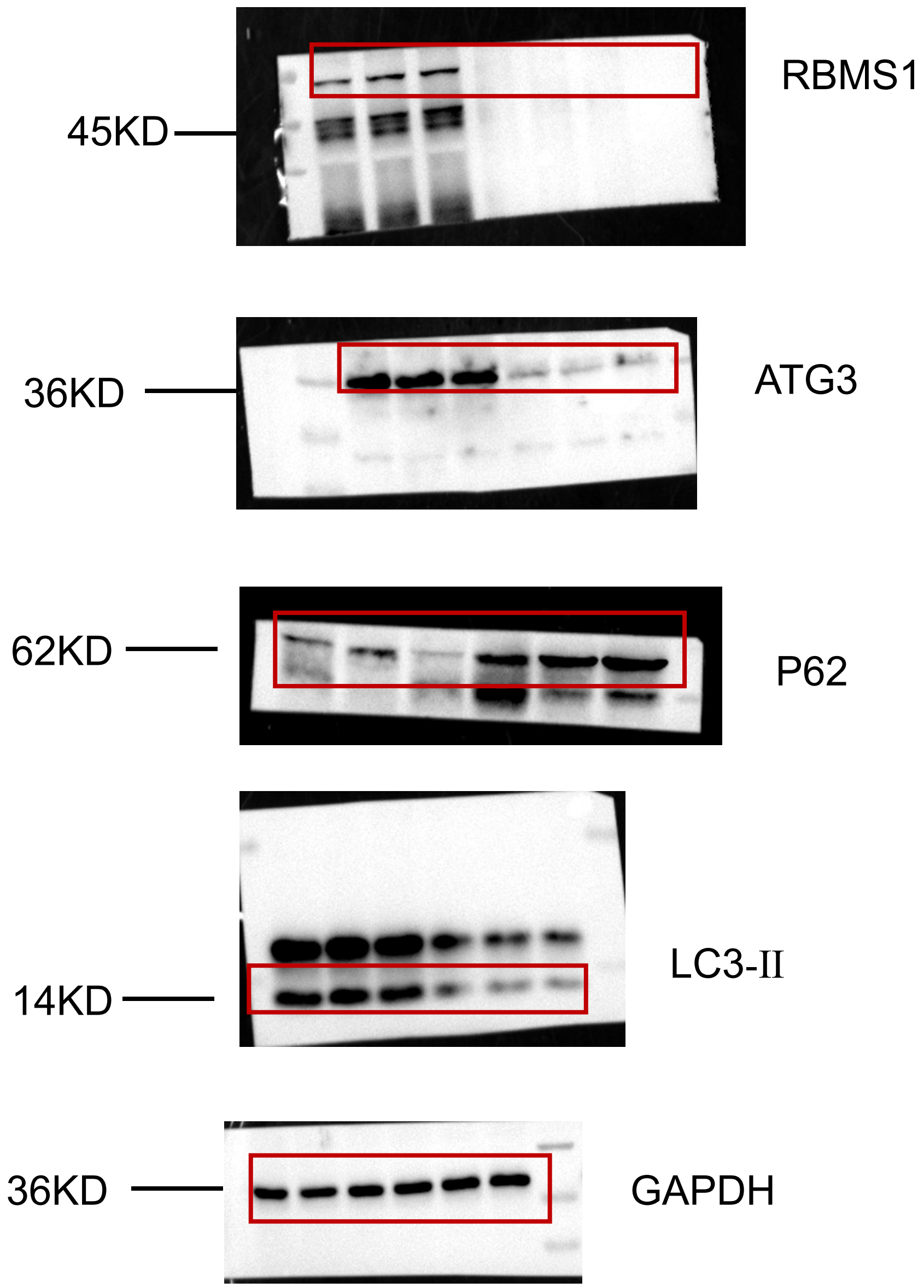

Figure. 7C

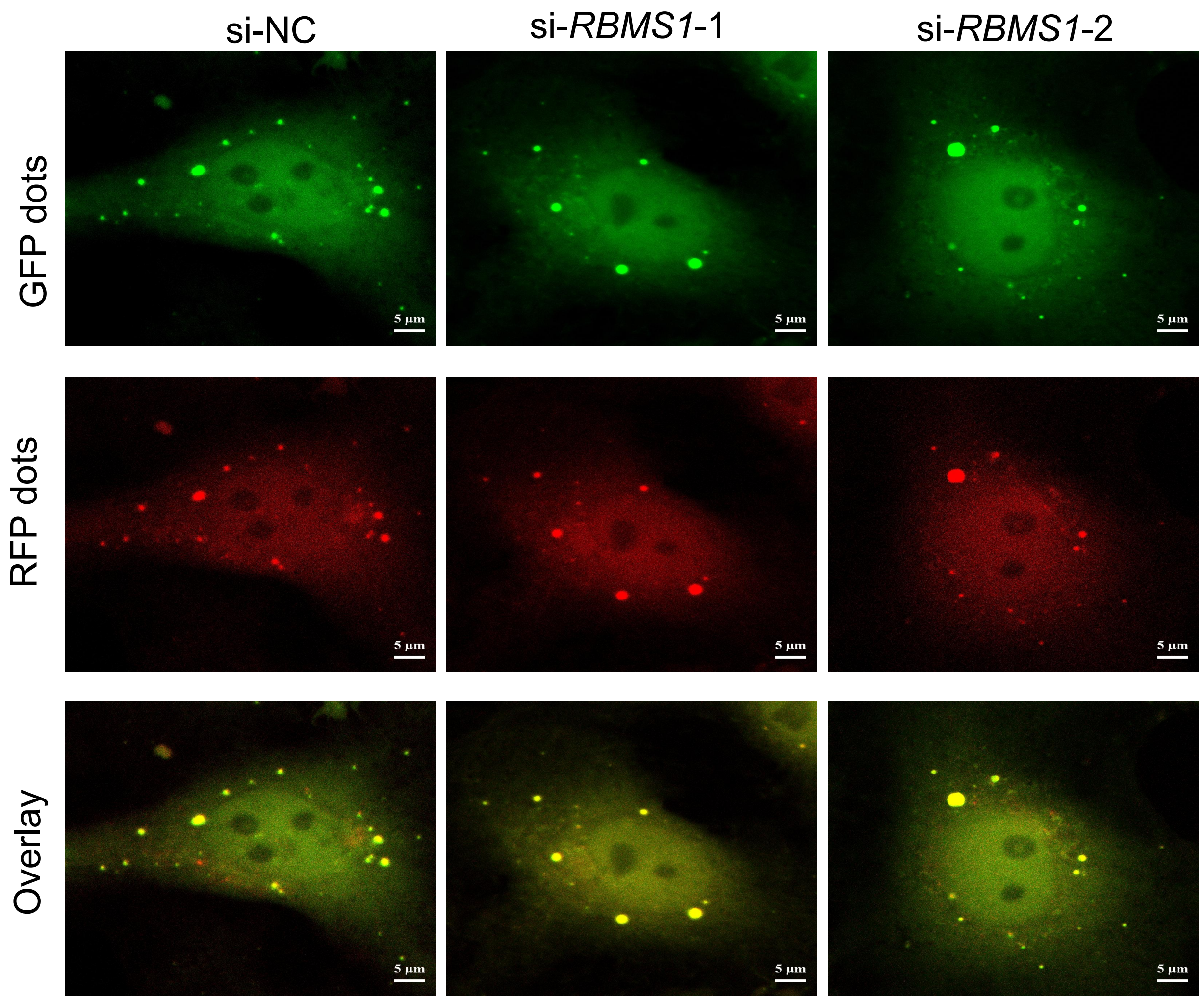

Original data of the corresponding images in Figure 8

Figure. 8B

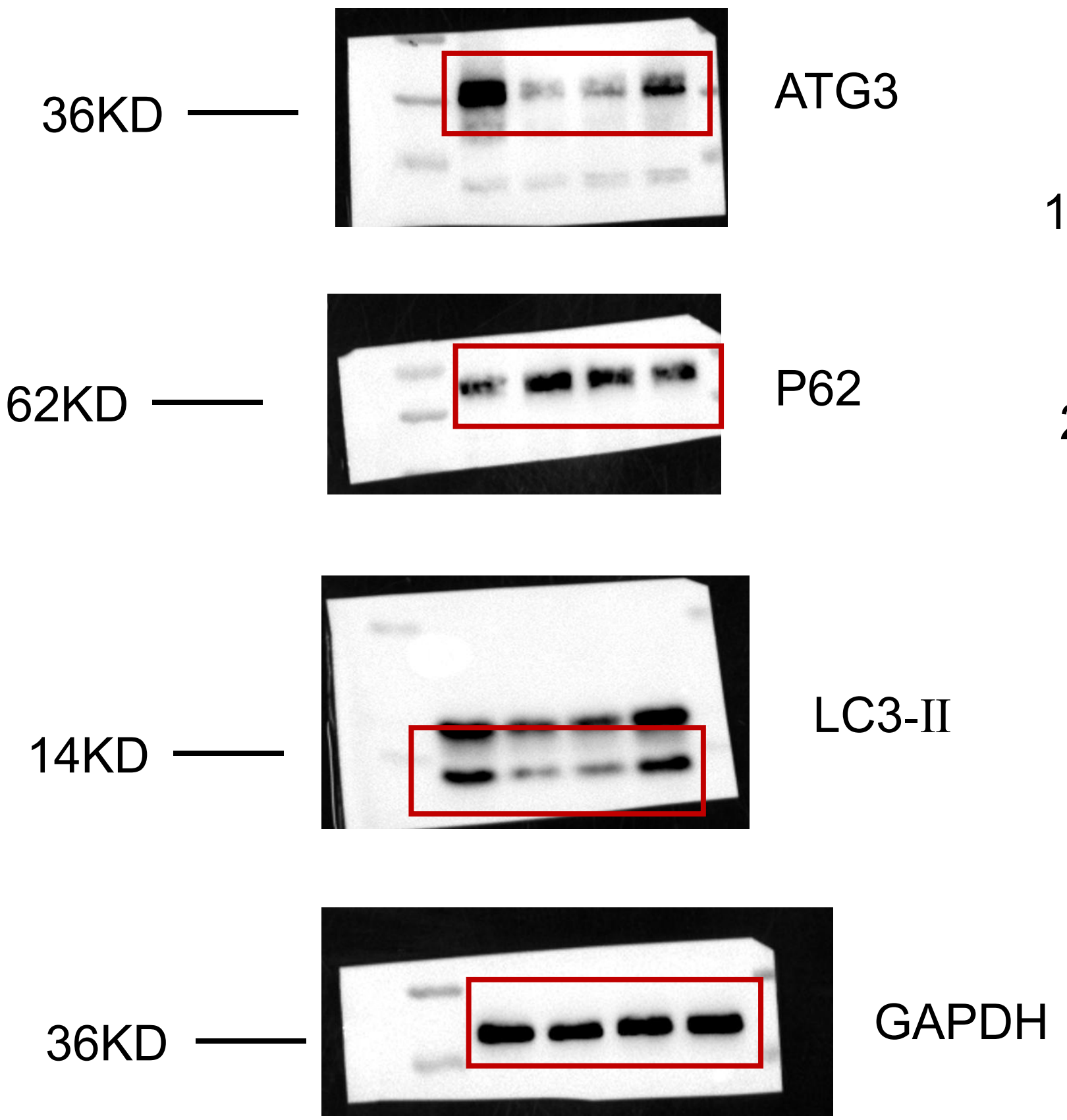

Figure. 8C

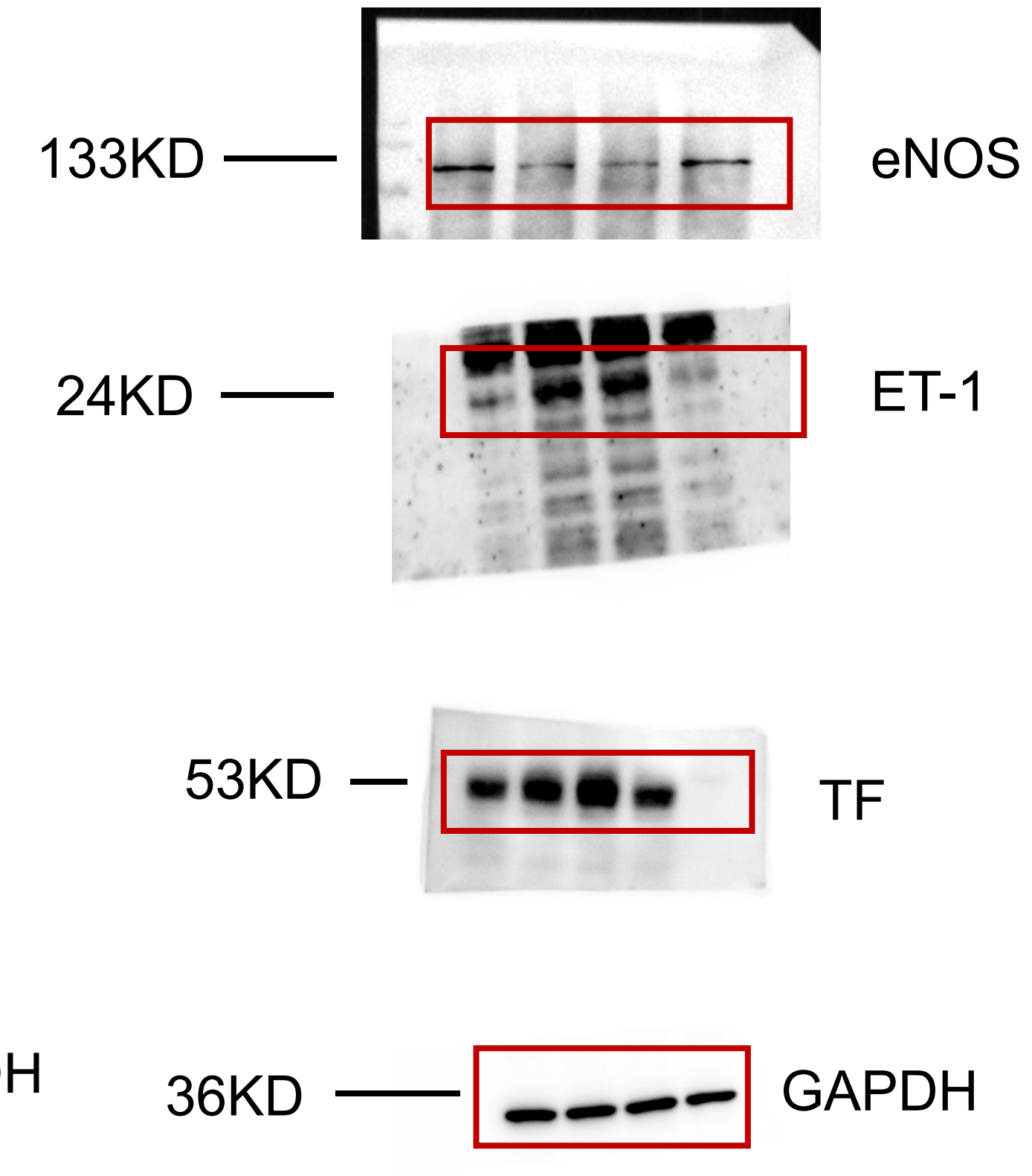

Figure. 8F

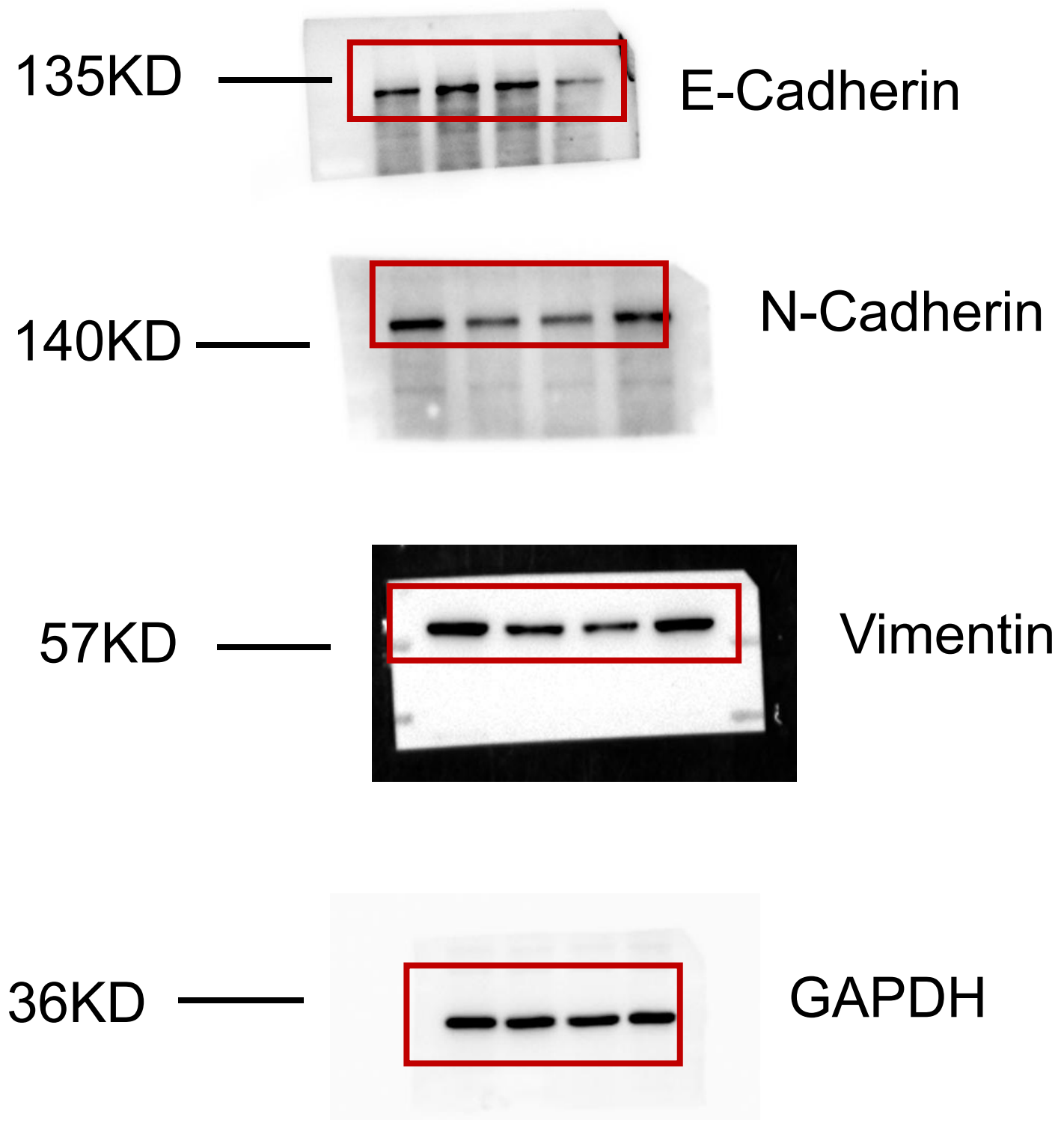

Figure. 8L

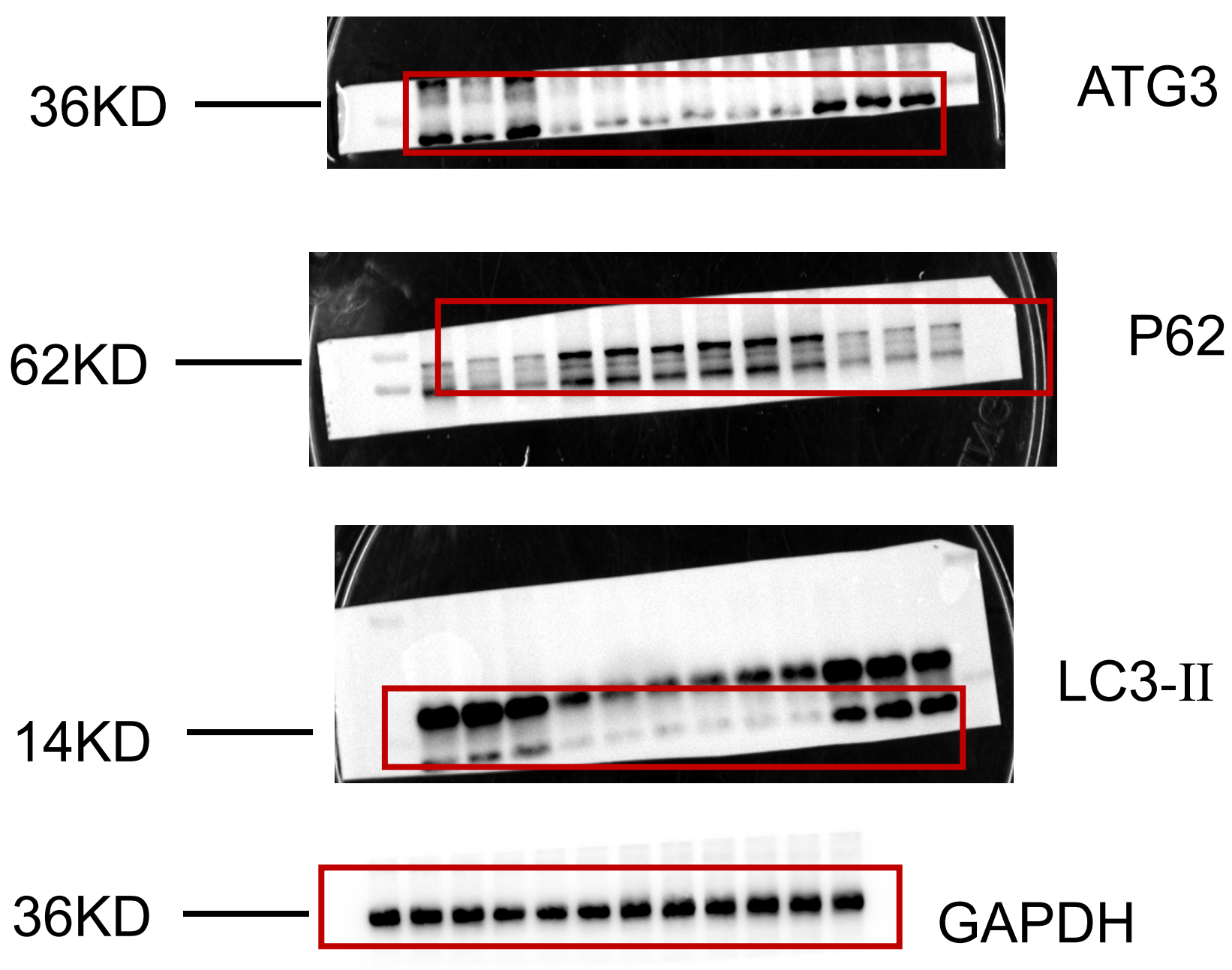

Figure. 8M

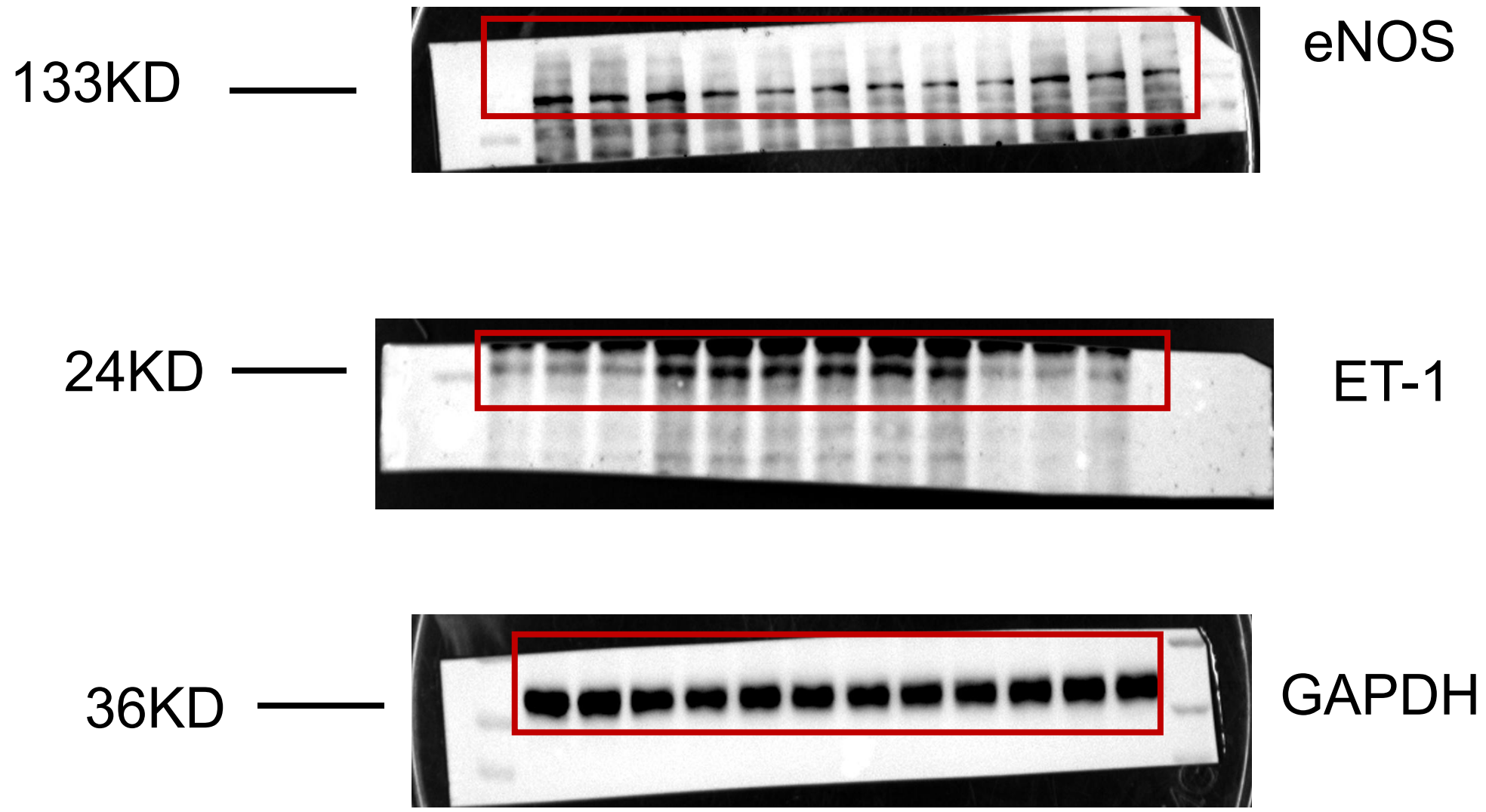

Figure. 8E

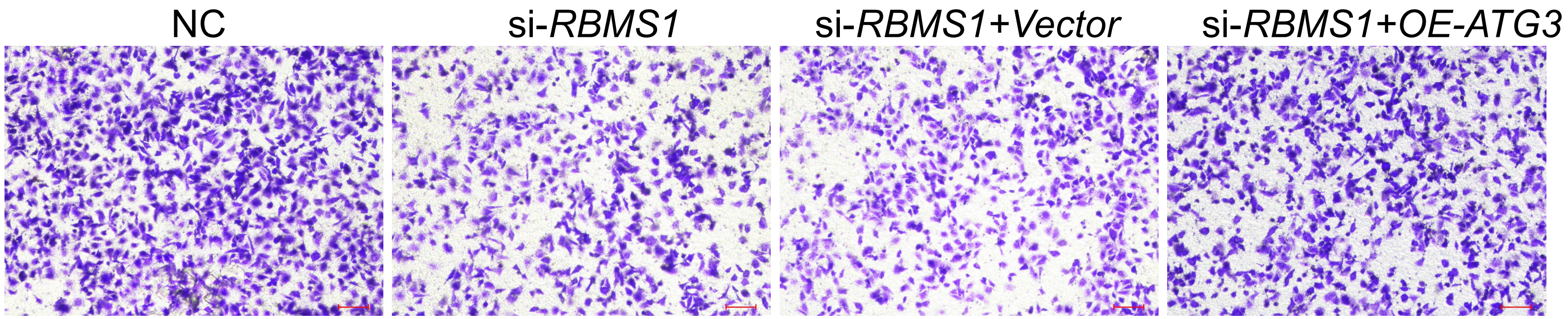

Figure. 8G

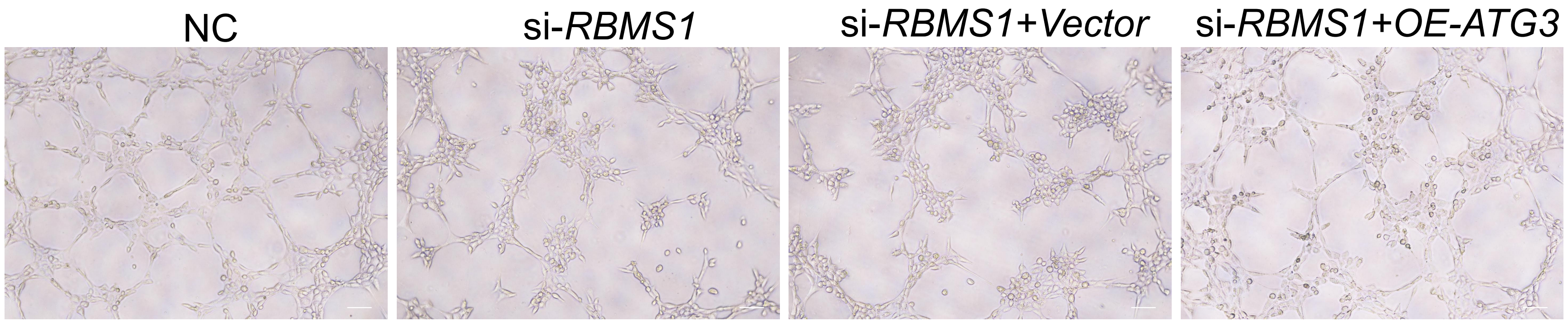

Figure. 8H

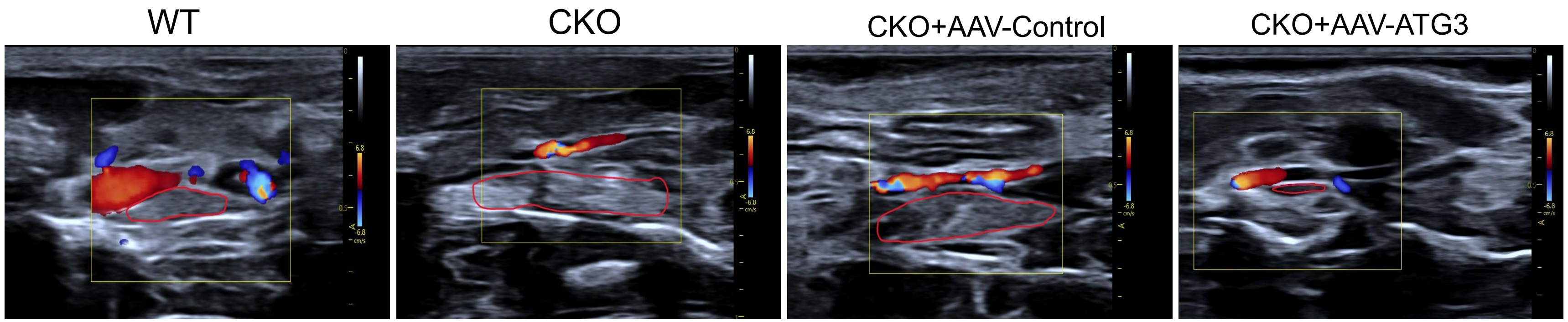

Figure. 8J

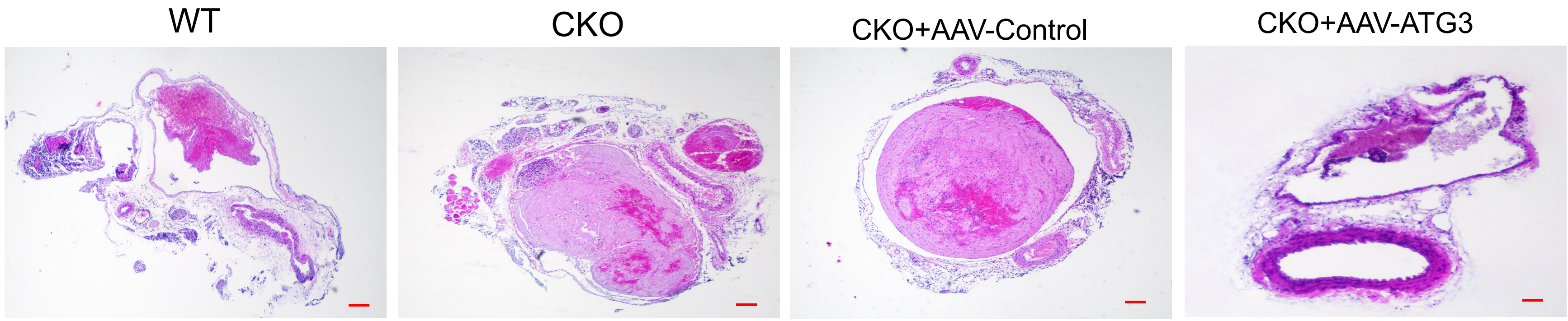

Original data of the corresponding images in Figure 9

Figure. 9E

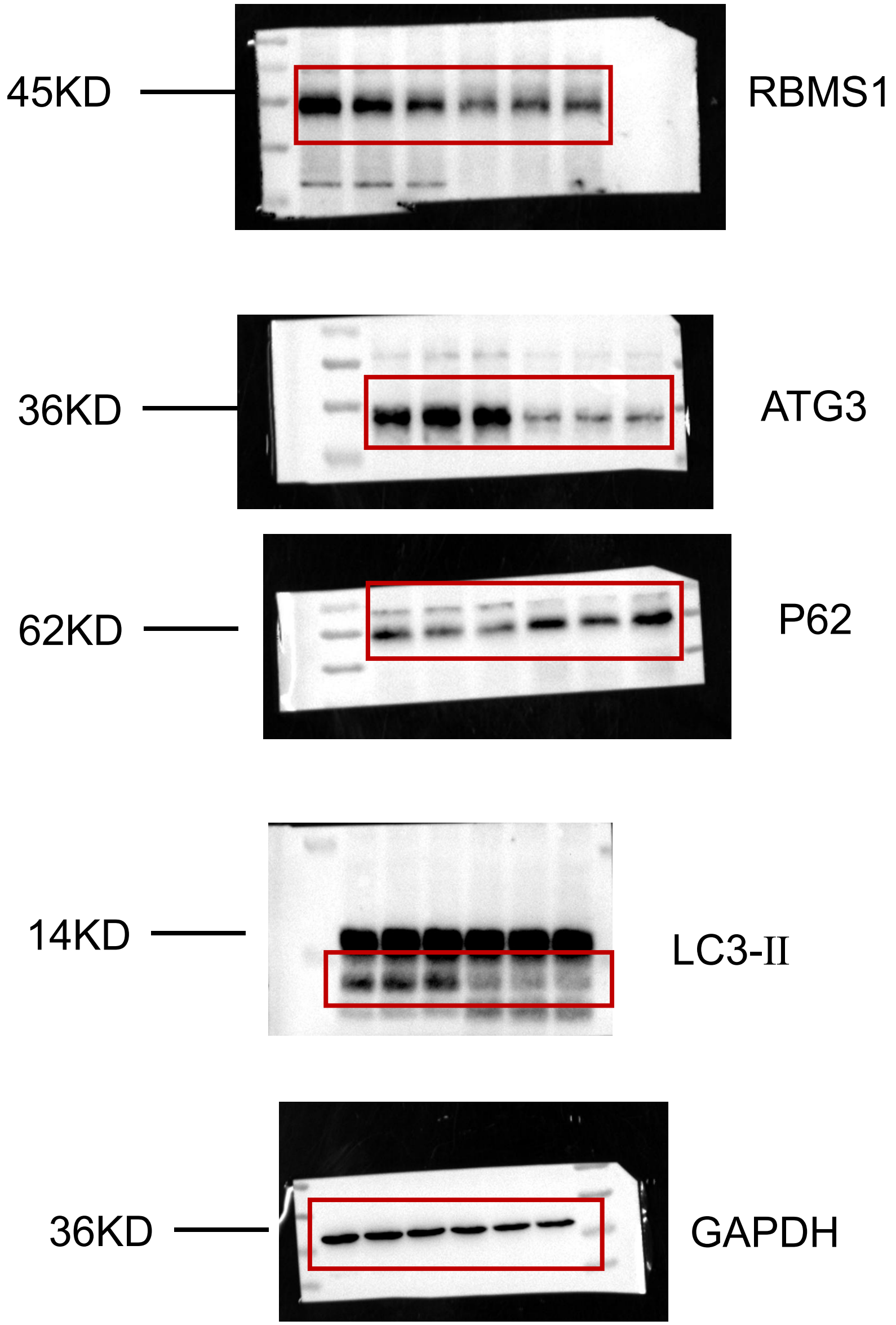

Original data of the corresponding images in Supplementary Figure1

Figure.S1A

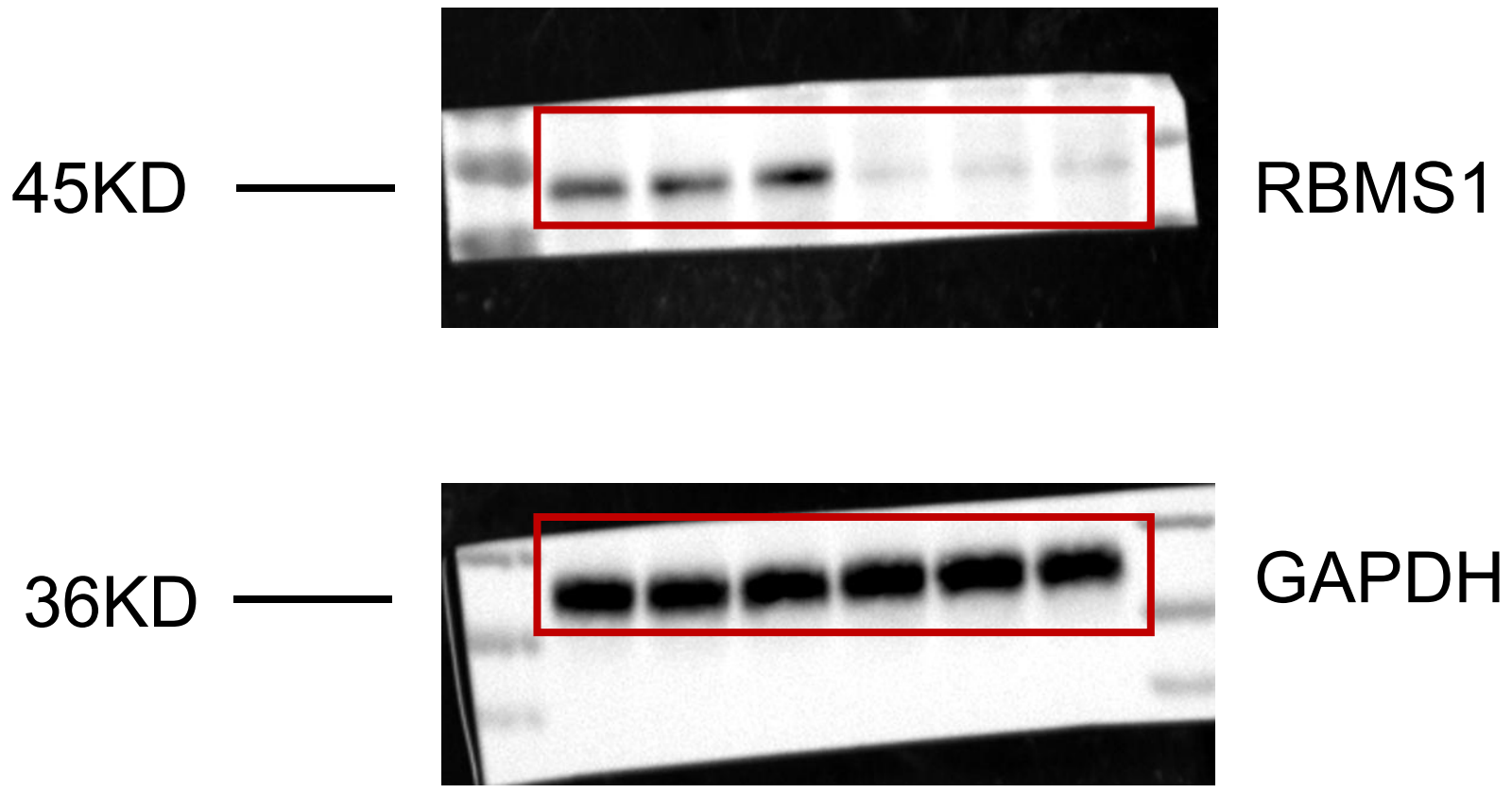

Figure.S1B

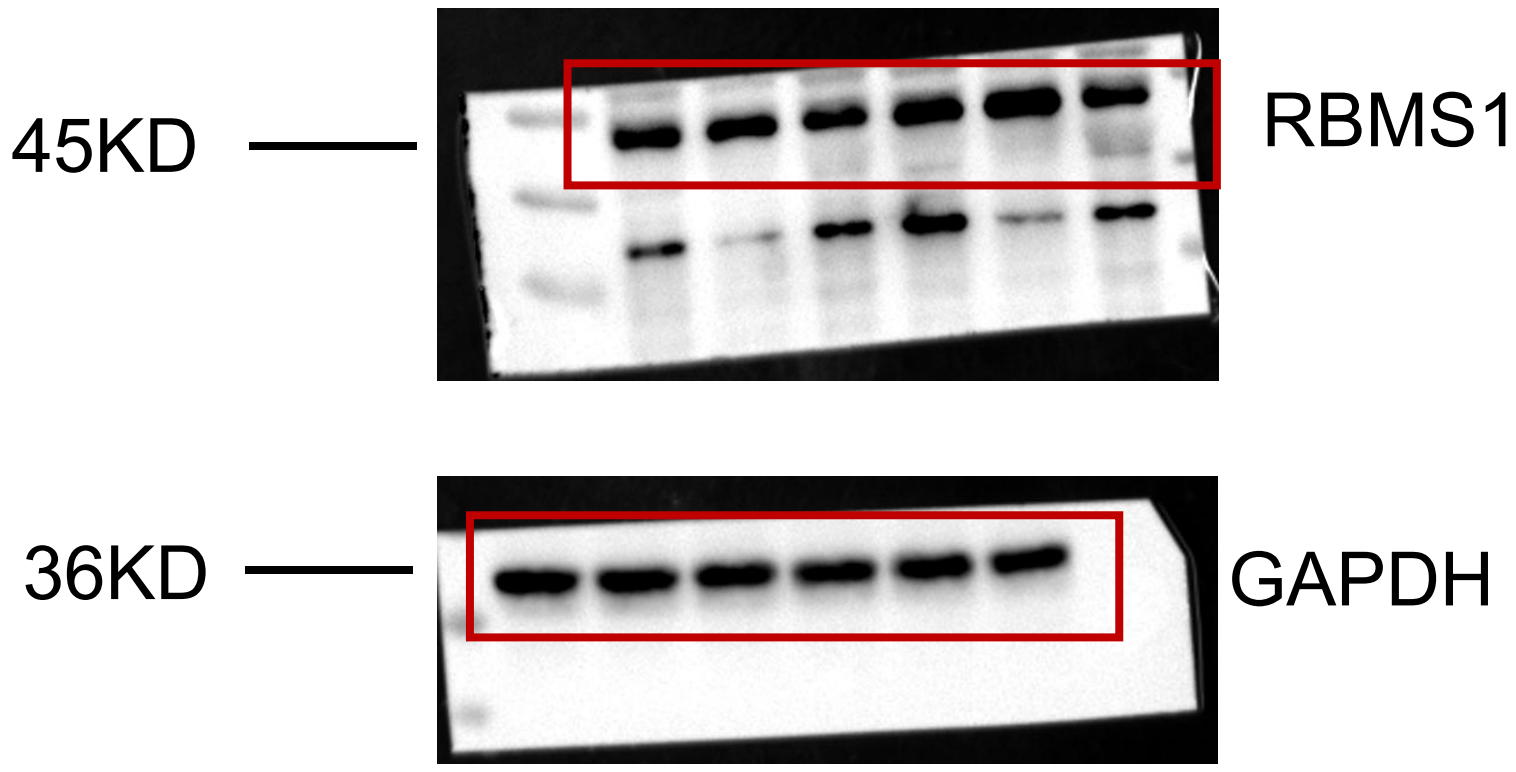

Figure.S1C

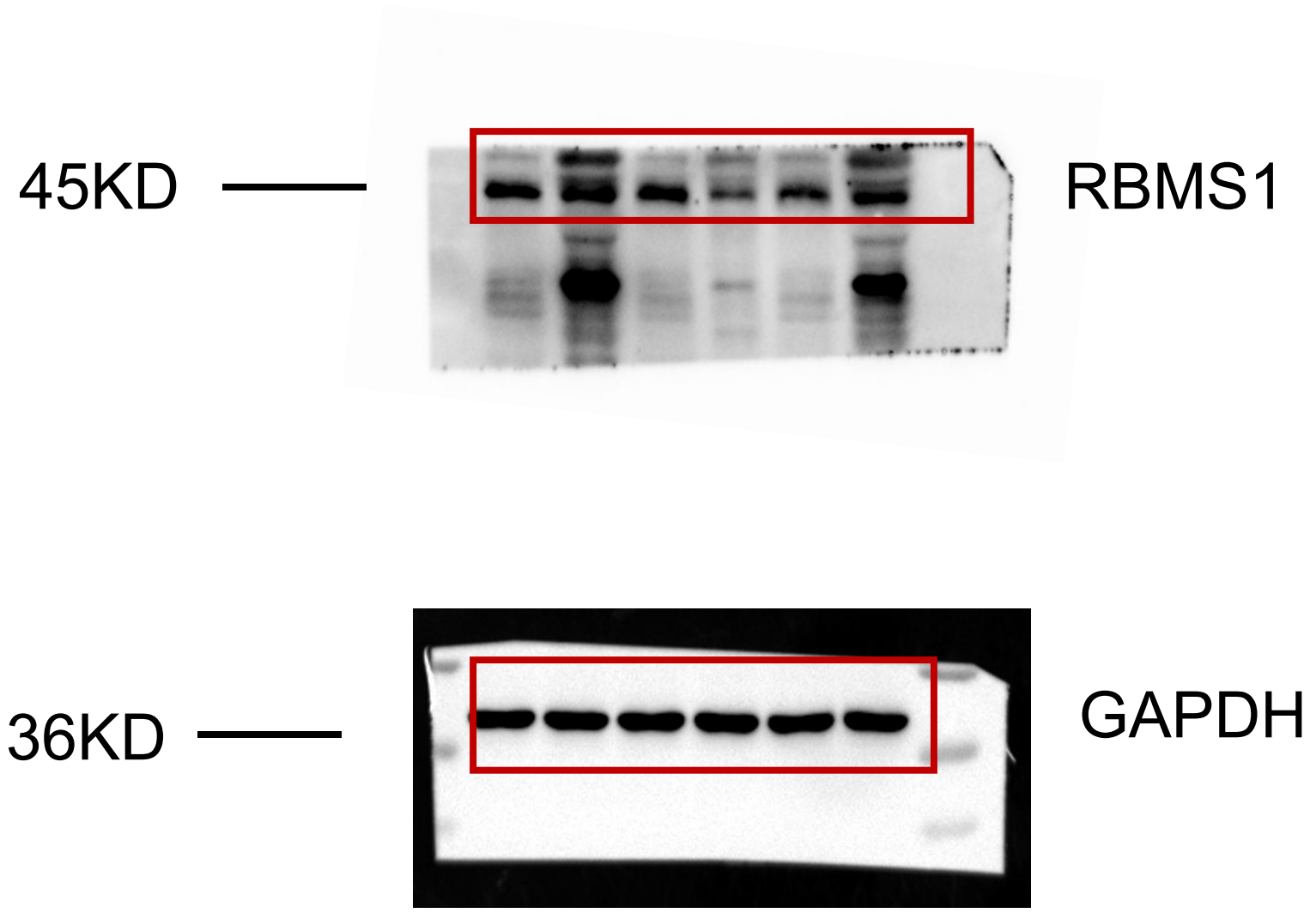

Original data of the corresponding images in Supplementary Figure 2

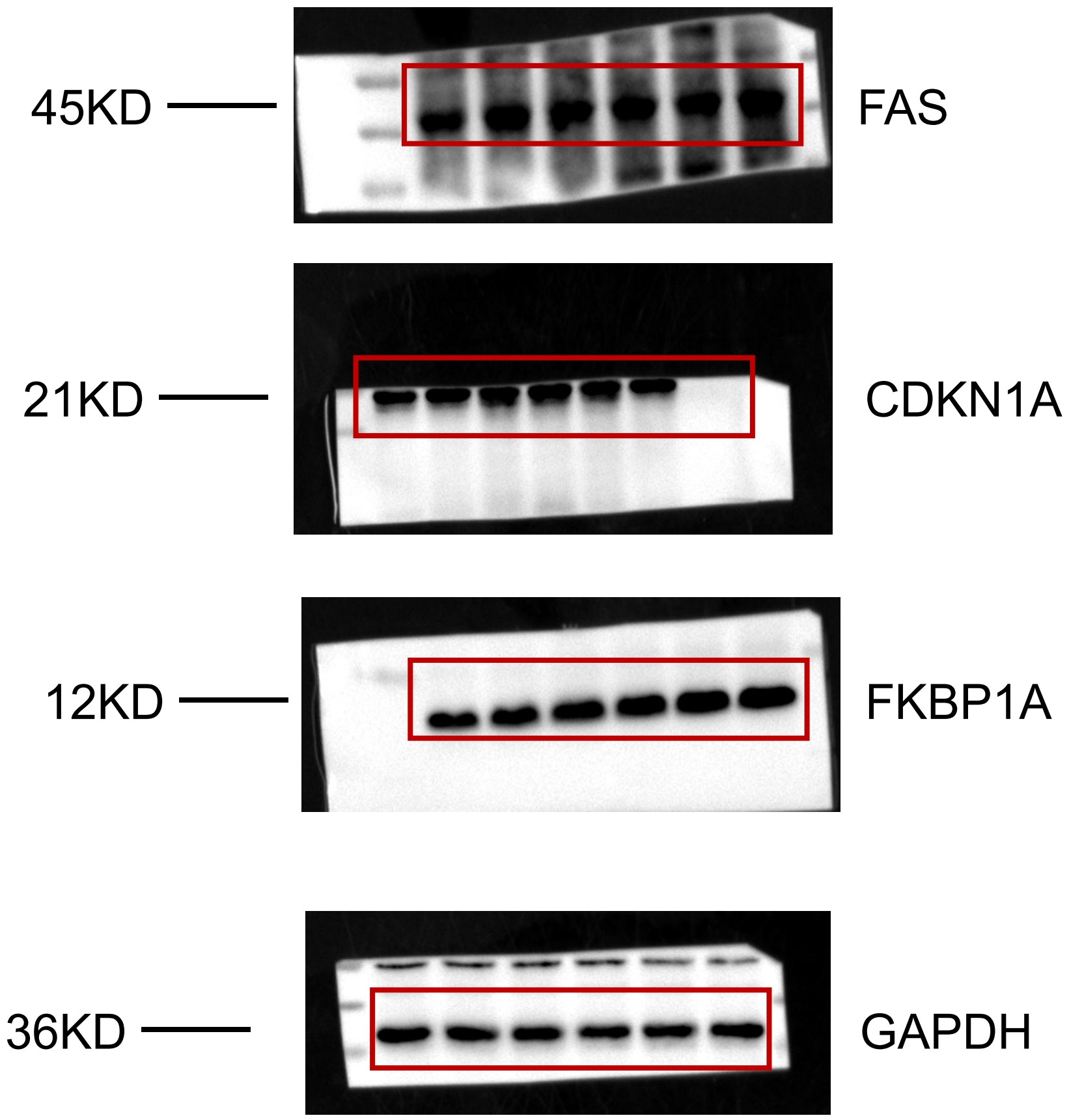

Original data of the corresponding images in Supplementary Figure 3

Figure. S3A

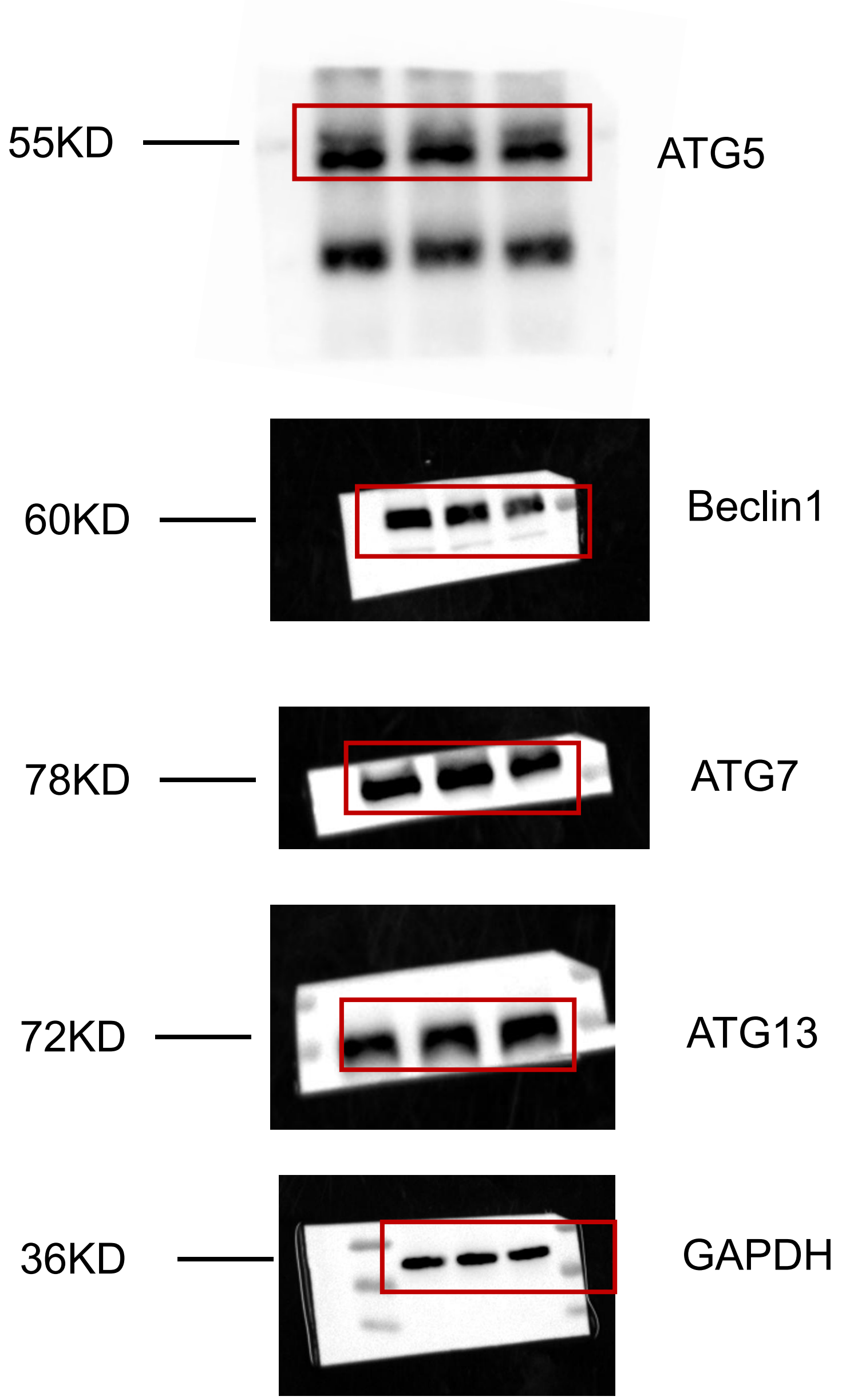

Figure.S3B

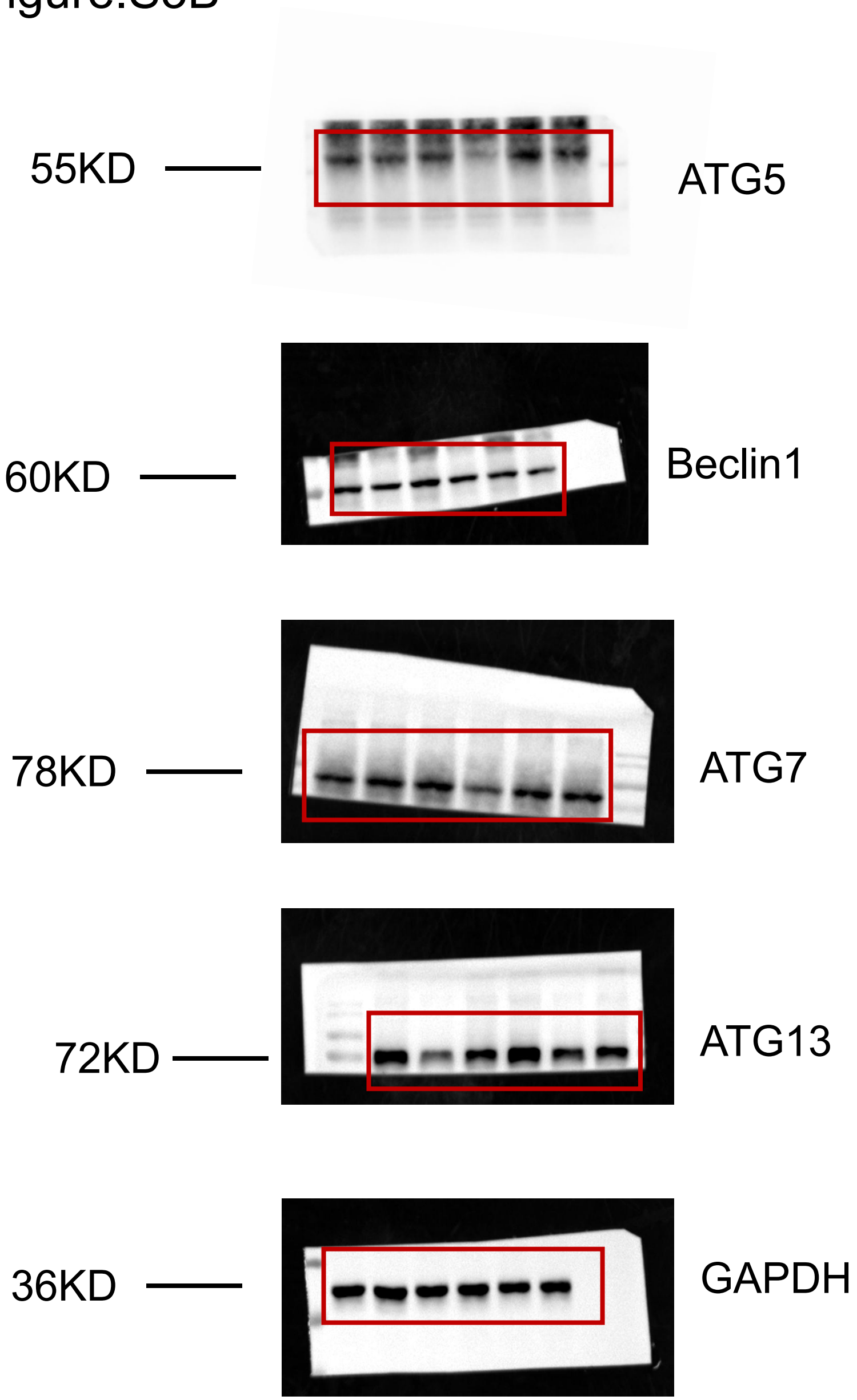

Original data of the corresponding images in Supplementary Figure 5

Figure.S5A

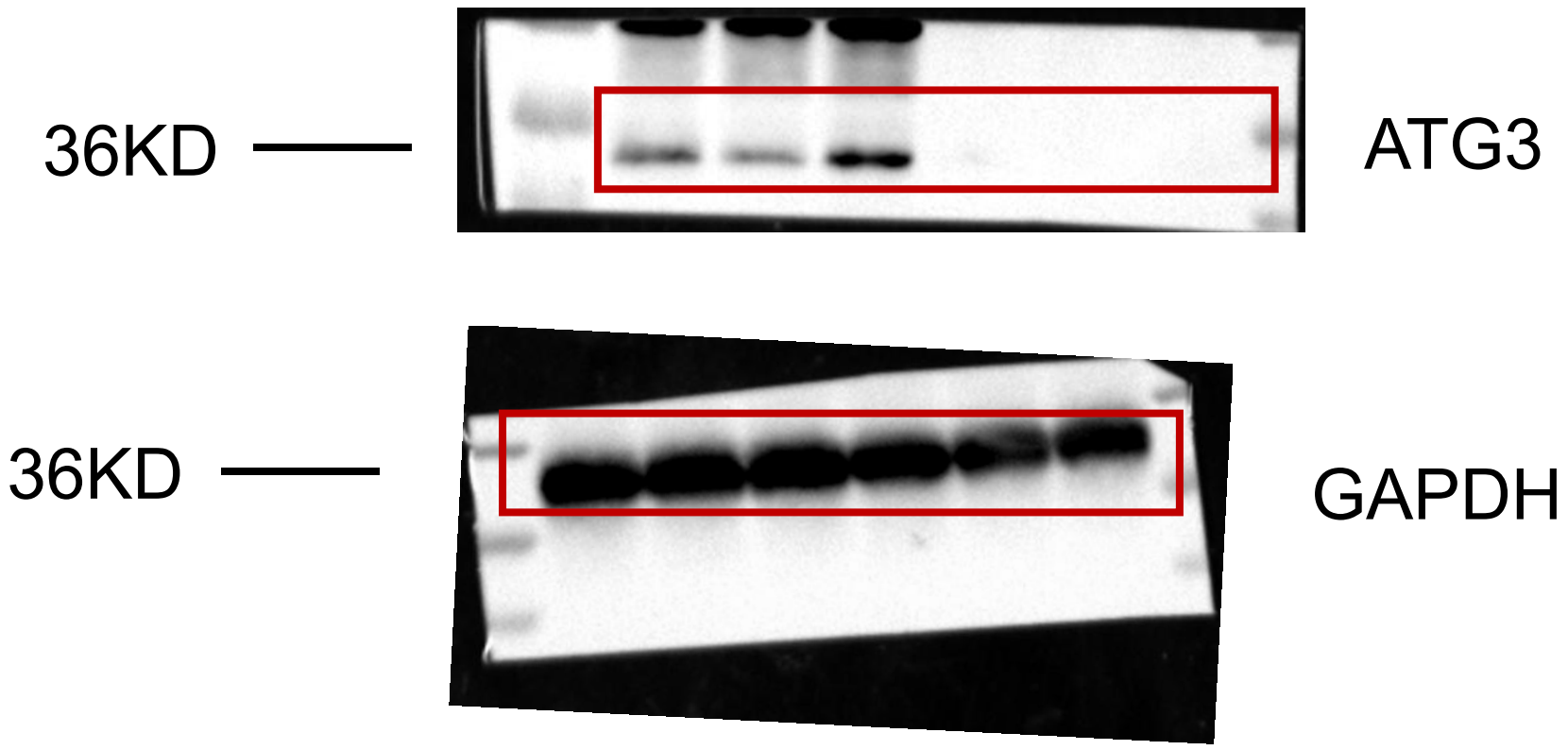

Figure.S5C

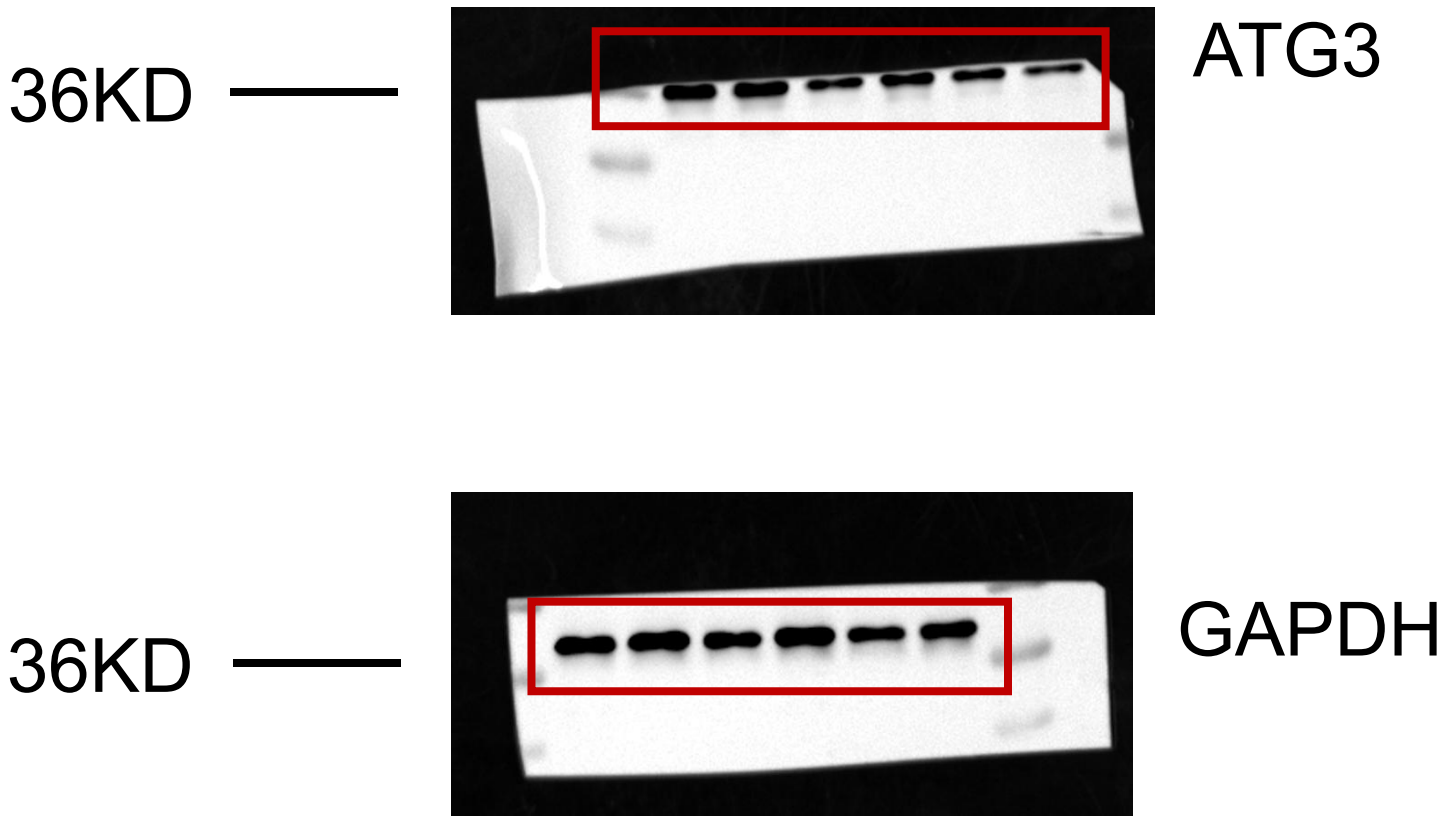

Figure.S5B

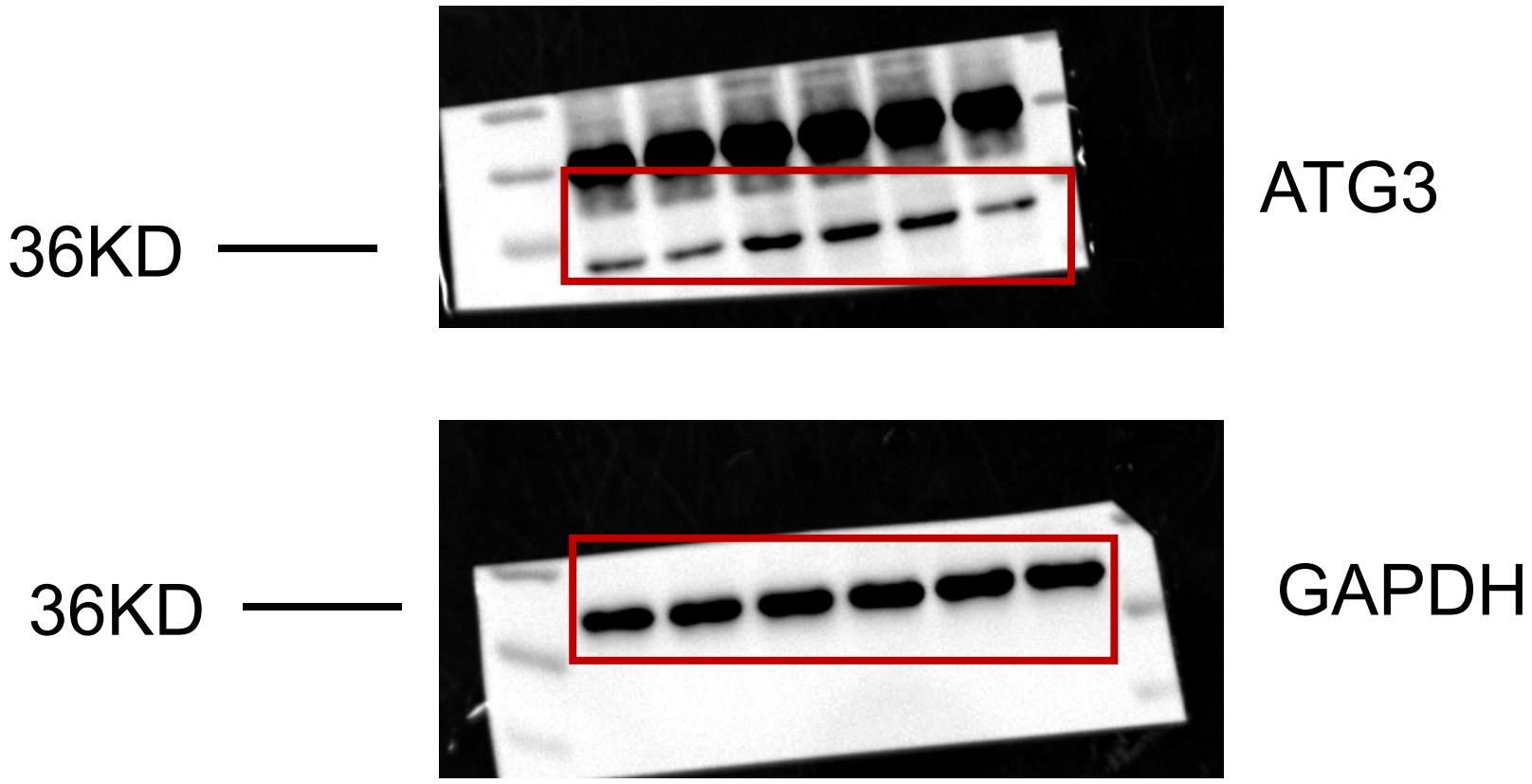

Original data of the corresponding images in Supplementary Figure 6

Figure.S6A

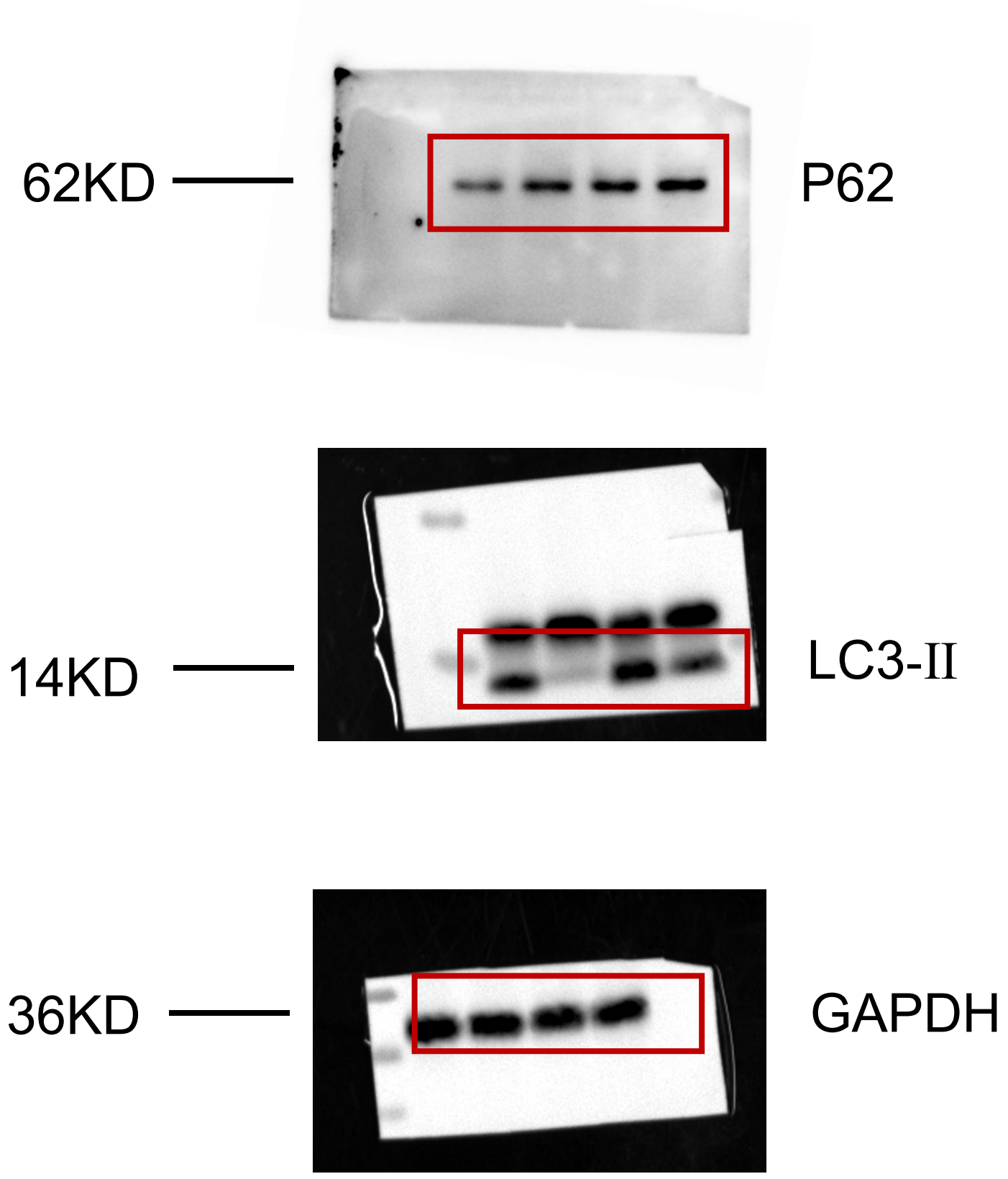

Figure.S6B

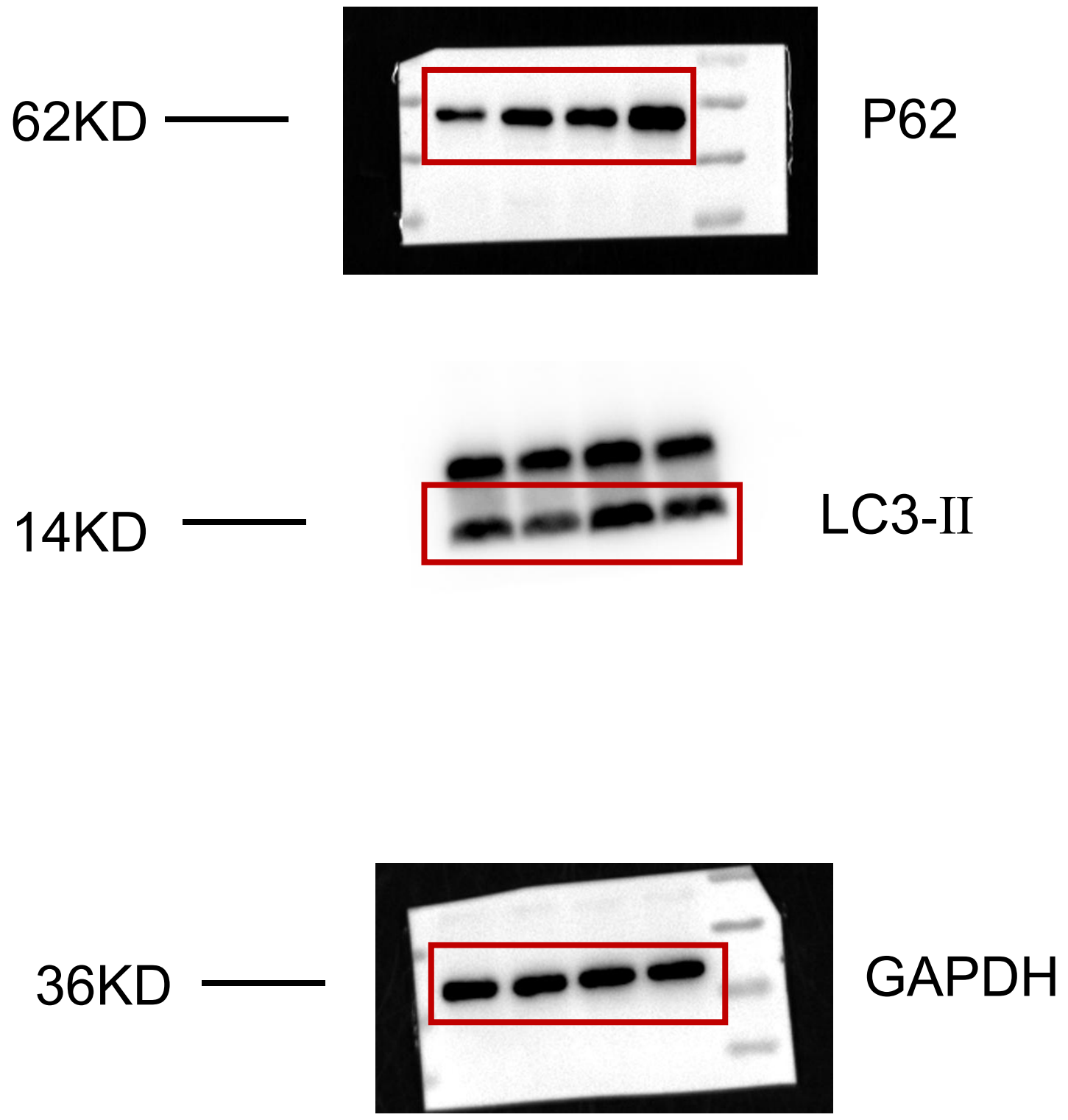

Original data of the corresponding images in Supplementary Figure 7

Figure.S7A

AAV-Control AAV-RBMS1

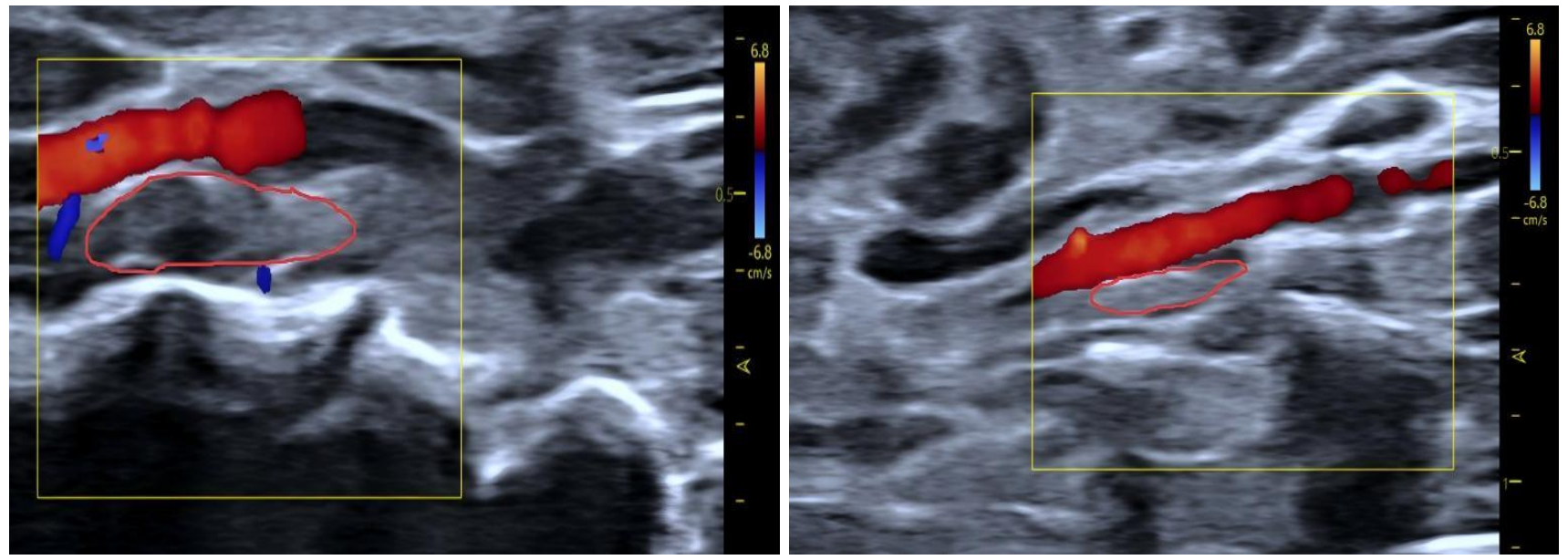

Figure.S57D

AAV-Control AAV-ATG3

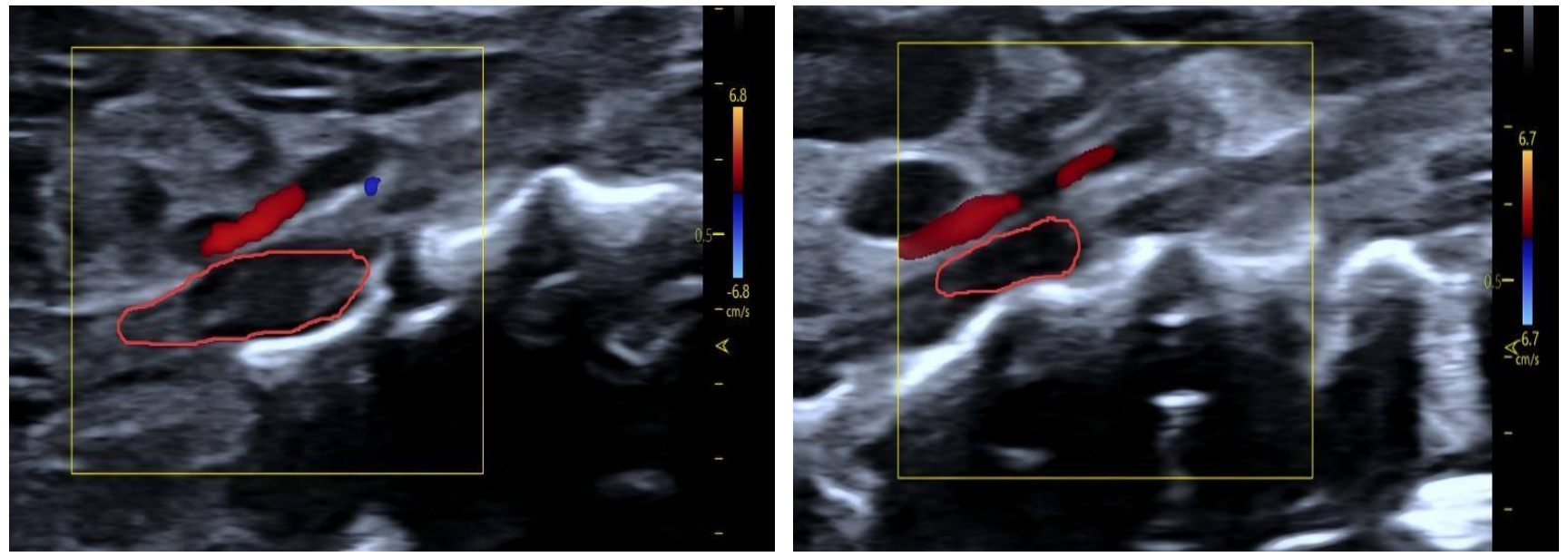

Figure.S7B

AAV-Control AAV-RBMS1

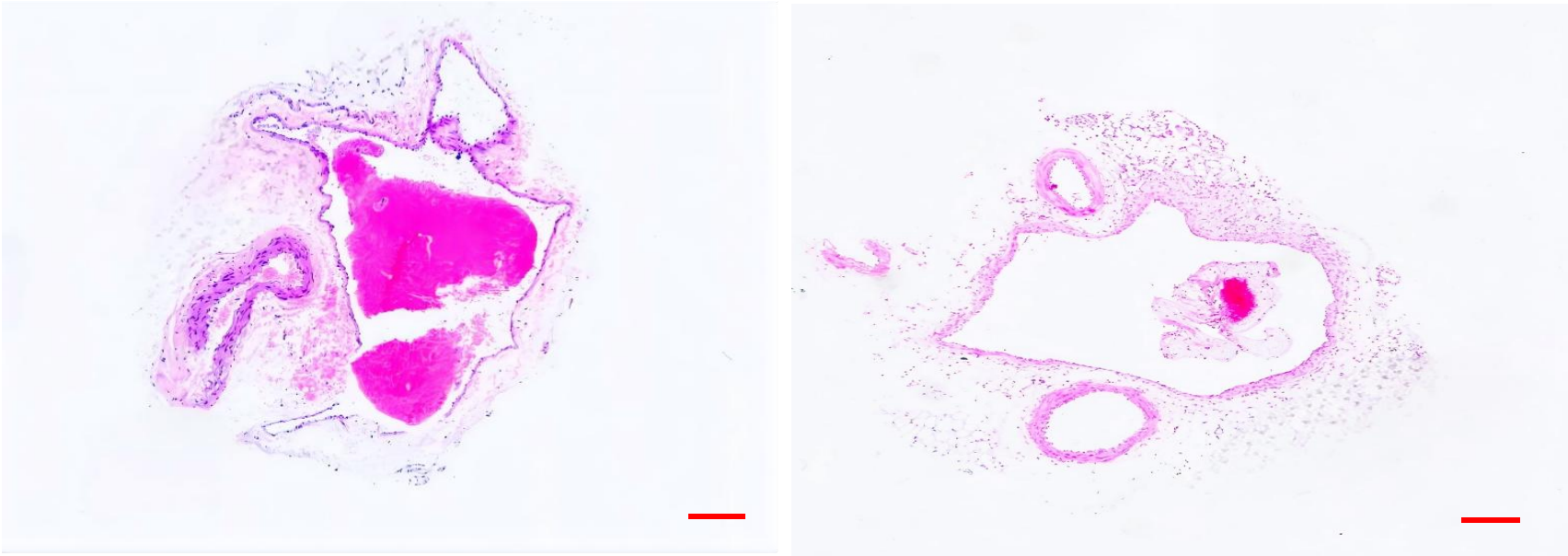

Figure.S7E

AAV-Control AAV-ATG3

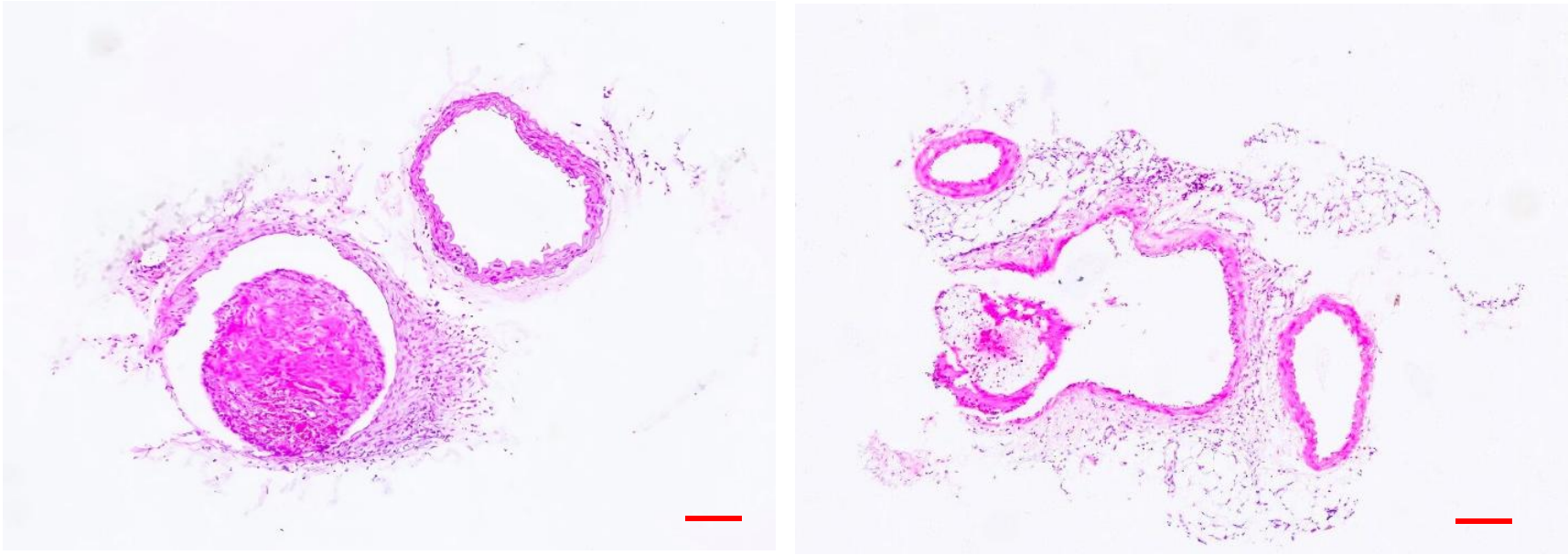

Supplement: Supplementary file 1 [file ADVS-13-e77393-s002.pdf]
