# Supplementary material for: An RNA‐Binding Protein RBMS1 Promotes Endothelial Cell Autophagy to Maintain Vascular Homeostasis and Suppress Deep Vein Thrombosis
Source: Adv Sci (Weinh). 2026 Aug 27;13(63):e77393. doi: 10.1002/advs.77393 (PMC13521309; doi:10.1002/advs.77393)
Supplement: Supplementary file 2 [file ADVS-13-e77393-s001.docx]

Supporting Information

**An RNA-binding Protein RBMS1 Promotes Endothelial Cell Autophagy to Maintain Vascular Homeostasis and Suppress Deep Vein Thrombosis**

Chu Chu^1,2^, Xinkui Liu^1,2^, Nannan Fan^1,2^, Xiaoyan Yu^1,3^, Qiaoqiao Han^1,3^, Yunhong Zhang^1,2^, Wei Li^1,2^, Yongqing Cai^1,2^, Miaomiao Zhou^1,2^, Fang Li^1,2^, Feifei Shi^1,2^, Zhen Zhang^1,2*^, Bin Wang^4*^, Xia Li^1,2,5*^

**This file includes**:

1. Supplementary Figures 1 to 8

**Figure S1.** RBMS1 protein expression was examined in endothelial-specific *Rbms1* conditional knockout mice.

(A) Western blot analysis of RBMS1 protein levels in vascular endothelial cells (VECs) isolated from the inferior vena cava of *Rbms1*^fl/fl^*Cdh5*-Cre^+^ and *Rbms1*^fl/fl^*Cdh5*-Cre^-^ mice (n= 6). (B) In non-VECs (non-vascular endothelial cells) isolated from the same vascular tissue, RBMS1 protein levels were comparable between Cre^+^ and Cre^-^ groups (n= 6). (C) Detection of RBMS1 protein levels in CD45^+^ immune cells sorted from peripheral blood mononuclear cells (PBMCs) by Western blot analysis (n= 6). Statistical significance was determined by unpaired Student’s t-test. ^****^*P*< 0.0001; ns, not significant.

**Figure S2** FAS, CDKN1A, and FKBP1A protein levels are unchanged upon endothelial Rbms1 deletion.

Representative Western blot images and densitometric quantification of FAS, CDKN1A (p21), and FKBP1A protein levels in vascular endothelial cells isolated from *Rbms1*^fl/fl^Cdh5-Cre^−^ and *Rbms1*^fl/fl^Cdh5-Cre^+^ mice. Protein expression was normalized to GAPDH. Data are presented as the mean± SEM; n= 6 mice per group. Statistical comparisons were performed using an unpaired two-sided Student’s t-test. ns, not significant.

**Figure S3** RBMS1 depletion on ATG-related protein expression *in vitro* and *in vivo*.

(A) Western blot analysis of ATG5, Beclin1, ATG7, and ATG13 protein levels in HUVECs transfected with siRBMS1 or siNC. (B) Western blot analysis of the same ATG-related proteins in vascular endothelial cells (VECs) isolated from the inferior vena cava from *Rbms1*^fl/fl^*Cdh5*-Cre^+^ and *Rbms1*^fl/fl^*Cdh5*-Cre^-^ mice. Statistical significance was determined by One-way ANOVA for A, and unpaired Student’s t-test for B. ns, not significant.

**Figure S4** RBMS1-dependent reporter mRNA stability requires ATG3 3′-UTR cis-regulatory elements.

(A, B) Reporter-mRNA decay analysis in HEK293T cells co-transfected with si-NC or si-RBMS1 and firefly luciferase reporters containing either the wild-type T1 fragment of the ATG3 3′-UTR (WT1) or the corresponding mutant sequence (MUT1). (C, D) Reporter-mRNA decay analysis in HEK293T cells co-transfected with si-NC or si-RBMS1 and firefly luciferase reporters containing either the wild-type T2 fragment of the ATG3 3′-UTR (WT2) or the corresponding mutant sequence (MUT2). Following actinomycin D treatment, firefly luciferase reporter mRNA levels were measured by qRT-PCR at the indicated time points, normalized to GAPDH, and expressed relative to the corresponding level at 0 h. Data are presented as the mean± SEM from three independent biological experiments. Reporter-mRNA decay curves were fitted using a one-phase exponential decay model, and differences were analyzed by ordinary two-way ANOVA with reporter construct and time as the two factors, followed by Šídák′s multiple-comparisons test. ^****^*P*< 0.0001, ns, not significant.

**Figure S5.** ATG3 protein expression was assessed in endothelial-specific *Atg3* conditional knockout mice.

(A) Western blot analysis was performed to assess ATG3 protein levels in VECs isolated from the inferior vena cava of *Atg3*^fl/fl^*Cdh5*-Cre^+^ and *Atg3*^fl/fl^*Cdh5*-Cre^-^ mice (n= 6). (B) The protein levels of ATG3 were detected in non-VECs isolated from the inferior vena cava of *Atg3*^fl/fl^*Cdh5*-Cre^+^ and *Atg3*^fl/fl^*Cdh5*-Cre^-^ mice (n= 6). (C) The expression level of ATG3 protein was examined by Western blot in CD45^+^ immune cells sorted from PBMCs (n= 6). Statistical significance was determined by unpaired Student’s t-test. ^****^*P*< 0.0001; ns, not significant.

**Figure S6.** P62 and LC3-II protein levels in different groups.

(A) Representative Western blot images and densitometric quantification of p62 and LC3-II protein levels in vascular endothelial cells isolated from DVT mice of the indicated genotypes, with or without chloroquine (CQ) treatment. Protein expression was normalized to GAPDH. Data were obtained from six mice per group (n= 6). Data were analyzed by two-way ANOVA, followed by Šídák′s multiple-comparisons test with multiplicity adjustment. The interaction statistics were as follows: p62, F(1, 20) = 11.17, *P*= 0.0032; LC3-II, F(1, 20) = 29.74, *P*< 0.0001. ^****^*P*< 0.0001 for the indicated post hoc comparison. (B) Representative Western blot images and densitometric quantification of p62 and LC3-II protein levels in HUVECs transfected with si-NC or si-RBMS1, with or without CQ treatment. Protein expression was normalized to GAPDH. Data were obtained from three independent biological replicates per group (n= 3). Data were analyzed by two-way ANOVA, followed by Šídák′s multiple-comparisons test with multiplicity adjustment. The interaction statistics were as follows: p62, F(1, 8)= 8.528, P= 0.0193; LC3-II, F(1, 8)= 105.7, *P*< 0.0001. ^****^*P*< 0.0001 for the indicated post hoc comparison.

**Figure S7** Overexpression of RBMS1 or ATG3 suppresses thrombus formation *in vivo*.

(A) Representative Doppler ultrasound images of thrombi in DVT model mice from the empty-vector control group (AAV-Control) and the RBMS1 overexpression group (AAV-RBMS1). (B) H&E staining of thrombus sections from the AAV-Control and AAV-RBMS1 groups (magnification: ×40; scale bar: 100 μm). (C) Quantification of thrombus length and weight in AAV-Control and AAV-RBMS1 groups (n = 6 per group). (D) Representative Doppler ultrasound images of thrombi in DVT model mice from the AAV-Control and ATG3 overexpression group (AAV-ATG3). (E) H&E staining of thrombus sections from the AAV-Control and AAV-ATG3 groups (magnification: ×40; scale bar: 100 μm). (F) Quantification of thrombus length and weight in AAV-Control and AAV-ATG3 groups (n = 6 per group). Data are presented as mean± SEM. Statistical significance was determined by unpaired Student’s t-test. ^***^*P*< 0.001, ^****^*P*< 0.0001.

**Figure S8** VWF release is regulated by the RBMS1-ATG3 axis in vascular endothelial cells.

(A) VWF levels were measured by ELISA in the culture supernatants of vascular endothelial cells isolated from *Atg3*^fl/fl^Cdh5-Cre^+^ mice and their *Atg3*^fl/fl^Cdh5-Cre^−^ littermate controls. (B) VWF levels were measured by ELISA in the culture supernatants of vascular endothelial cells isolated from WT, CKO, CKO+ AAV-Control, and CKO+ AAV-ATG3 mice. Each dot represents an individual mouse. Data are presented as the mean± SEM (n= 6 mice per group). Statistical significance was assessed using an unpaired two-tailed Student’s t-test in (A) and two-way ANOVA followed by Šídák′s multiple-comparisons test in (B). ^****^*P*< 0.0001.
